# A systematic review and meta-analysis of effects of menopausal hormone therapy on cardiovascular diseases

**DOI:** 10.1038/s41598-020-77534-9

**Published:** 2020-11-26

**Authors:** Ji-Eun Kim, Jae-Hyuck Chang, Min-Ji Jeong, Jaesung Choi, JooYong Park, Chaewon Baek, Aesun Shin, Sang Min Park, Daehee Kang, Ji-Yeob Choi

**Affiliations:** 1 Department of Biomedical Sciences, Seoul National University Graduate School, 103 Daehak-ro, Jongno-gu, Seoul, 03080 Korea; 2 BK21plus Biomedical Science Project, Seoul National University College of Medicine, Seoul, Korea; 3 Northeastern University Bouve College of Health Sciences School of Pharmacy, Boston, MA USA; 4 Department of Preventive Medicine, Seoul National University College of Medicine, Seoul, Korea; 5 Cancer Research Institute, Seoul National University, Seoul, Korea; 6 Department of Innovative Medical Science, Seoul National University College of Medicine, Seoul, Korea; 7 Department of Family Medicine, Seoul National University College of Medicine, Seoul, Korea; 8 Institute of Environmental Medicine, Seoul National University Medical Research Center, Seoul, Korea; 9 Institute of Health Policy and Management, Seoul National University Medical Research Center, Seoul, Korea

**Keywords:** Cardiovascular diseases, Epidemiology

## Abstract

A systematic review and meta-analysis of randomized controlled trials (RCTs) and observational studies was conducted to assess the association between menopausal hormone therapy and cardiovascular disease. The PubMed and EMBASE databases were searched for articles published from 2000 to 2019, using review methods based on a previous Cochrane review. Quality assessment of RCTs and observational studies was conducted using the Jadad scale and the Newcastle–Ottawa Scale, respectively. A total of 26 RCTs and 47 observational studies were identified. The study populations in the RCTs were older and had more underlying diseases than those in the observational studies. Increased risks of venous thromboembolism [summary estimate (SE), 95% confidence interval (CI): RCTs, 1.70, 1.33–2.16; observational studies, 1.32, 1.13–1.54] were consistently identified in both study types, whereas an increased risk of stroke in RCTs (SE: 1.14, 95% CI: 1.04–1.25) and a decreased risk of myocardial infarction in observational studies (SE: 0.79, 95% CI: 0.75–0.84) were observed. Differential clinical effects depending on timing of initiation, underlying disease, regimen type, and route of administration were identified through subgroup analyses. These findings suggest that underlying disease and timing of initiation should be carefully considered before starting therapy in postmenopausal women.

## Introduction

Previous studies have reported that while premenopausal women have a lower risk of developing cardiovascular disease (CVD) than men, postmenopausal women have a higher risk^1,2^. It has been suggested that metabolic changes due to estrogen depletion after menopause lead to an increased CVD risk in postmenopausal women^3–5^. Experimental studies have identified several protective mechanisms of estrogen against CVD, which include increasing angiogenesis and vasodilation, and reducing fibrosis and oxidative stress^6^. Menopausal hormone therapy (MHT) was suggested to contribute to the reduction of CVD risk based on the hypothesis of cardioprotection by estrogen^7,8^. Many randomized controlled trials (RCTs) and observational studies have investigated the association between MHT and CVD risk; however, there have been inconsistent results among studies. Early observational studies have reported beneficial effects of MHT on CVD, whereas large RCTs, such as the Women’s Health Initiative (WHI) and the Heart and Estrogen/Progestin Replacement Study (HERS) have not^7–11^. However, some major limitations of the RCTs are that the women were older, had initiated MHT late after menopause, and either had CVD risk factors at baseline or had a history of a CVD event^10–12^. Since the publication of the WHI report, several studies have reevaluated the risk profile of MHT^13–15^. Despite that, controversies remain regarding CVD-related risks and benefits of MHT. Therefore, emphasis has been placed on the necessity of additional studies that evaluate the following factors: dose of estrogen, route of administration, timing after menopause, duration of use, other hormone effects, pre-existing pathology, and age^3^.

The Cochrane library conducted a meta-analysis of the association of MHT with specific CVD outcomes in RCTs. They conducted subgroup analyses based on the timing of MHT after menopause (timing hypothesis), but the study did not consider other confounding factors^16^. Another meta-analysis of RCTs examining the timing hypothesis for MHT and CVD risk supported the importance of the timing of initiation of MHT, and concluded that MHT may have beneficial effects on mortality and CVD events in younger menopausal women^17^. The previous meta-analysis studies of RCTs were limited to the timing hypothesis, and did not include other confounding factors that could affect the association between MHT and CVD. The most recent study was a systematic review of individual studies on the effects of timing, routes of administration, duration, and dose of MHT on CVD. However, that review was based on the findings of observational studies, and did not conduct pooled analyses of results owing to the diversity of the studies^18^. In addition, a previous meta-analysis of RCTs reported that inconsistent findings between the study designs may be due to the differences in the characteristics of the study populations, methodologic limitations of observational studies, and lower event rates and shorter duration of treatments in RCTs^19^.

Therefore, it is necessary to consistently examine the results of RCTs and observational studies by conducting various subgroup analyses and by comparing the characteristics of the included study populations. The aim of this study was to assess the association between MHT and CVD outcomes, and to compare the results of RCTs and observational studies through a systematic review and meta-analysis of RCTs and observational studies, respectively.

## Methods

### Search strategy and selection criteria

A literature search was conducted according to each study design (RCTs and observational studies) using the following search terms: (“cardiovascular diseases” OR “cerebrovascular disorder” OR “all-cause death” OR “cardiovascular death” OR “death” OR “mortality”) AND (“hormone replacement therapy” OR “hormone therapy” OR “menopausal hormone therapy” OR “postmenopause”). Detailed search terms can be found in Supplementary File 1. The PubMed and EMBASE databases were searched to identify relevant articles published between January 2000 and December 2019. In the Cochrane review, studies published before 2000 were assessed as having a higher risk of bias than articles published after 2000^16^. Thus, the current study included only studies published after 2000.

The study selection criteria for the RCTs were based on the Cochrane reviews by Boardman et al.^16^. The same criteria were applied to the observational studies. After removing duplicates, exclusion criteria were separately applied to the remaining RCTs and observational studies. Only original articles of human studies published in English were included. Studies that did not report about relevant exposure or outcomes, or included either an ineligible population (premenopausal women or cancer survivors) or a duplicated study population, or had ineligible data to conduct the meta-analysis were excluded. Studies with an insufficient follow-up duration of less than 6 months in the RCTs or those with an ineligible study design (cross-sectional design) were also excluded. Reference lists of the relevant studies were manually screened to include more articles in our meta-analysis.

### Data extraction and quality assessment

Two authors (JHC and MJJ) participated in study selection and data extraction. Two other authors (JEK and JYC) checked and reviewed the data in two steps. We extracted the data as follows: (1) characteristics of the included studies and populations, including author, year of publication, study design, follow-up duration, sample size, ethnicity, age at baseline, and underlying diseases; (2) exposure, including initiation of MHT after menopause, regimen type [estrogen only (E only) or combined estrogen-progesterone (combined EP)], route of administration, and duration, and recency of MHT; (3) outcomes, including all-cause death, cardiovascular death, stroke, venous thromboembolism (VTE), pulmonary embolism (PE), myocardial infarction (MI), coronary heart disease (CHD), angina, and revascularization; and (4) the effect estimates of the association between MHT and outcomes, such as hazard ratio, relative risk, odds ratio, 95% confidence interval (CI), the number of exposed/non-exposed of MHT, and each event. Multivariable adjusted estimates were primarily extracted to reduce any confounding effects. If a study did not include the estimated values, the combined estimates were calculated based on the original estimates or the number of exposed/non-exposed, and each event was extracted as it was for the meta-analysis. Supplementary Tables S1–S4 provide details about the RCTs and observational studies included in the meta-analysis.

Representative studies were selected from one or more trials or studies, prioritizing the following selection criteria: (1) longest follow-up duration, (2) largest number of outcomes, or (3) largest number of participants. Detailed information on the selected representative RCTs and observational studies is presented in Supplementary Tables S5 and S6, respectively.

Quality assessment was conducted using the Jadad scale for RCTs, and the Newcastle–Ottawa Scale (NOS) for observational studies^20,21^. The Jadad scale consists of three domains: randomization (0–2 points), blinding (0–2 points), and an account of all patients (0–1 point). We classified the quality of RCTs as good (4–5 points), fair (3 points), or poor (0–2 points). The NOS is based on three domains: selection (0–4 stars), comparability (0–2 stars), and exposure for cohort and nested case–control studies or outcomes for case–control studies (0–3 stars). The NOS, a star system, was converted to the Agency for Healthcare Research and Quality standards. The thresholds for assessing quality were as follows: (1) good: selection (3 or 4 stars) AND comparability (1 or 2 stars) AND outcome/exposure (2 or 3 stars); (2) fair: selection (2 stars) AND comparability (1 or 2 stars) AND outcome/exposure (2 or 3 stars); and (3) poor: selection (0 or 1 star) OR comparability (0 star) OR outcome/exposure (0 or 1 star).

### Statistical analysis

The association between MHT and each CVD outcome was evaluated using summary estimates (SE) and corresponding 95% CIs. Heterogeneity among the included studies was assessed by the I^2^ index and Q statistics. We employed a fixed-effects model if the I^2^ was < 30% and the *P*-value by Q statistic was > 0.05. If not, a random-effects model was used. In both RCTs and observational studies, we conducted subgroup analyses by regimen type (E only and combined EP), duration of MHT (< 5 and ≥ 5 years), timing of initiation of MHT (early: age < 60 years or initiation within 10 years since menopause; late: age ≥ 60 years or initiation after 10 years since menopause), and underlying diseases (with or without). Subgroup analyses of observational studies were conducted by route of administration (oral and non-oral), study design (cohort, nested case–control, and case–control), recency of MHT (past and current), and study quality (good/fair and poor). We defined the timing of initiation of MHT based on the criteria of the timing hypothesis presented in a previous RCT meta-analysis conducted by Boardman et al.^16^ and Nudy et al.^17^. Subgroup analyses were performed when the number of studies was adequate. We evaluated the publication bias of included studies using symmetry funnel plots and Egger’s test. Statistical analysis was performed using the “meta” packages in the R version 3.4.1 software (R Foundation for Statistical Computing).

## Results

### Eligible studies and characteristics

**Figure 1:**
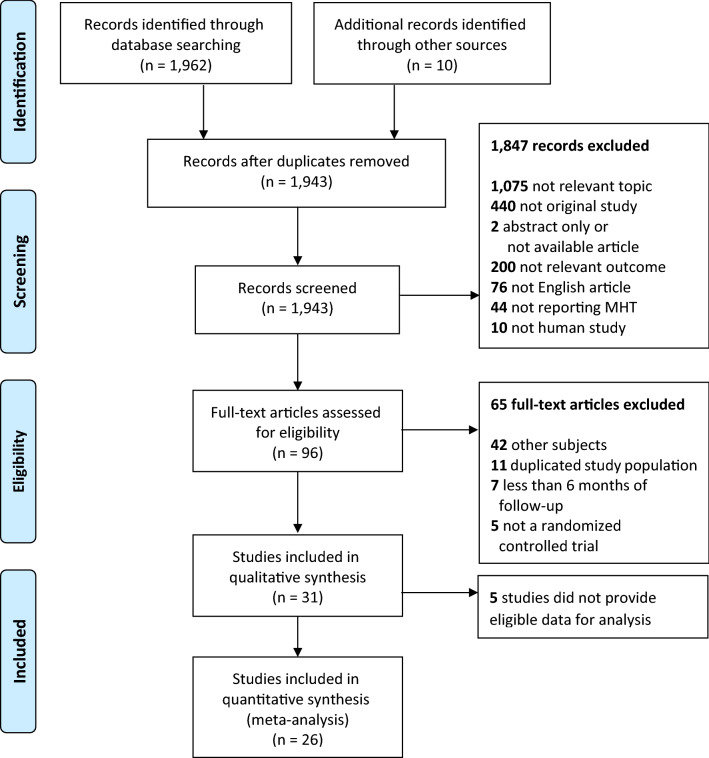
PRISMA flow diagram for study selection of the randomized controlled trials. From: Moher et al.^48^.

**Figure 2:**
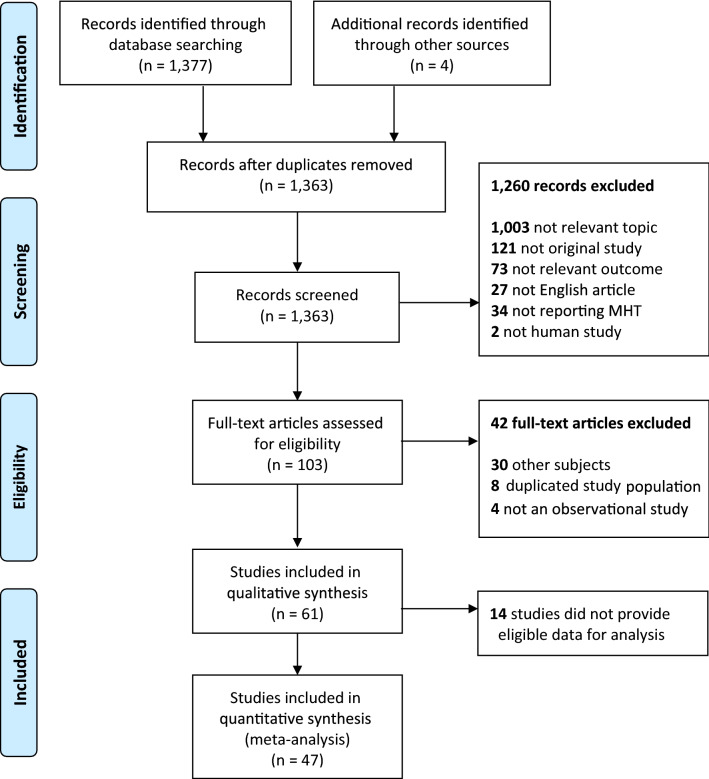
PRISMA flow diagram for study selection of the observational studies. From: Moher et al.^48^.

A total of 26 RCT studies (20 trials) and 47 observational studies were included in the final meta-analysis (Figs. 1, 2). We compared the characteristics of the included studies (Table 1). Most of the RCTs and observational studies were conducted in Europe or North America, and only a few studies were conducted in Asia. Eighteen RCTs (69.2%) were published before 2006, the year in which the WHI findings were being actively reevaluated. Twenty-nine of the observational studies (61.7%) were published after 2006. Study populations included in the RCTs were older than those included in the observational studies (median age, 63.6 vs. 60.6 years, respectively), and had more underlying diseases at baseline; subjects in the observational studies were relatively healthy. The route of MHT administration was oral in most RCTs, whereas some non-oral routes, such as transdermal or vaginal were used in the observational studies. The median follow-up duration of the RCTs was shorter than that of the observational studies (3.4 vs. 6.8 years). The observational studies included 30 cohort studies, 5 nested case–control studies, and 13 case–control studies.

**Table 1:** Overview of the characteristics of the included studies.

	Randomized controlled trials (n = 26 including 20 trials)	Observational studies (n = 47)
	N (%)	N (%)
**Ethnic group**		
Europe	7 (26.9)	29 (61.7)
North America	19 (73.1)	12 (25.5)
Rest of world	**–**	6 (12.8)
**Publication year**		
≤ 2006	18 (69.2)	18 (38.3)
** > **2006	8 (30.8)	29 (61.7)
**Age at baseline, years (median, range)**	63.6 (49.7–75.0)	60.6 (48.8–77.0)
Timing of initiation of MHT*^, †^		
Reported	22 (84.6)	36 (76.6)
Unreported	4 (15.4)	11 (23.4)
**Underlying diseases**		
With	17 (65.4)	6 (12.8)
Without	9 (34.6)	41 (87.2)
Regimen type*		
Reported	19 (73.1)	29 (617)
Unreported	7 (26.9)	18 (38.3)
Route of administration*		
Reported	25 (96.2)	21 (44.7)
Unreported	1 (3.8)	26 (55.3)
Duration of MHT*		
Reported	26 (100.0)	12 (25.5)
Unreported	–	35 (74.5)
Outcomes*		
All-cause death	16 (61.5)	15 (31.9)
Cardiovascular death	10 (38.5)	6 (12.8)
Stroke	12 ()	14 (29.8)
VTE	15 (57.7)	13 (27.7)
PE	7 (26.9)	4 (8.5)
MI	16 (61.5)	10 (21.3)
CHD	4 (15.4)	7 (14.9)
Angina	8 (30.8)	1 (2.1)
Revascularization	7 ()	0 (0.0)
**Follow-up, years (median, range)**	3.4 (0.7–18)	6.8 (1–21.5)
Study type*		
Cohort study	–	30 (63.8)
Case–control study	–	13 (27.7)
Nested case–control study	–	5 (10.6)

CHD, coronary heart disease; MHT, menopausal hormone therapy; MI, myocardial infarction; PE, pulmonary embolism; VTE, venous thromboembolism. *Individual studies often included more than one subgroup; †Women under 60 years of age or those in whom MHT was initiated within 10 years after menopause were included in the early subgroup; the others were included in the late subgroup.

### Quality assessment

Most of the RCTs were classified as good or fair quality studies according to the Jadad scale. Among the 20 trials, 15 were good quality and 5 were fair quality studies (Supplementary Table S7). The RCTs were assessed as fair quality studies for the following reasons: (1) incomplete blinding that affected the results or (2) allocation based on laboratory tests that could have increased selection bias.

Results of the quality assessment of cohort, nested case–control, and case–control studies by the NOS can be found in Supplementary Tables S8–S10. Among the 30 cohort studies, 25 and 5 studies were assessed as good/fair and poor quality studies, respectively. All 5 nested case–control studies were assessed as good quality. Studies with a cohort design were assessed as fair quality studies for the following reasons: (1) nurses or teachers included in the study population, increasing the risk of selection bias, or (2) MHT ascertained using a self-reported questionnaire. Studies with a cohort design were assessed as poor quality studies for the following reasons: (1) no control for confounding factors, such as age, disease history, and other lifestyle factors; (2) outcomes ascertained using a self-reported questionnaire; or (3) follow-up duration insufficient or follow-up rate not reported. Among the 13 case–control studies, 7 studies were assessed as good quality, and 6 studies were assessed as poor quality. Studies with a case–control design were assessed as poor quality studies for the following reasons: (1) no control for confounding factors, such as age, disease history, and other lifestyle factors; (2) MHT ascertained via interview and interviewer not blinded to case/control status; or (3) response rates differed between cases and controls.

### Meta-analysis of RCTs and observational studies

#### All-cause death and cardiovascular death

MHT was not associated with all-cause death (SE: 1.00, 95% CI: 0.96–1.04 in RCTs; SE: 0.90, 95% CI: 0.79–1.02 in observational studies) and cardiovascular death (SE: 0.96, 95% CI: 0.83–1.12 in RCTs; SE: 0.81, 95% CI: 0.61–1.07 in observational studies) in the pooled analysis of both RCTs and observational studies (Table 2). Subgroup analyses of RCTs did not identify any association between MHT and death (Table 3). In subgroup analyses of the observational studies, a decreased risk of all-cause death was observed among E only (SE: 0.85, 95% CI: 0.77–0.95) and early users after menopause (SE: 0.68, 95% CI: 0.51–0.92; Table 4).

**Table 2:** Meta-analysis of randomized controlled trials and observational studies for menopausal hormonal therapy (MHT) and cardiovascular disease (CVD) outcomes.

Outcomes	Randomized controlled trials				Observational studies			
	No. of trials	Summary estimates (95% CI)*	I^2^ (%)	P for heterogeneity	No. of studies	Summary estimates (95% CI)*	I^2^ (%)	P for heterogeneity
All-cause death	17	1.00 (0.96–1.04)	0.0	0.61	15	0.90 (0.79–1.02)	88.7	< 0.01
Cardiovascular death	11	0.96 (0.83–1.12)	39.5	0.09	6	0.81 (0.61–1.07)	32.9	0.19
Stroke	13	1.14 (1.04–1.25)	0.0	0.98	13	0.98 (0.85–1.13)	71.4	< 0.01
VTE	15	1.70 (1.33–2.16)	2.0	0.43	12	1.32 (1.13–1.54)	63.0	< 0.01
PE	8	1.26 (1.06–1.50)	20.7	0.27	4	1.44 (1.17–.1.76)	0.0	0.77
MI	17	1.04 (0.94–1.14)	0.0	0.51	10	0.79 (0.75–0.84)	0.0	0.89
CHD	5	1.02 (0.94–1.10)	0.0	0.43	7	0.91 (0.72–1.15)	75.4	< 0.01
Angina	8	0.95 (0.84–1.08)	14.3	0.32	1	1.11 (0.86–1.43)	–	–
Revascularization	7	0.96 (0.87–1.06)	14.1	0.32	0	–	–	–

CHD, coronary heart disease; CI, confidence interval; MI, myocardial infarction; PE, pulmonary embolism; VTE, venous thromboembolism. *Summary estimates (95% CI) were measured by fixed-effect models if I^2^ was < 30% and P for heterogeneity was > 0.05; otherwise, the summary estimates (95% CI) were measured by random-effect models.

**Table 3:** Subgroup analyses for menopausal hormonal therapy (MHT) and cardiovascular disease (CVD) outcomes in randomized controlled trials.

Variable	Subgroup	No. of trials	Summary estimates (95% CI)*	I^2^ (%)	P for heterogeneity
**All-cause death**					
Regimen	E only	5	0.96 (0.90–1.03)	0.0	0.42
	Combined EP	8	1.03 (0.97–1.08)	0.0	0.89
Duration	< 5 years	12	1.10 (0.94–1.29)	0.0	0.92
	≥ 5 years	5	0.99 (0.91–1.07)	43.2	0.13
Timing of initiation	Early	4	0.78 (0.57–1.07)	0.0	0.76
	Late	15	1.00 (0.96–1.05)	0.0	0.62
Underlying diseases	With	11	1.00 (0.96–1.04)	0.2	0.44
	Without	6	0.76 (0.51–1.15)	0.0	0.82
**Cardiovascular death**					
Regimen	E only	4	0.96 (0.85–1.07)	0.0	0.59
	Combined EP	4	1.04 (0.94–1.15)	0.0	0.64
Duration	< 5 years	7	0.83 (0.56–1.22)	0.0	0.47
	≥ 5 years	4	0.98 (0.83–1.16)	70.2	0.02
Timing of initiation	Early	1	0.26 (0.11–0.64)	–	–
	Late	10	1.01 (0.93–1.08)	0.0	0.55
Underlying diseases	With	10	1.01 (0.93–1.08)	0.0	0.55
	Without	1	0.26 (0.11–0.64)	–	–
**Stroke**					
Regimen	E only	4	1.15 (0.98–1.34)	0.0	0.86
	Combined EP	7	1.14 (1.01–1.29)	0.0	0.95
Duration	< 5 years	9	1.21 (0.91–1.63)	0.0	0.92
	≥ 5 years	4	1.13 (1.03–1.25)	0.0	0.84
Timing of initiation	Early	5	1.33 (0.91–1.93)	0.0	0.57
	Late	10	1.17 (1.01–1.37)	0.0	0.89
Underlying diseases	With	9	1.14 (1.04–1.26)	0.0	0.94
	Without	4	1.05 (0.63–1.78)	0.0	0.72
**VTE**					
Regimen	E only	4	1.33 (0.89–1.99)	0.0	0.55
	Combined EP	7	2.28 (1.64–3.18)	0.0	0.59
Duration	< 5 years	10	1.93 (1.10–3.36)	0.0	0.49
	≥ 5 years	5	1.65 (1.26–2.15)	27.7	0.24
Timing of initiation	Early	2	0.69 (0.25–1.93)	0.0	0.65
	Late	13	1.79 (1.39–2.29)	0.0	0.53
Underlying diseases	With	9	1.67 (1.29–2.17)	0.0	0.75
	Without	6	1.87 (0.71–4.94)	45.3	0.10
**PE**					
Regimen	E only	3	1.14 (0.88–1.49)	0.0	0.97
	Combined EP	3	2.09 (0.93–4.70)	64.8	0.06
Duration	< 5 years	3	1.89 (0.72–4.92)	23.8	0.27
	≥ 5 years	5	1.25 (1.05–1.48)	27.3	0.24
Timing of initiation	Early	3	1.73 (0.87–3.42)	14.0	0.31
	Late	7	1.88 (1.28–2.78)	0.0	0.49
Underlying diseases	With	5	1.24 (1.05–1.48)	0.0	0.47
	Without	3	2.08 (0.34–12.59)	50.4	0.13
**MI**					
Regimen	E only	4	1.02 (0.87–1.19)	0.0	0.97
	Combined EP	7	1.06 (0.94–1.20)	6.9	0.38
Duration	< 5 years	12	1.03 (0.69–1.55)	0.0	0.62
	≥ 5 years	5	1.02 (0.89–1.17)	34.4	0.19
Timing of initiation	Early	5	0.74 (0.50–1.11)	0.0	0.50
	Late	14	1.00 (0.86–1.17)	0.0	0.66
Underlying diseases	With	10	1.04 (0.94–1.14)	0.0	0.70
	Without	7	1.00 (0.43–2.29)	30.7	0.19
**CHD**					
Regimen	E only	2	0.93 (0.81–1.07)	0.0	0.83
	Combined EP	4	1.05 (0.96–1.15)	0.0	0.51
Duration	< 5 years	2	1.02 (0.80–1.30)	33.0	0.22
	≥ 5 years	3	1.01 (0.93–1.10)	13.0	0.32
Timing of initiation	Early	3	0.94 (0.66–1.33)	44.0	0.17
	Late	4	1.00 (0.87–1.14)	0.0	0.66
Underlying diseases	With	4	1.01 (0.93–1.09)	1.7	0.38
	Without	1	1.12 (0.90–1.40)	–	–
**Angina**					
Regimen	E only	2	0.96 (0.78–1.17)	0.0	0.69
	Combined EP	4	0.85 (0.71–1.01)	0.0	0.48
Duration	< 5 years	4	1.12 (0.76–1.63)	32.6	0.22
	≥ 5 years	4	0.91 (0.79–1.05)	0.0	0.57
Timing of initiation	Early	1	0.87 (0.54–1.41)	–	–
	Late	7	1.00 (0.86–1.17)	28.3	0.21
Underlying diseases	With	6	0.94 (0.83–1.07)	5.1	0.38
	Without	2	5.90 (0.71–49.13)	0.0	0.87
**Revascularization**					
Regimen	E only	3	0.91 (0.78–1.07)	0.0	0.75
	Combined EP	3	1.00 (0.87–1.15)	0.0	0.79
Duration	< 5 years	4	0.90 (0.54–1.49)	43.7	0.15
	≥ 5 years	3	0.98 (0.88–1.09)	0.0	0.73
Timing of initiation	Early	2	0.78 (0.54–1.13)	0.0	0.67
	Late	6	0.97 (0.86–1.10)	21.8	0.27
Underlying diseases	With	6	0.96 (0.87–1.07)	25.3	0.24
	Without	1	0.50 (0.05–5.43)	–	

CHD, coronary heart disease; CI, confidence interval; MI, myocardial infarction; E, estrogen; EP, estrogen and progesterone; PE, pulmonary embolism; VTE, venous thromboembolism. *Summary estimates (95% CI) were measured by fixed-effect models if the I^2^ was < 30% and P for heterogeneity was > 0.05; otherwise, the summary estimates (95% CI) were measured by random-effect models.

**Table 4:** Subgroup analyses for MHT and CVD outcomes in observational studies.

Variable	Subgroup	No. of studies	Summary estimates (95% CI)*	I^2^ (%)	P for heterogeneity
**All-cause death**					
Regimen	E only	7	0.85 (0.77–0.95)	59.5	0.02
	Combined EP	7	0.61 (0.34–1.09)	99.3	< 0.01
Duration	< 5 years	2	0.65 (0.25–1.64)	98.0	< 0.01
	≥ 5 years	2	0.81 (0.50–1.30)	88.5	< 0.01
Timing of initiation	Early	8	0.68 (0.51–0.92)	94.6	< 0.01
	Late	6	0.94 (0.73–1.21)	83.3	< 0.01
Routes of administration	Oral	2	1.01 (0.94–1.08)	0.0	0.75
	Non-oral	3	0.83 (0.65–1.07)	49.1	0.14
Underlying diseases	With	3	1.26 (0.34–4.64)	91.6	< 0.01
	Without	12	0.89 (0.78–1.01)	89.0	< 0.01
Recency of MHT	Past	4	0.95 (0.86–1.04)	57.8	0.07
	Current	4	0.90 (0.78–1.03)	85.8	< 0.01
Study design	Cohort	15	0.90 (0.79–1.02)	88.7	< 0.01
	Case–control	0	–	–	–
	Nested case–control	0	–	–	–
Study quality	Good and fair	12	0.89 (0.78–1.01)	89.0	< 0.01
	Poor	3	1.26 (0.34–4.64)	91.6	< 0.01
**Stroke**					
Regimen	E only	9	1.02 (0.90–1.16)	65.9	< 0.01
	Combined EP	6	1.05 (0.81–1.35)	92.0	< 0.01
Duration	< 5 years	3	1.11 (1.04–1.18)	0.0	0.43
	≥ 5 years	2	1.22 (1.16–1.29)	5.0	0.30
Timing of initiation	Early	4	0.81 (0.62–1.06)	22.0	0.28
	Late	6	0.91 (0.69–1.19)	59.2	0.03
Routes of administration	Oral	5	1.24 (1.11–1.39)	50.7	0.09
	Non-oral	5	0.86 (0.77–0.96)	0.0	0.91
Underlying diseases	With	3	1.19 (0.27–5.26)	81.8	< 0.01
	Without	10	1.00 (0.88–1.14)	69.6	< 0.01
Recency of MHT	Past	3	1.03 (0.99–1.07)	0.0	0.99
	Current	3	1.17 (1.12–1.22)	0.0	0.88
Study design	Cohort	9	0.97 (0.82–1.15)	55.1	0.02
	Case–control	2	0.84 (0.75–0.94)	0.0	0.44
	Nested case–control	2	1.22 (1.11–1.34)	0.0	0.82
Study quality	Good and fair	10	0.99 (0.87–1.14)	72.1	< 0.01
	Poor	3	1.27 (0.40–4.02)	77.3	0.01
**VTE**					
Regimen	E only	8	0.93 (0.79–1.08)	0.0	0.51
	Combined EP	6	2.21 (1.51–3.22)	90.1	< 0.01
Duration	< 5 years	4	1.23 (1.02–1.47)	0.0	0.88
	≥ 5 years	2	1.19 (0.95–1.51)	0.0	0.39
Timing of initiation	Early	6	1.55 (1.26–1.92)	31.4	0.20
	Late	5	1.27 (0.87–1.86)	2.9	< 0.01
Routes of administration	Oral	9	1.41 (1.19–1.67)	72.5	< 0.01
	Non-oral	7	0.81 (0.60–1.09)	70.8	< 0.01
Underlying diseases	With	0	–	–	–
	Without	12	1.32 (1.13–1.54)	63.0	< 0.01
Recency of MHT	Past	6	1.07 (0.97–1.19)	31.4	0.20
	Current	6	1.52 (1.45–1.60)	0.0	0.66
Study design	Cohort	6	1.25 (1.01–1.55)	38.9	0.15
	Case–control	5	1.43 (1.07–1.91)	80.8	< 0.01
	Nested case–control	1	1.34 (1.03–1.73)	–	–
Study quality	Good and fair	10	1.28 (1.08–1.51)	65.7	< 0.01
	Poor	2	1.60 (1.15–2.22)	0.0	0.35
**MI**					
Regimen	E only	9	0.85 (0.79–0.91)	0.0	0.67
	Combined EP	8	0.77 (0.71–0.84)	20.4	0.27
Duration	< 5 years	3	0.91 (0.73–1.12)	0.0	0.54
	≥ 5 years	2	0.51 (0.34–0.76)	0.2	0.32
Timing of initiation	Early	3	0.78 (0.62–0.98)	0.0	0.80
	Late	4	0.79 (0.73–0.84)	0.0	0.68
Routes of administration	Oral	2	0.87 (0.57–1.32)	83.3	0.01
	Non-oral	3	0.75 (0.60–0.93)	0.0	0.45
Underlying diseases	With	1	0.84 (0.72–0.98)	–	–
	Without	9	0.79 (0.74–0.84)	0.0	0.88
Recency of MHT	Past	4	0.84 (0.75–0.95)	0.0	0.68
	Current	4	0.81 (0.59–1.10)	76.7	< 0.01
Study design	Cohort	5	0.85 (0.76–0.95)	0.0	0.79
	Case–control	5	0.77 (0.72–0.83)	0.0	0.94
	Nested case–control	0	–	–	–
Study quality	Good and fair	7	0.78 (0.73–0.84)	0.0	0.78
	Poor	3	0.84 (0.74–0.95)	0.0	0.89

CI, confidence interval; MI, myocardial infarction; E, estrogen; EP, estrogen and progesterone; VTE, venous thromboembolism. *Summary estimates (95% CI) were measured by fixed-effect models if the I^2^ was < 30% and P for heterogeneity was > 0.05; otherwise, the summary estimates (95% CI) were measured by random-effect models.

#### Stroke

MHT was associated with an increased risk of stroke in the pooled analysis of RCTs (SE: 1.14, 95% CI: 1.04–1.25), although this was not observed in the pooled analysis of observational studies (SE: 0.98, 95% CI:0.85–1.13; Table 2). In the subgroup analyses of RCTs, an increased risk of stroke was observed in combined EP users (SE: 1.14, 95% CI: 1.01–1.29), users with a MHT duration ≥ 5 years (SE: 1.13, 95% CI: 1.03–1.25), late users after menopause (SE: 1.17, 95% CI: 1.01–1.37), and in women with an underlying disease at baseline (SE: 1.14, 95% CI: 1.04–1.26; Table 3). In subgroup analysis of the observational studies, an increased risk of stroke was observed in women administered oral MHT (SE: 1.24, 95% CI: 1.11–1.39), whereas a decreased risk of stroke was observed in women administered non-oral MHT (SE: 0.86, 95% CI: 0.77–0.96). There was no difference in risk by duration of MHT (SE: 1.11, 95% CI: 1.04–1.18 for < 5 years duration; SE: 1.22, 95% CI: 1.16–1.29 for ≥ 5 years duration; Table 4).

#### Venous thromboembolism

MHT was associated with an increased risk of VTE in the pooled results of both RCTs (SE: 1.70, 95% CI: 1.33–2.16) and observational studies (SE: 1.32, 95% CI: 1.13–1.54; Table 2). An increased risk of VTE was observed in combined EP users in both RCTs (SE: 2.28, 95% CI: 1.64–3.18; Table 3) and observational studies (SE: 2.21, 95% CI: 1.51–3.22; Table 4). This increased risk was also observed in late users after menopause (SE: 1.79, 95% CI: 1.39–2.29) and women with an underlying disease in the RCTs (SE: 1.67, 95% CI: 1.29–2.17; Table 3). It was not possible to evaluate the effects of underlying diseases on risk estimates in the observational studies, because women included in the observational studies were relatively healthy. Unlike findings from RCTs, an increased risk of VTE was observed in early users after menopause (SE: 1.55, 95% CI: 1.26–1.92), and in women administered oral MHT (SE: 1.41, 95% CI: 1.19–1.67; Table 4). There was no difference in risk by duration of MHT (SE: 1.93, 95% CI: 1.10–3.36 for < 5 years duration; SE: 1.65, 95% CI: 1.26–2.15 for ≥ 5 years duration) in the RCTs, although an increased risk was observed in use of MHT for < 5 years in observational studies (SE: 1.23, 95% CI: 1.02–1.47; Tables 3, 4). Regardless of study quality, an increased risk of VTE was observed in the observational studies (SE: 1.28, 95% CI: 1.08–1.51 in good and fair quality, SE: 1.60, 95% CI: 1.15–2.22 in poor quality; Table 4).

#### Pulmonary embolism

MHT was associated with an increased risk of PE in the pooled results of both RCTs (SE: 1.26, 95% CI: 1.06–1.50) and observational studies (SE: 1.44, 95% CI: 1.17–1.76; Table 2). In the subgroup analyses of RCTs, an increased risk of PE was observed in users with a MHT duration ≥ 5 years (SE: 1.25, 95% CI: 1.05–1.48), late users (SE: 1.88, 95% CI: 1.28–2.78), and women with an underlying disease at baseline (SE: 1.24, 95% CI: 1.05–1.48; Table 3).

#### Myocardial infarction and other outcomes

MHT was not associated with MI in the pooled results of RCTs (SE: 1.04, 95% CI: 0.94–1.14), whereas a decreased risk of MI was observed in the pooled results of observational studies (SE: 0.79, 95% CI: 0.75–0.84; Table 2). Subgroup analyses of RCTs did not reveal any association between MHT and MI (Table 3), whereas that of observational studies revealed a decreased risk in users with a MHT duration ≥ 5 years (SE: 0.51, 95% CI: 0.34–0.76), and with a non-oral route of MHT administration (SE: 0.75, 95% CI: 0.60–0.93). A decreased risk of MI was observed regardless of regimen type, timing of initiation, underlying diseases, study design, and quality of observational studies (Table 4).

The pooled results from both RCTs and observational studies did not reveal any association between MHT and CHD (SE: 1.02, 95% CI: 0.94–1.10 in RCTs; SE: 0.91, 95% CI: 0.72–1.15 in observational studies; Table 2). In the pooled results of the RCTs, there was also no association between MHT and revascularization (SE: 0.96, 95% CI: 0.87–1.06), or angina (SE: 0.95, 95% CI: 0.84–1.08; Table 2).

The forest plots for all analyses can be found in Supplementary Figures S1–S4.

#### Publication bias

There was no evidence of publication bias for all-cause death, cardiovascular death, stroke, VTE, PE, MI, CHD, angina, or revascularization in the RCTs or the observational studies (Egger’s *P*-value > 0.05). The funnel plots and Egger’s *P*-values calculated for the assessment of publication bias are included in Supplementary Figures S1 and S2.

## Discussion

### Summary of findings

RCTs had a shorter follow-up duration than did observational studies, and the study populations in the RCTs were older, initiated MHT late after menopause, and had more underlying diseases than those in the observational studies. RCTs and observational studies both showed that MHT was associated with an increased risk of VTE and PE, although only the RCTs revealed an increased risk of stroke among those administered MHT. A decreased risk of MI by MHT was identified in the observational studies, but the RCTs did not show this association. Although still unexplained in the current literature, differential clinical effects according to regimen type, timing of initiation, underlying disease, and route of administration were identified in subgroup analyses.

### Comparison of findings with previous systematic reviews and a meta-analysis

Our meta-analysis of RCTs was based on the Cochrane review published in 2015^16^. Among the included RCTs, 13 trials overlapped with those included in the Cochrane review^12,22–33^. Four other trials (ESPRIT, HERS, WHI I, and WHI II)^13,14,34–36^ were included according to our inclusion criteria. Three trials (EMS, KEEPS, and PHASE)^37–39^ were newly identified in this study. Two other trials^40,41^ with a higher risk of bias than other studies, and one trial^27^ assessing recurrent VTE as the outcome, were excluded.

Consistent with the Cochrane review^16^, our pooled results from the RCTs showed an increased risk of stroke, VTE, and PE among MHT users. However, the effect size in the current study was decreased compared to those in the Cochrane review. We considered multivariable adjusted-estimates as a priority for the meta-analysis, thus potentially attenuating the effects of confounding factors. Nudy et al. conducted another RCT-based meta-analysis to assess the assumption of the timing hypothesis^17^. They reported that younger MHT users had a decreased risk of all-cause death and cardiac events (a composite of cardiac mortality and non-fatal MI), whereas the risk of a composite of stroke, transient ischemic attack (TIA), and systemic embolism increased as age increased. Unlike the Cochrane review^16^ and our meta-analysis, they integrated stroke, TIA, and systemic embolism as an outcome. Thus, it is not possible to compare the results with those from this study.

Two previous RCT-based meta-analyses^16,17^ did not assess the effects by confounding factors owing to insufficient information, but a more recent systematic review^18^ reported the effects of the timing of initiation, route of administration, duration, and dose on CVD risk. They reported that a low dose of oral MHT and transdermal MHT may have beneficial effects on CVD, including stroke and VTE. However, they did not report a synthesis of results, and most of the results were derived from observational studies. They reviewed 33 studies that included 6 RCTs and 27 observational studies; thus, it is difficult to compare the results between RCTs and observational studies. As another limitation, they reported that most of the included studies had a low or moderate evidence level based on quality assessment. We conducted subgroup analysis according to the quality of the observational studies. Although we investigated the effect of MHT on CVD through a meta-analysis in a manner similar to older more conventional studies, our study is comparable to the most recent review.

### Comparison of findings between RCTs and observational studies

Our pooled analysis of both RCTs and observational studies identified consistent findings with respect to thrombotic events, and inconsistent findings regarding stroke and MI. However, differential associations in the subgroup analyses were observed. In our meta-analysis, timing of initiation and underlying diseases at baseline were likely to affect CVD outcomes. Mostly, late users and women who had an underlying disease at baseline had an increased risk of CVD outcomes, whereas early users and relatively healthy women had a decreased risk in both RCTs and observational studies. We found that the route of MHT administration was a possible factor for differential associations with CVD outcomes. Oral MHT was related to an increased risk of thrombotic events and stroke, whereas transdermal and vaginal MHT were comparatively safer than oral MHT in a review of observational studies^18^; however, this information was not available for RCTs^16,17^. Our subgroup analyses of observational studies according to route of administration supported findings from the most recent review^18,42^. The recent Nurses’ Health Study and the WHI Observational Study also have suggested the safety of low-dose vaginal estrogen with respect to the risk of CVD and cancer^43,44^.

### Strengths and limitations

In the current study, we compared the characteristics of RCTs and observational studies, and identified possible reasons for inconsistent findings through various subgroup analyses. However, it is necessary to be cautious when interpreting our findings owing to some limitations. First, most of the study subjects were Europeans or North Americans. Thus, it was difficult to identify ethnic differences, for example, the prevalence of CVD, and age at natural menopause^45,46^. Second, some observational studies defined MHT through a self-reported questionnaire, and the included RCTs used different treatment regimens. Our subgroup analysis only considered E only and combined EP regimen types, and therefore, we were unable to assess the effect of more detailed regimens. Although the heterogeneity of the observational studies was higher than that of the RCTs, it was slightly attenuated in the subgroup analyses. Third, methodologic limitations for the control of confounding effects may remain. However, because we extracted multivariable adjusted-estimates for the associations as a priority, some of the confounders may have been controlled in this study. Although atrial fibrillation (AF) has been known to be strongly associated with thrombotic events, most of the included studies in our meta-analysis did not take this into account^47^. Therefore, the potential strong association between AF and thrombotic events may have contributed to the consistent findings of increased risk of thrombotic events in both RCTs and observational studies. Further studies are necessary to evaluate well-known risk factors, such as AF, to identify the association between MHT and risk of thrombotic events. Finally, the largest WHI trials may have contributed to the RCT findings. When we performed sensitivity analyses after excluding the WHI trials, the results did not change except for stroke, which suggested that the increased risk of stroke in the RCTs may be overestimated, or may reflect the characteristics of women who received MHT. Nevertheless, to the best of our knowledge, our study is the first meta-analysis of both RCTs and observational studies that takes into account as many factors as possible, unlike previous meta-analyses. The meta-analysis of observational studies can also be comparable to that of RCTs, as the observational studies often have longer follow-ups and are conducted in a more real-world setting. Although the included observational studies were rated ‘good’ or ‘fair’ in the quality assessment, healthy user bias may remain, and therefore, the results should be interpreted with caution.

## Conclusion

Our findings support the idea that the risks and benefits of MHT are likely to depend on the characteristics of the women who are treated. MHT is still not recommended for the prevention of chronic diseases; however, it may have beneficial effects with respect to CVD and mortality in postmenopausal women with severe menopausal symptoms, after sufficient consideration of underlying diseases and timing of treatment initiation. Moreover, the use of non-oral types of MHT for menopausal symptoms may be suggested for women at high risk of VTE and stroke than oral types. Further studies to investigate the influence of ethnicity or specific MHT types are required.

## Supplementary information

Supplementary Figures.

Supplementary Information.
