# Supplementary material for: A systematic review and meta-analysis of effects of menopausal hormone therapy on cardiovascular diseases
Source: Sci Rep. 2020 Nov 26;10:20631. doi: 10.1038/s41598-020-77534-9 (PMC7691511; doi:10.1038/s41598-020-77534-9)
Supplement: Supplementary file 1 — Supplementary Figures. [file 41598_2020_77534_MOESM1_ESM.pdf]

Supplementary Figures

A systematic review and meta-analysis of effects of menopausal hormone therapy on cardiovascular diseases

Ji-Eun Kim[1, 2], Jae-Hyuck Chang[3], Min-Ji Jeong[3], Jaesung Choi[1, 2], JooYong Park[1, 2], Chaewon Baek[3], Aesun Shin[4, 5, 6], Sang Min Park[1, 7], Daehee Kang[4, 5, 6, 8] and Ji-Yeob Choi[1, 2, 5, 9 *]

[1]Department of Biomedical Sciences, Seoul National University Graduate School, Seoul, Korea;

[2]BK21plus Biomedical Science Project, Seoul National University College of Medicine, Seoul, Korea

[3]Northeastern University Bouve College of Health Sciences School of Pharmacy, Boston, MA, USA;

[4]Department of Preventive Medicine, Seoul National University College of Medicine, Seoul, Korea;

[5]Cancer Research Institute, Seoul National University, Seoul, Korea;

[6]Department of Innovative Medical Science, Seoul National University College of Medicine, Seoul, Korea;

[7]Department of Family Medicine, Seoul National University College of Medicine, Seoul, Korea;

[8]Institute of Environmental Medicine, Seoul National University Medical Research Center, Seoul, Korea;

[9]Institute of Health Policy and Management, Seoul National University Medical Research Center, Seoul, Korea

Supplementary Figure S1.1. Pooled results of MHT and all-cause death in the RCTs: (a) forest plot and (b) funnel plot.

Supplementary Figure S1.2. Pooled results of MHT and cardiovascular death in the RCTs: (a) forest plot and (b) funnel plot.

Supplementary Figure S1.3. Pooled results of MHT and stroke in the RCTs: (a) forest plot and (b) funnel plot.

Supplementary Figure S1.4. Pooled results of MHT and VTE in the RCTs: (a) forest plot and (b) funnel plot.

Supplementary Figure S1.5. Pooled results of MHT and PE in the RCTs: (a) forest plot and (b) funnel plot.

Supplementary Figure S1.6. Pooled results of MHT and MI in the RCTs: (a) forest plot and (b) funnel plot.

Supplementary Figure S1.7. Pooled results of MHT and CHD in the RCTs: (a) forest plot and (b) funnel plot.

Supplementary Figure S1.8. Pooled results of MHT and angina in the RCTs: (a) forest plot and (b) funnel plot.

Supplementary Figure S1.9. Pooled results of MHT and revascularization in the RCTs: (a) forest plot and (b) funnel plot.

Supplementary Figure S2.1. Pooled results of MHT and all-cause death in the observational studies: (a) forest plot and (b) funnel plot.

Supplementary Figure S2.2. Pooled results of MHT and cardiovascular death in the observational studies: (a) forest plot and (b) funnel plot.

Supplementary Figure S2.3. Pooled results of MHT and stroke in the observational studies: (a) forest plot and (b) funnel plot.

Supplementary Figure S2.4. Pooled results of MHT and VTE in the observational studies: (a) forest plot and (b) funnel plot.

Supplementary Figure S2.5. Pooled results of MHT and PE in the observational studies: (a) forest plot and (b) funnel plot.

Supplementary Figure S2.6. Pooled results of MHT and MI in the observational studies: (a) forest plot and (b) funnel plot.

Supplementary Figure S2.7. Pooled results of MHT and CHD in the observational studies: (a) forest plot and (b) funnel plot.

Supplementary Figure S2.8. Pooled results of MHT and angina in the observational studies: (a) forest plot.

Supplementary Figure S3.1.1. MHT and all-cause death in RCTs: subgroup results by regimen type.

Supplementary Figure S3.1.2. MHT and all-cause death in RCTs: subgroup results by duration of use.

Supplementary Figure S3.1.3. MHT and all-cause death in RCTs: subgroup results by timing of initiation.

Supplementary Figure S3.1.4. MHT and all-cause death in RCTs: subgroup results by underlying disease.

Supplementary Figure S3.2.1. MHT and cardiovascular death in RCTs: subgroup results by regimen type.

Supplementary Figure S3.2.2. MHT and cardiovascular death in RCTs: subgroup results by duration of use.

Supplementary Figure S3.2.3. MHT and cardiovascular death in RCTs: subgroup results by timing of initiation.

Supplementary Figure S3.2.4. MHT and cardiovascular death in RCTs: subgroup results by underlying disease.

Supplementary Figure S3.3.1. MHT and stroke in RCTs: subgroup results by regimen type.

Supplementary Figure S3.3.2. MHT and stroke in RCTs: subgroup results by duration of use.

Supplementary Figure S3.3.3. MHT and stroke in RCTs: subgroup results by timing of initiation.

Supplementary Figure S3.3.4. MHT and stroke in RCTs: subgroup results by underlying disease.

Supplementary Figure S3.4.1. MHT and VTE in RCTs: subgroup results by regimen type.

Supplementary Figure S3.4.2. MHT and VTE in RCTs: subgroup results by duration of use.

Supplementary Figure S3.4.3. MHT and VTE in RCTs: subgroup results by timing of initiation.

Supplementary Figure S3.4.4. MHT and VTE in RCTs: subgroup results by underlying disease.

Supplementary Figure S3.5.1. MHT and PE in RCTs: subgroup results by regimen type.

Supplementary Figure S3.5.2. MHT and PE in RCTs: subgroup results by duration of use.

Supplementary Figure S3.5.3. MHT and PE in RCTs: subgroup results by timing of initiation.

Supplementary Figure S3.5.4. MHT and PE in RCTs: subgroup results by underlying disease.

Supplementary Figure S3.6.1. MHT and MI in RCTs: subgroup results by regimen type.

Supplementary Figure S3.6.2. MHT and MI in RCTs: subgroup results by duration of use.

Supplementary Figure S3.6.3. MHT and MI in RCTs: subgroup results by timing of initiation.

Supplementary Figure S3.6.4. MHT and MI in RCTs: subgroup results by underlying disease.

Supplementary Figure S3.7.1. MHT and CHD in RCTs: subgroup results by regimen type.

Supplementary Figure S3.7.2. MHT and CHD in RCTs: subgroup results by duration of use.

Supplementary Figure S3.7.3. MHT and CHD in RCTs: subgroup results by timing of initiation.

Supplementary Figure S3.7.4. MHT and CHD in RCTs: subgroup results by underlying disease.

Supplementary Figure S3.8.1. MHT and angina in RCTs: subgroup results by regimen type.

Supplementary Figure S3.8.2. MHT and angina in RCTs: subgroup results by duration of use.

Supplementary Figure S3.8.3. MHT and angina in RCTs: subgroup results by timing of initiation.

Supplementary Figure S3.8.4. MHT and angina in RCTs: subgroup results by underlying disease.

Supplementary Figure S3.9.1. MHT and revascularization in RCTs: subgroup results by regimen type.

Supplementary Figure S3.9.2. MHT and revascularization in RCTs: subgroup results by duration of use.

Supplementary Figure S3.9.3. MHT and revascularization in RCTs: subgroup results by timing of initiation.

Supplementary Figure S3.9.4. MHT and revascularization in RCTs: subgroup results by underlying disease.

Supplementary Figure S4.1.1. MHT and all-cause death in observational studies: subgroup results by regimen type.

Supplementary Figure S4.1.2. MHT and all-cause death in observational studies: subgroup results by duration of use.

Supplementary Figure S4.1.3. MHT and all-cause death in observational studies: subgroup results by timing of initiation.

Supplementary Figure S4.1.4. MHT and all-cause death in observational studies: subgroup results by route of administration.

Supplementary Figure S4.1.5. MHT and all-cause death in observational studies: subgroup results by underlying disease.

Supplementary Figure S4.1.6. MHT and all-cause death in observational studies: subgroup results by recency of MHT.

Supplementary Figure S4.1.7. MHT and all-cause death in observational studies: subgroup results by study design.

Supplementary Figure S4.1.8. MHT and all-cause death in observational studies: subgroup results by study quality.

Supplementary Figure S4.2.1. MHT and stroke in observational studies: subgroup results by regimen type.

Supplementary Figure S4.2.2. MHT and stroke in observational studies: subgroup results by duration of use.

Supplementary Figure S4.2.3. MHT and stroke in observational studies: subgroup results by timing of initiation.

Supplementary Figure S4.2.4. MHT and stroke in observational studies: subgroup results by route of administration.

Supplementary Figure S4.2.5. MHT and stroke in observational studies: subgroup results by underlying disease.

Supplementary Figure S4.2.6. MHT and stroke in observational studies: subgroup results by recency of MHT.

Supplementary Figure S4.2.7. MHT and stroke in observational studies: subgroup results by study design.

Supplementary Figure S4.2.8. MHT and stroke in observational studies: subgroup results by study quality.

Supplementary Figure S4.3.1. MHT and VTE in observational studies: subgroup results by regimen type.

Supplementary Figure S4.3.2. MHT and VTE in observational studies: subgroup results by duration of use.

Supplementary Figure S4.3.3. MHT and VTE in observational studies: subgroup results by timing of initiation.

Supplementary Figure S4.3.4. MHT and VTE in observational studies: subgroup results by route of administration.

Supplementary Figure S4.3.5. MHT and VTE in observational studies: subgroup results by underlying disease.

Supplementary Figure S4.3.6. MHT and VTE in observational studies: subgroup results by recency of MHT.

Supplementary Figure S4.3.7. MHT and VTE in observational studies: subgroup results by study design.

Supplementary Figure S4.3.8. MHT and VTE in observational studies: subgroup results by study quality.

Supplementary Figure S4.4.1. MHT and MI in observational studies: subgroup results by regimen type.

Supplementary Figure S4.4.2. MHT and MI in observational studies: subgroup results by duration of use.

Supplementary Figure S4.4.3. MHT and MI in observational studies: subgroup results by timing of initiation.

Supplementary Figure S4.4.4. MHT and MI in observational studies: subgroup results by route of administration.

Supplementary Figure S4.4.5. MHT and MI in observational studies: subgroup results by underlying disease.

Supplementary Figure S4.4.6. MHT and MI in observational studies: subgroup results by recency of MHT.

Supplementary Figure S4.4.7. MHT and MI in observational studies: subgroup results by study design.

Supplementary Figure S4.4.8. MHT and MI in observational studies: subgroup results by study quality.

Supplementary Figure S1.1. Pooled results of MHT and all-cause death in the RCTs: (a) forest plot and (b) funnel plot.

(a)

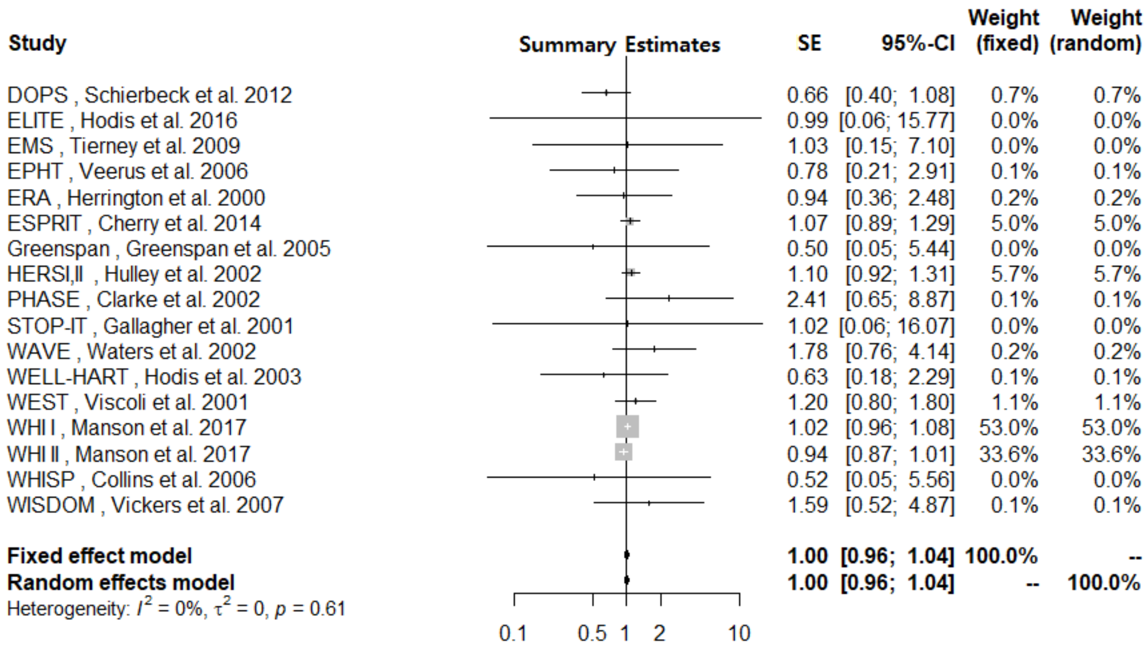

(b)

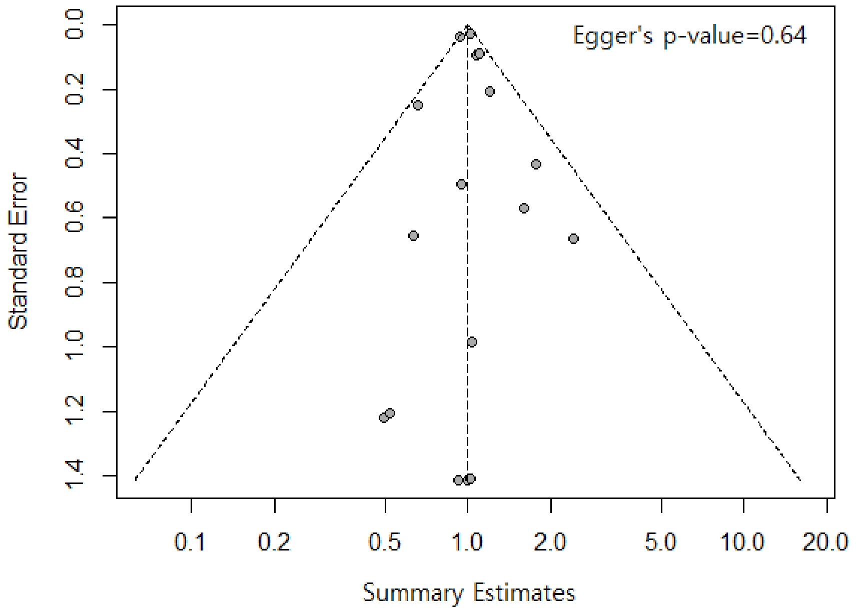

Summary estimates (95% CI) were measured by fixed-effect models if I^2 was <30% and P for heterogeneity was >0.05; otherwise, the summary estimates (95% CI) were measured by random-effect models. Forest and funnel plots were generated by "meta" package in R version 3.4.1. MHT, menopausal hormone therapy; RCTs, randomized controlled trials; SE, summary estimates.

Supplementary Figure S1.2. Pooled results of MHT and cardiovascular death in the RCTs: (a) forest plot and (b) funnel plot.

(a)

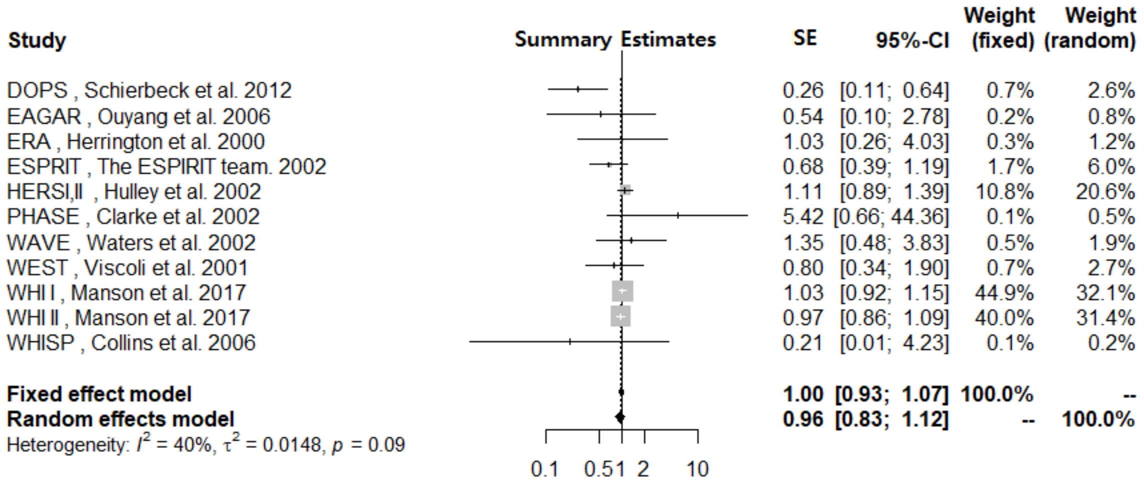

(b)

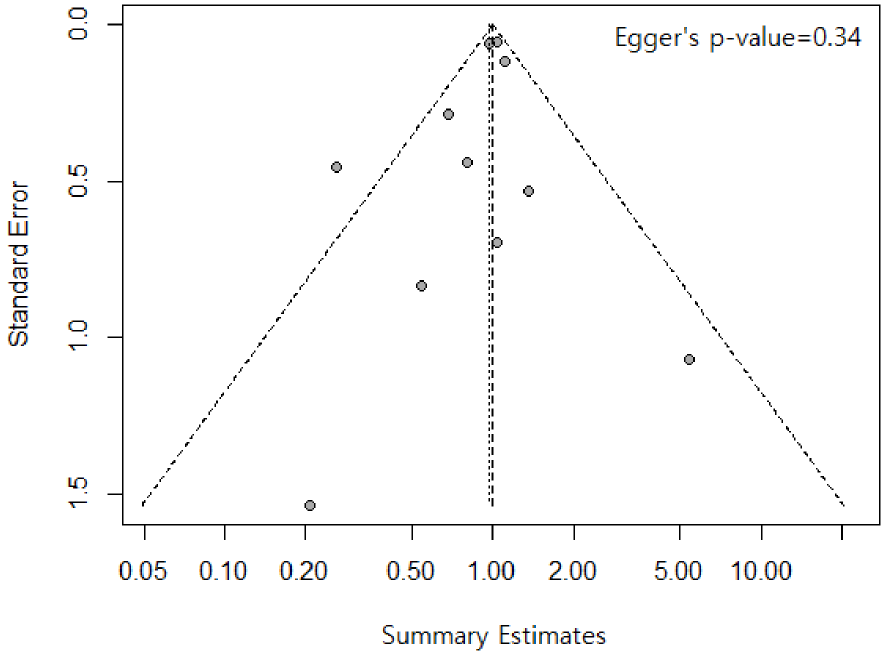

Summary estimates (95% CI) were measured by fixed-effect models if I^2 was <30% and P for heterogeneity was >0.05; otherwise, the summary estimates (95% CI) were measured by random-effect models. Forest and funnel plots were generated by "meta" package in R version 3.4.1. MHT, menopausal hormone therapy; RCTs, randomized controlled trials; SE, summary estimates.

Supplementary Figure S1.3. Pooled results of MHT and stroke in the RCTs: (a) forest plot and (b) funnel plot.

(a)

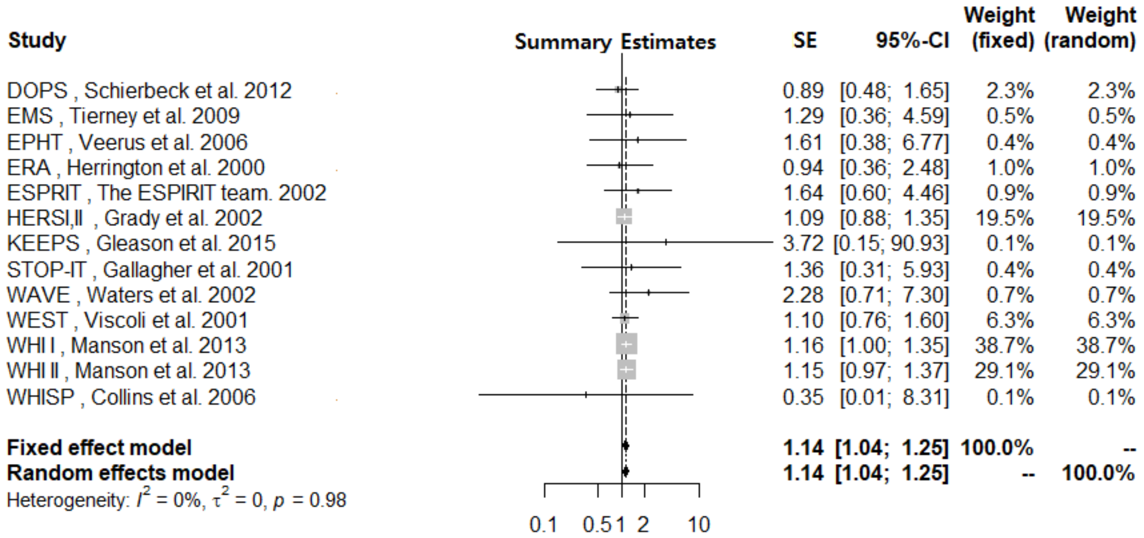

(b)

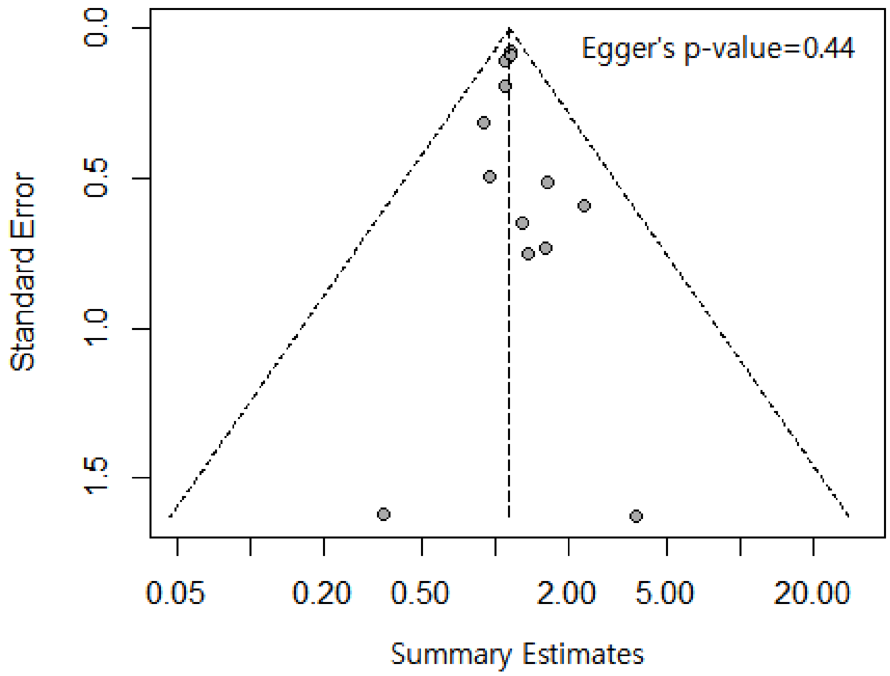

Summary estimates (95% CI) were measured by fixed-effect models if I^2 was <30% and P for heterogeneity was >0.05; otherwise, the summary estimates (95% CI) were measured by random-effect models. Forest and funnel plots were generated by "meta" package in R version 3.4.1. MHT, menopausal hormone therapy; RCTs, randomized controlled trials; SE, summary estimates.

Supplementary Figure S1.4. Pooled results of MHT and VTE in the RCTs: (a) forest plot and (b) funnel plot.

(a)

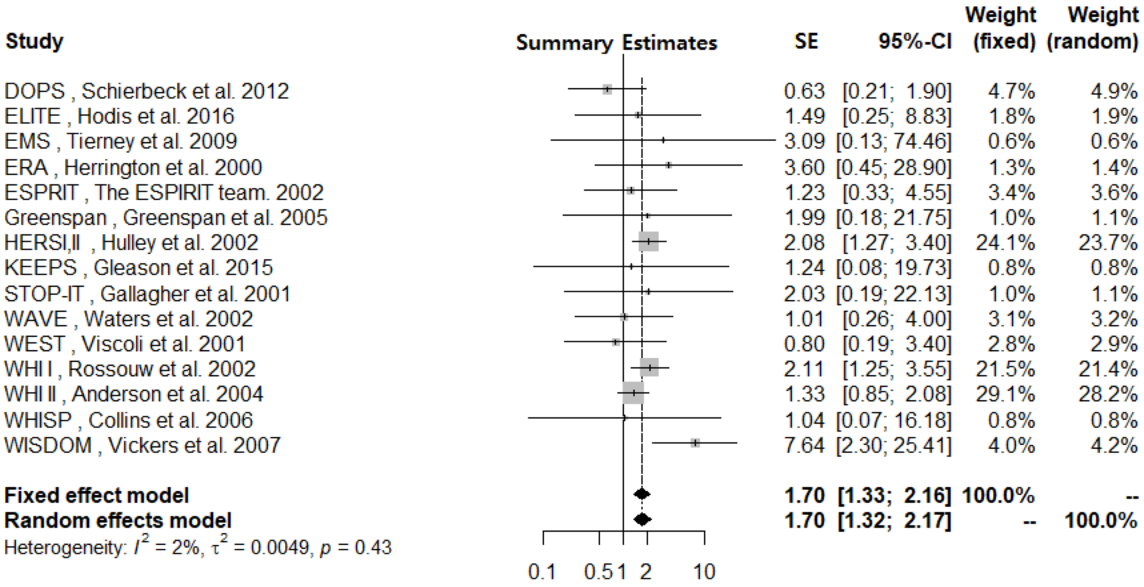

(b)

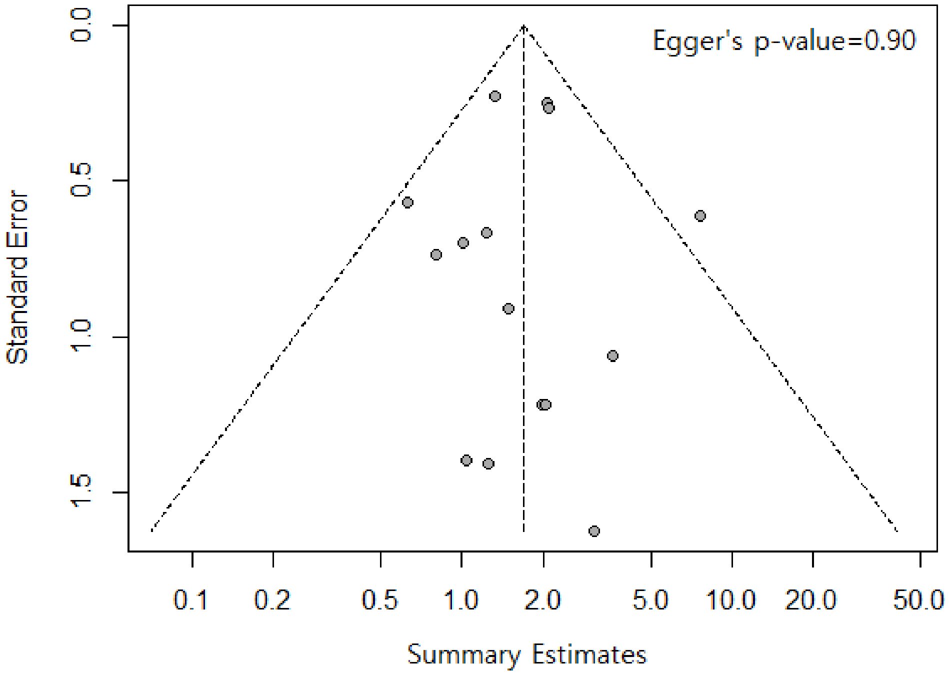

Summary estimates (95% CI) were measured by fixed-effect models if I^2 was <30% and P for heterogeneity was >0.05; otherwise, the summary estimates (95% CI) were measured by random-effect models. Forest and funnel plots were generated by "meta" package in R version 3.4.1. MHT, menopausal hormone therapy; RCTs, randomized controlled trials; SE, summary estimates; VTE, venous thromboembolism.

Supplementary Figure S1.5. Pooled results of MHT and PE in the RCTs: (a) forest plot and (b) funnel plot.

(a)

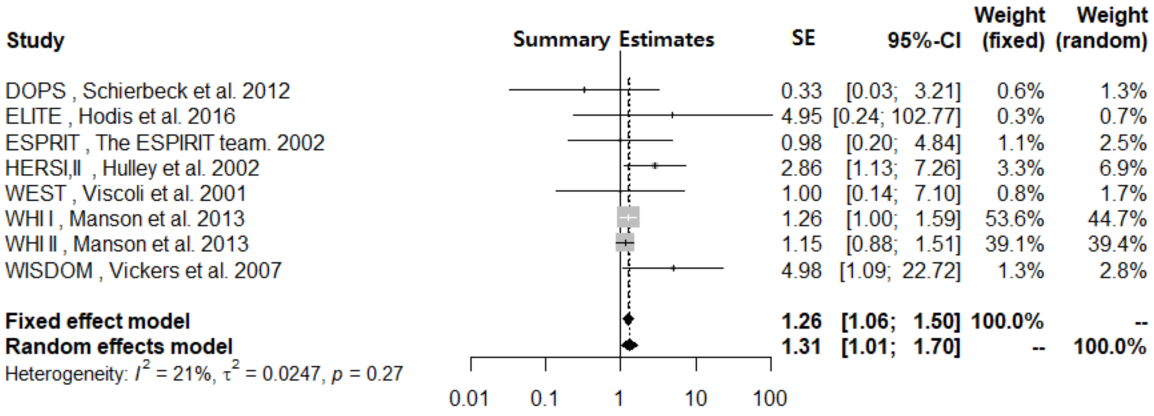

(b)

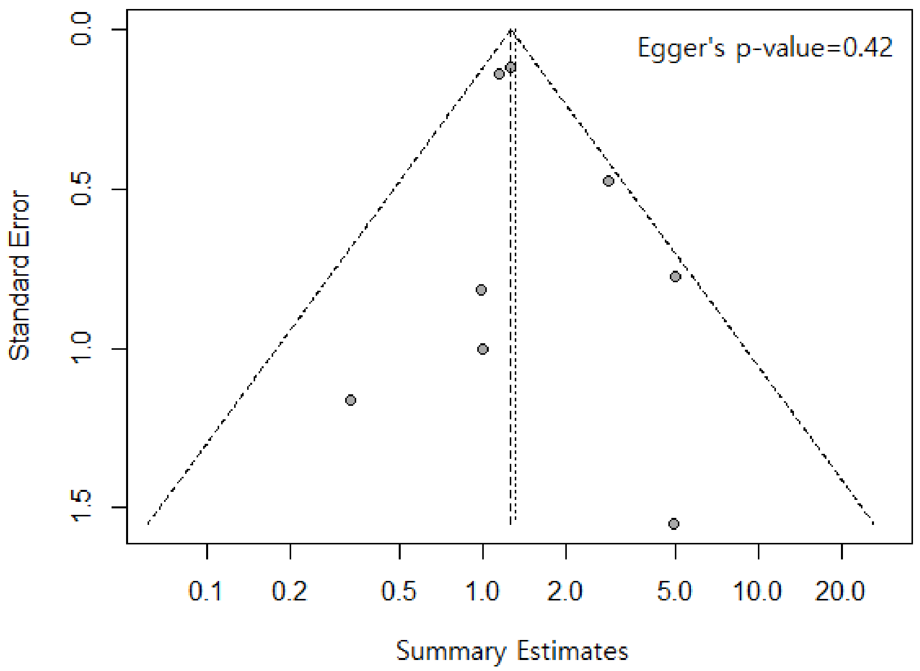

Summary estimates (95% CI) were measured by fixed-effect models if I^2 was <30% and P for heterogeneity was >0.05; otherwise, the summary estimates (95% CI) were measured by random-effect models. Forest and funnel plots were generated by "meta" package in R version 3.4.1. MHT, menopausal hormone therapy; PE, pulmonary embolism; RCTs, randomized controlled trials; SE, summary estimates.

Supplementary Figure S1.6. Pooled results of MHT and MI in the RCTs: (a) forest plot and (b) funnel plot.

(a)

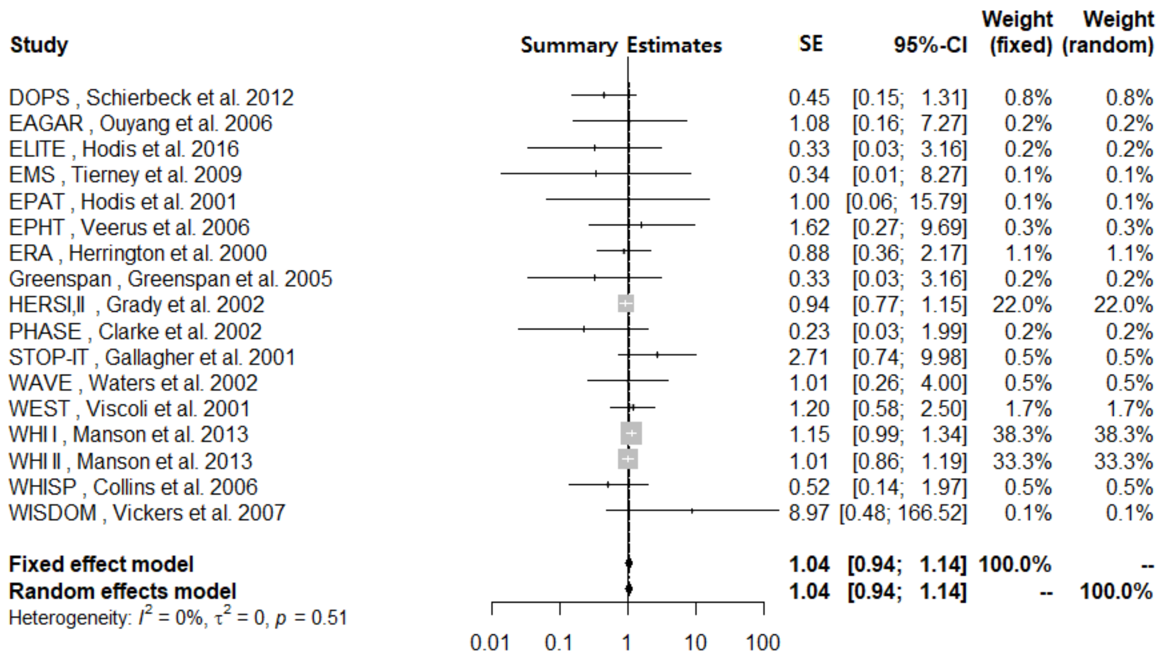

(b)

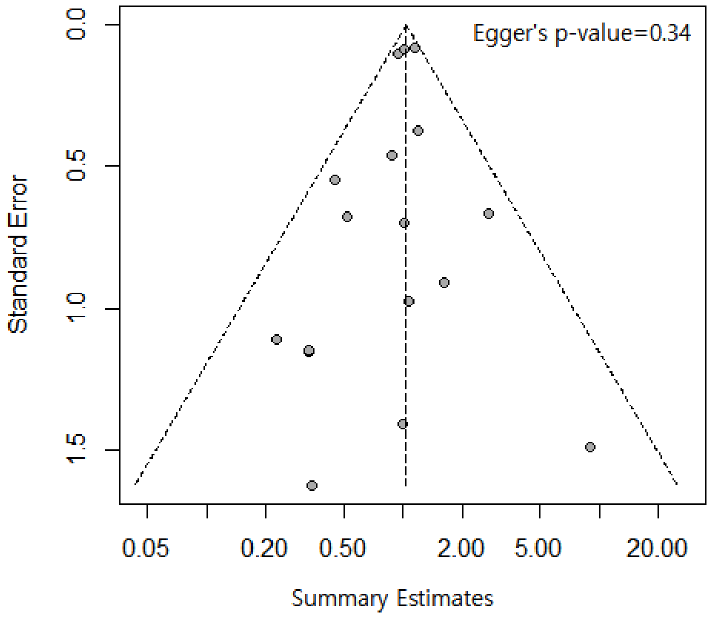

Summary estimates (95% CI) were measured by fixed-effect models if I^2 was <30% and P for heterogeneity was >0.05; otherwise, the summary estimates (95% CI) were measured by random-effect models. Forest and funnel plots were generated by "meta" package in R version 3.4.1. MHT, menopausal hormone therapy; MI, myocardial infarction; RCTs, randomized controlled trials; SE, summary estimates.

Supplementary Figure S1.7. Pooled results of MHT and CHD in the RCTs: (a) forest plot and (b) funnel plot.

(a)

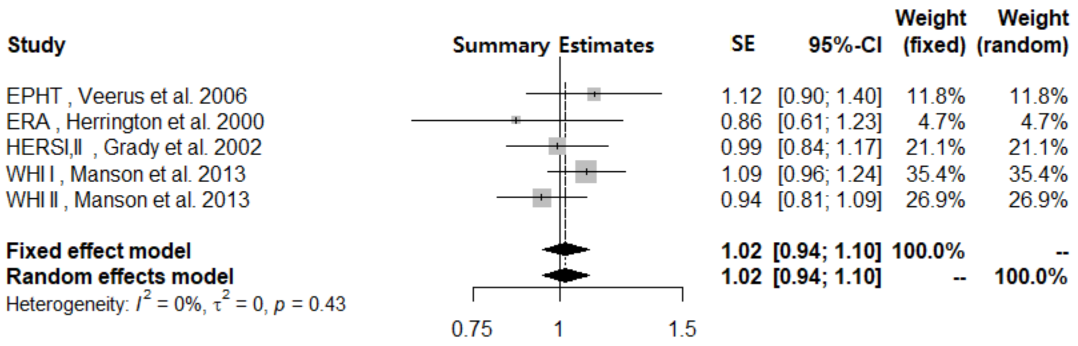

(b)

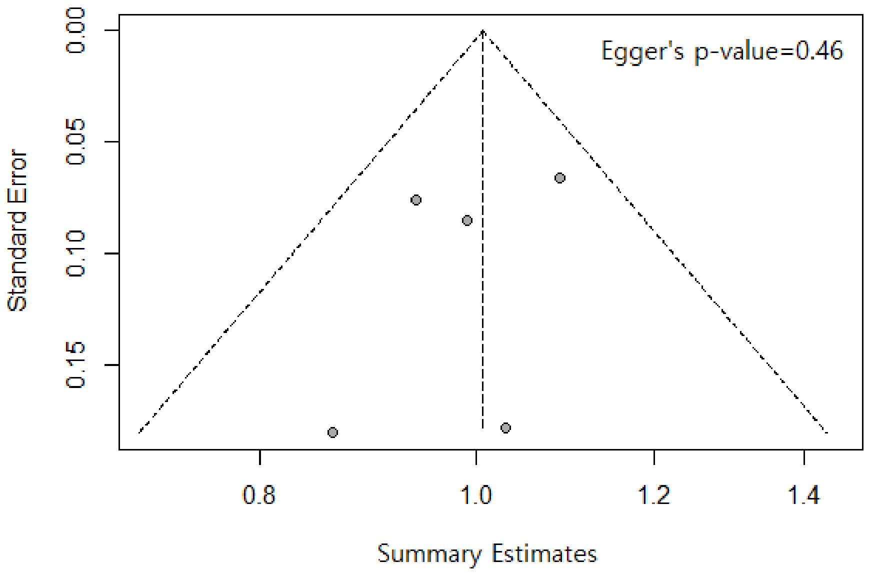

Summary estimates (95% CI) were measured by fixed-effect models if I^2 was <30% and P for heterogeneity was >0.05; otherwise, the summary estimates (95% CI) were measured by random-effect models. Forest and funnel plots were generated by "meta" package in R version 3.4.1. CHD, coronary heart disease; MHT, menopausal hormone therapy; RCTs, randomized controlled trials; SE, summary estimates.

Supplementary Figure S1.8. Pooled results of MHT and angina in the RCTs: (a) forest plot and (b) funnel plot.

(a)

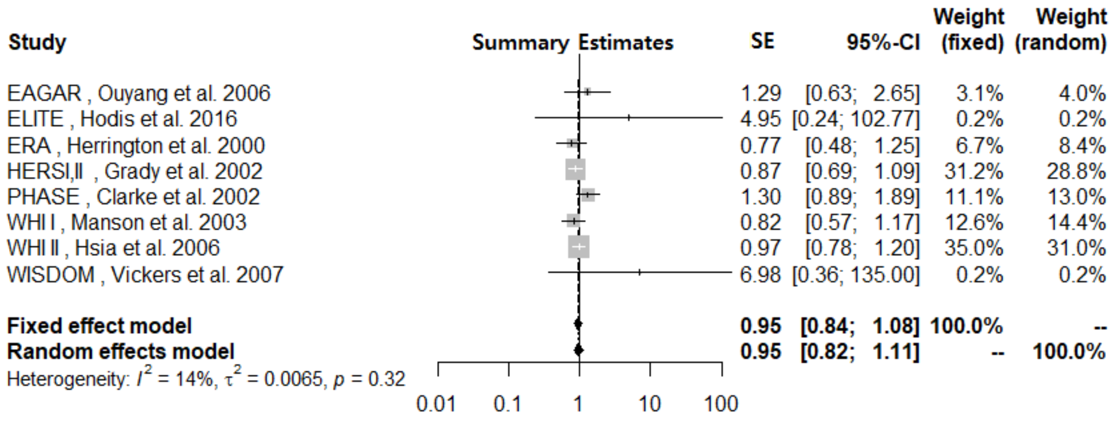

(b)

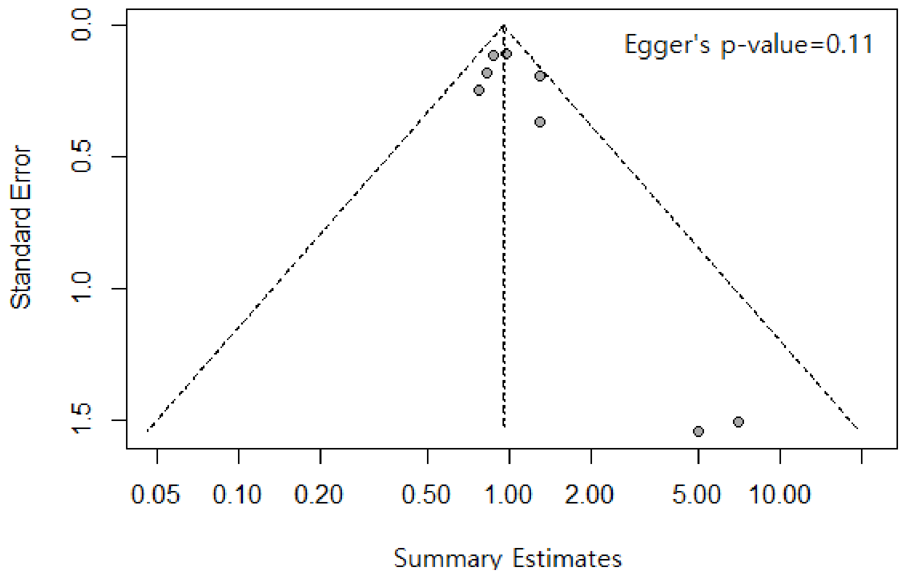

Summary estimates (95% CI) were measured by fixed-effect models if I^2 was <30% and P for heterogeneity was >0.05; otherwise, the summary estimates (95% CI) were measured by random-effect models. Forest and funnel plots were generated by "meta" package in R version 3.4.1. MHT, menopausal hormone therapy; RCTs, randomized controlled trials; SE, summary estimates.

Supplementary Figure S1.9. Pooled results of MHT and revascularization in the RCTs: (a) forest plot and (b) funnel plot.

(a)

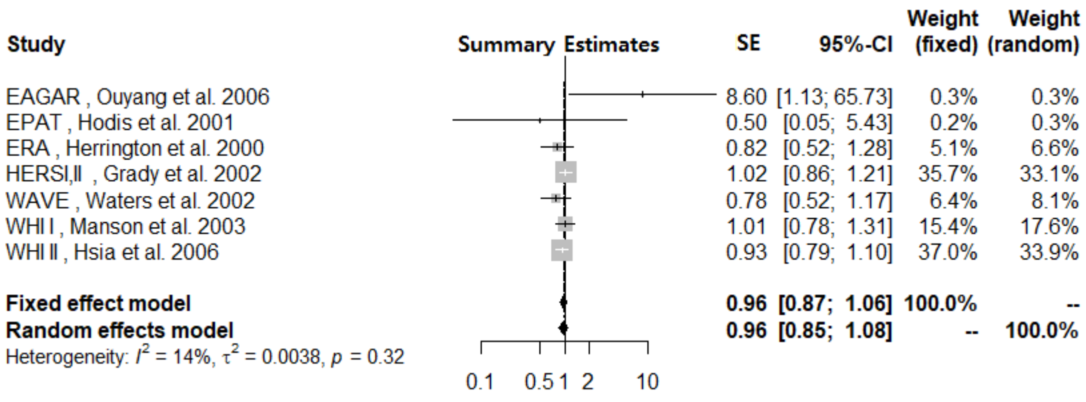

(b)

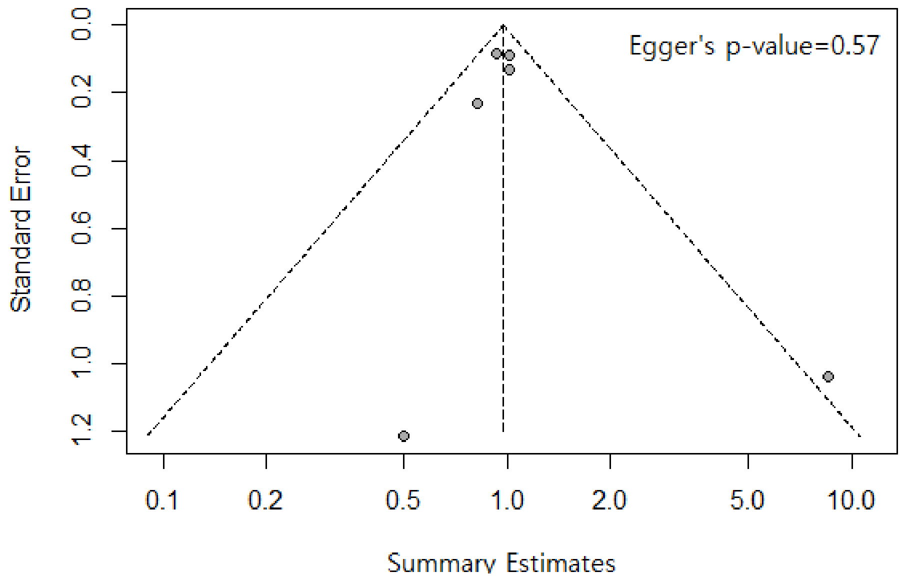

Summary estimates (95% CI) were measured by fixed-effect models if I^2 was <30% and P for heterogeneity was >0.05; otherwise, the summary estimates (95% CI) were measured by random-effect models. Forest and funnel plots were generated by "meta" package in R version 3.4.1. MHT, menopausal hormone therapy; RCTs, randomized controlled trials; SE, summary estimates.

Supplementary Figure S2.1. Pooled results of MHT and all-cause death in the observational studies: (a) forest plot and (b) funnel plot.

(a)

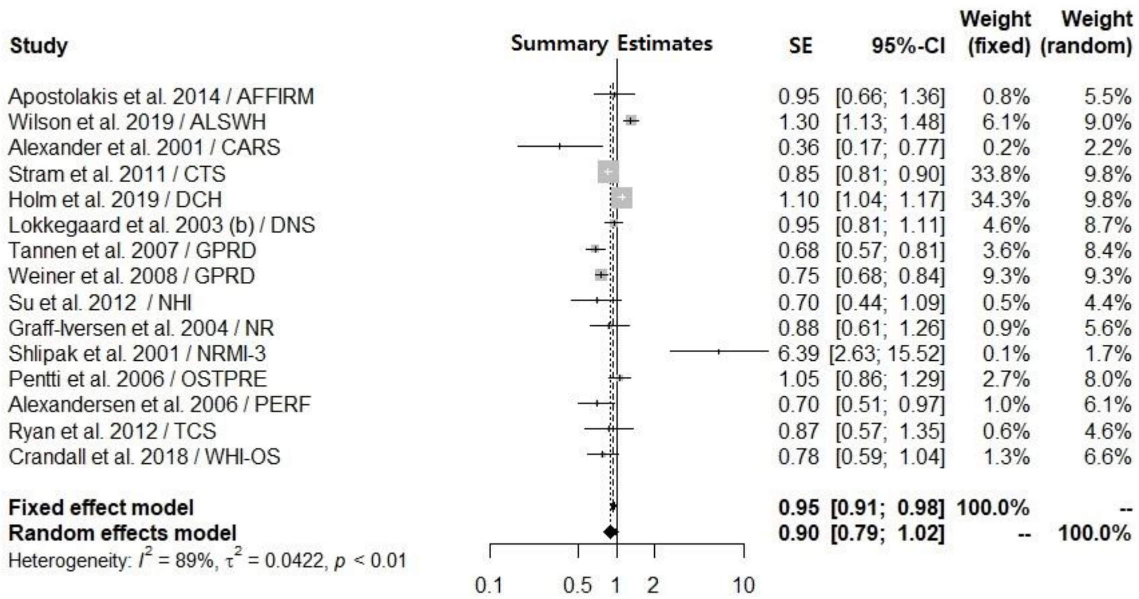

(b)

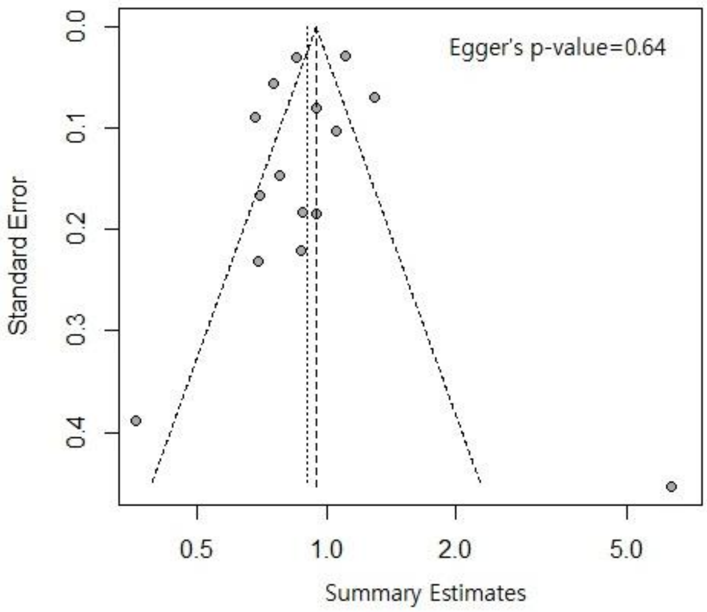

Summary estimates (95% CI) were measured by fixed-effect models if I^2 was <30% and P for heterogeneity was >0.05; otherwise, the summary estimates (95% CI) were measured by random-effect models. Forest and funnel plots were generated by "meta" package in R version 3.4.1. MHT, menopausal hormone therapy; SE, summary estimates.

Supplementary Figure S2.2. Pooled results of MHT and cardiovascular death in the observational studies: (a) forest plot and (b) funnel plot.

(a)

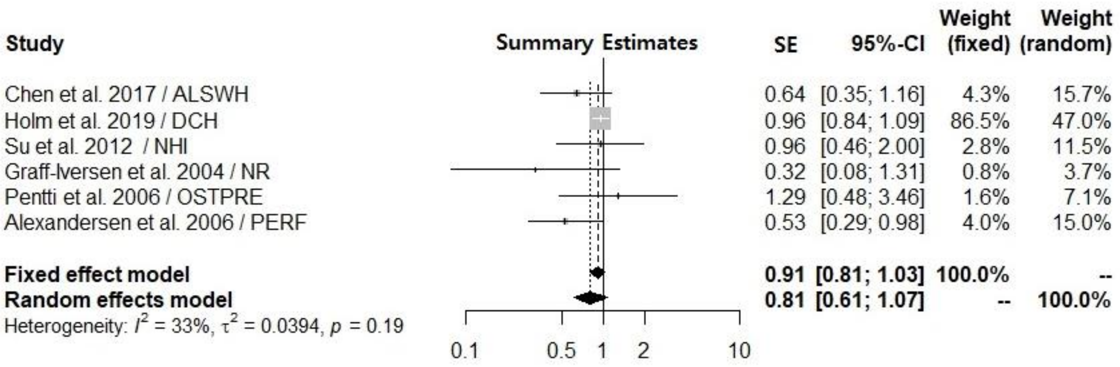

(b)

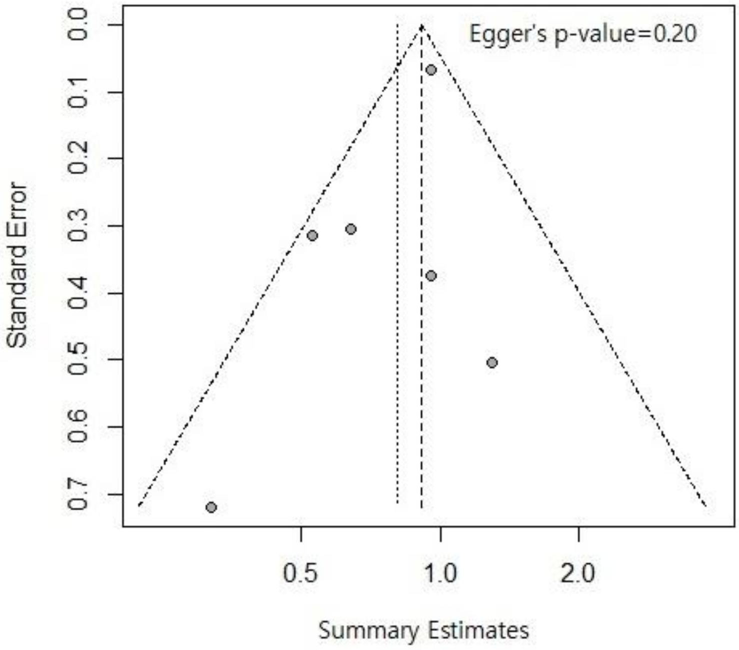

Summary estimates (95% CI) were measured by fixed-effect models if I^2 was <30% and P for heterogeneity was >0.05; otherwise, the summary estimates (95% CI) were measured by random-effect models. Forest and funnel plots were generated by "meta" package in R version 3.4.1. MHT, menopausal hormone therapy; SE, summary estimates.

Supplementary Figure S2.3. Pooled results of MHT and stroke in the observational studies: (a) forest plot and (b) funnel plot.

(a)

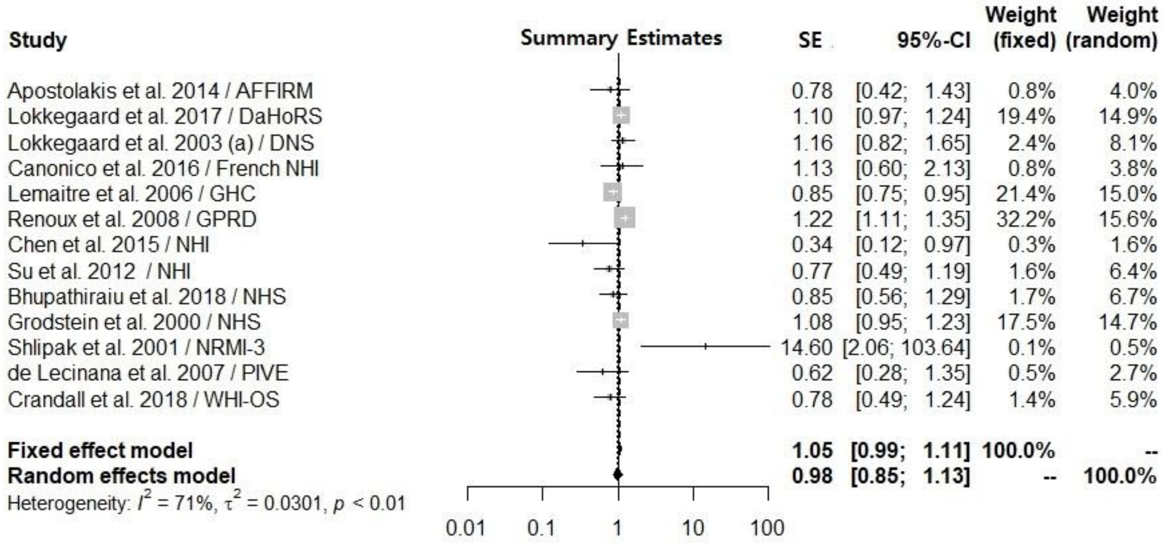

(b)

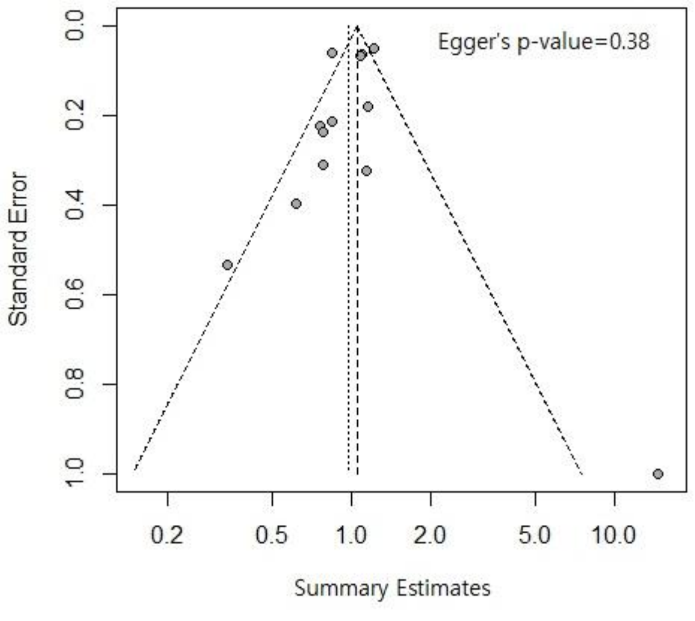

Summary estimates (95% CI) were measured by fixed-effect models if I^2 was $<30\%$ and P for heterogeneity was >0.05; otherwise, the summary estimates (95% CI) were measured by random-effect models. Forest and funnel plots were generated by "meta" package in R version 3.4.1. MHT, menopausal hormone therapy; SE, summary estimates.

Supplementary Figure S2.4. Pooled results of MHT and VTE in the observational studies: (a) forest plot and (b) funnel plot.

(a)

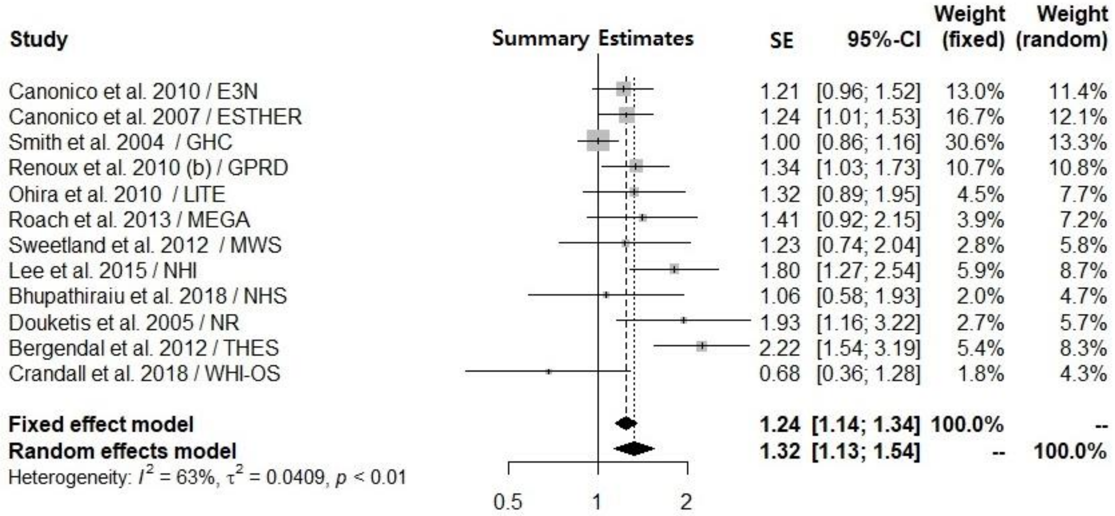

(b)

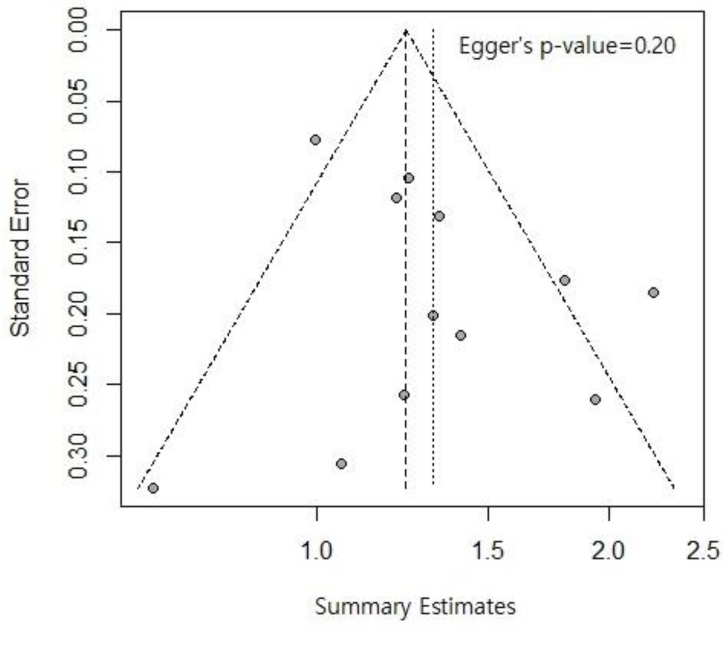

Summary estimates (95% CI) were measured by fixed-effect models if I^2 was <30% and P for heterogeneity was >0.05; otherwise, the summary estimates (95% CI) were measured by random-effect models. Forest and funnel plots were generated by "meta" package in R version 3.4.1. MHT, menopausal hormone therapy; SE, summary estimates; VTE, venous thromboembolism.

Supplementary Figure S2.5. Pooled results of MHT and PE in the observational studies: (a) forest plot and (b) funnel plot.

(a)

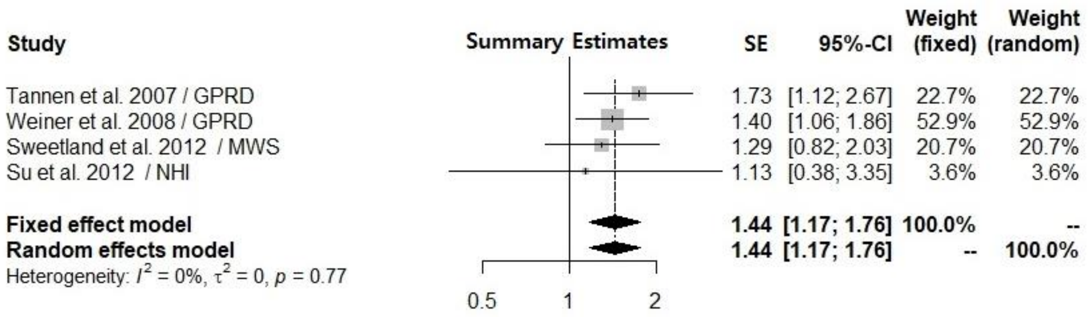

(b)

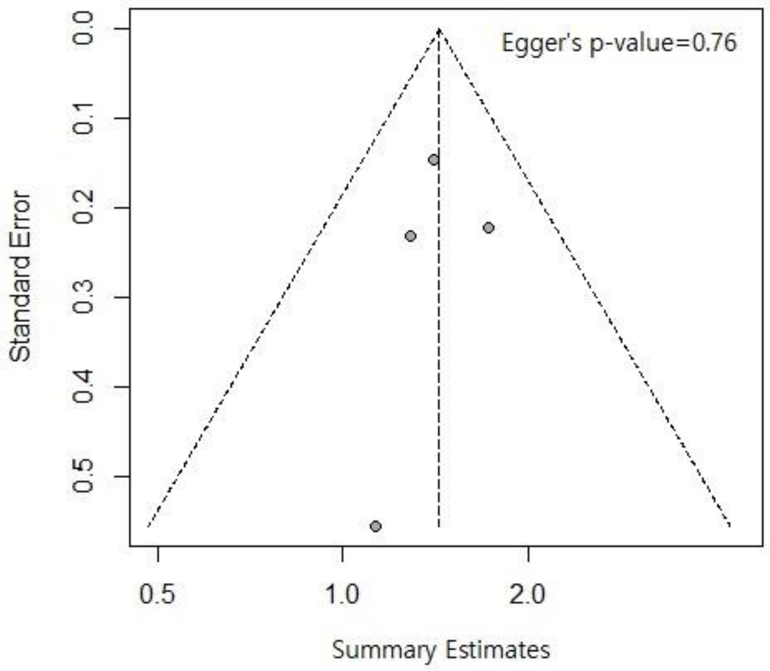

Summary estimates (95% CI) were measured by fixed-effect models if I^2 was <30% and P for heterogeneity was >0.05; otherwise, the summary estimates (95% CI) were measured by random-effect models. Forest and funnel plots were generated by "meta" package in R version 3.4.1. MHT, menopausal hormone therapy; PE, pulmonary embolism; SE, summary estimates.

Supplementary Figure S2.6. Pooled results of MHT and MI in the observational studies: (a) forest plot and (b) funnel plot.

(a)

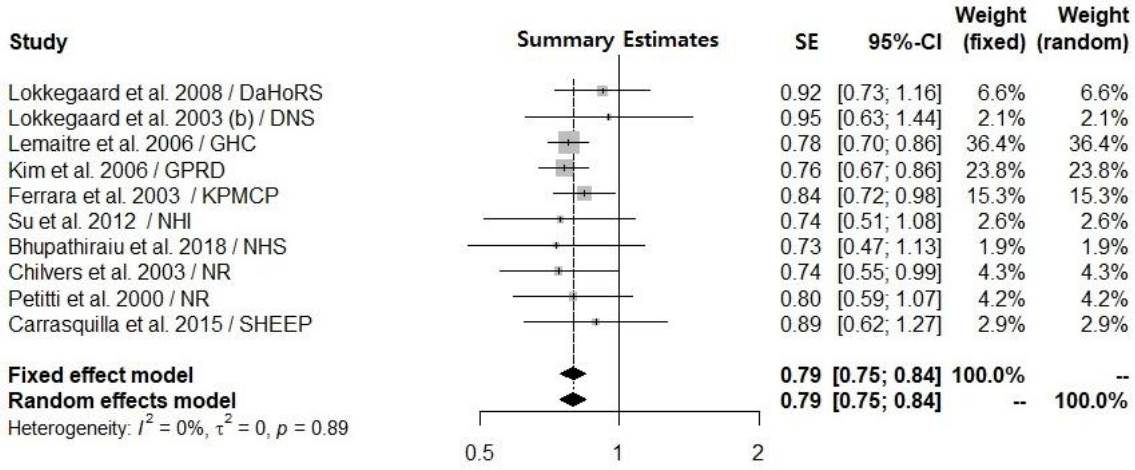

(b)

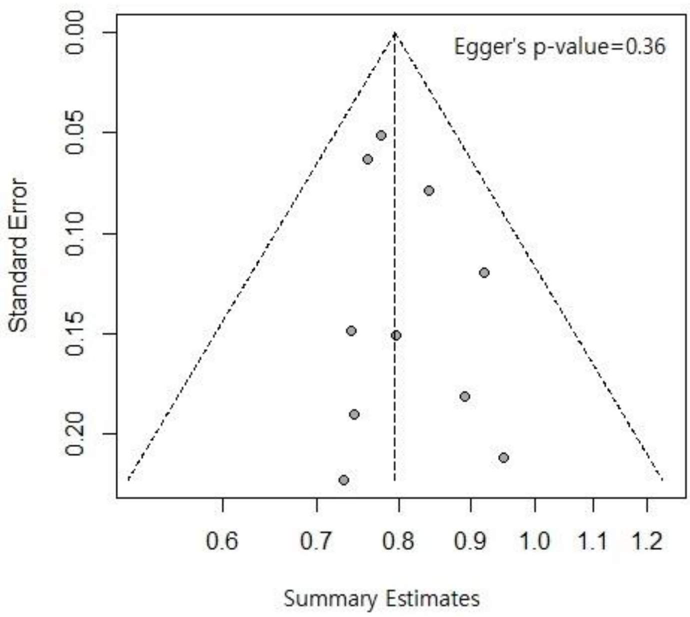

Summary estimates (95% CI) were measured by fixed-effect models if I^2 was <30% and P for heterogeneity was >0.05; otherwise, the summary estimates (95% CI) were measured by random-effect models. Forest and funnel plots were generated by "meta" package in R version 3.4.1. MHT, menopausal hormone therapy; MI, myocardial infarction; SE, summary estimates.

Supplementary Figure S2.7. Pooled results of MHT and CHD in the observational studies: (a) forest plot and (b) funnel plot.

(a)

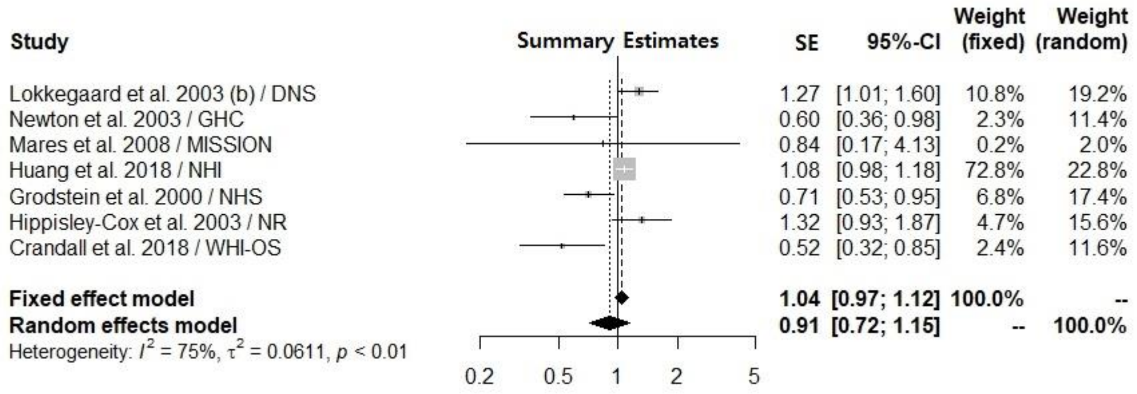

(b)

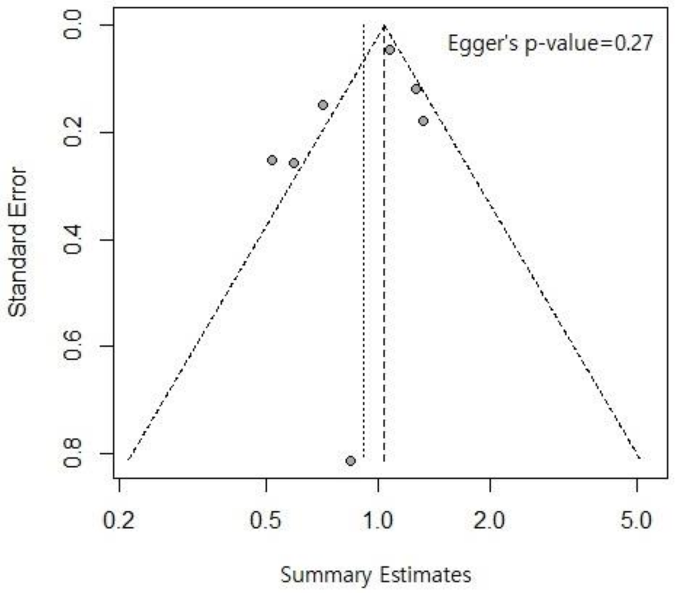

Summary estimates (95% CI) were measured by fixed-effect models if I^2 was <30% and P for heterogeneity was >0.05; otherwise, the summary estimates (95% CI) were measured by random-effect models. Forest and funnel plots were generated by "meta" package in R version 3.4.1. CHD, coronary heart disease; MHT, menopausal hormone therapy; SE, summary estimates.

Supplementary Figure S2.8. Pooled results of MHT and angina in the observational studies: (a) forest plot.

(a)

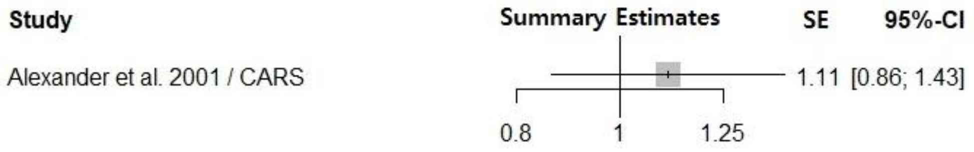

Summary estimates (95% CI) were measured by fixed-effect models if I^2 was <30% and P for heterogeneity was >0.05; otherwise, the summary estimates (95% CI) were measured by random-effect models. Forest and funnel plots were generated by "meta" package in R version 3.4.1. CHD, coronary heart disease; MHT, menopausal hormone therapy; SE, summary estimates.

Supplementary Figure S3.1.1. MHT and all-cause death in RCTs: subgroup results by regimen type.

(a) estrogen only

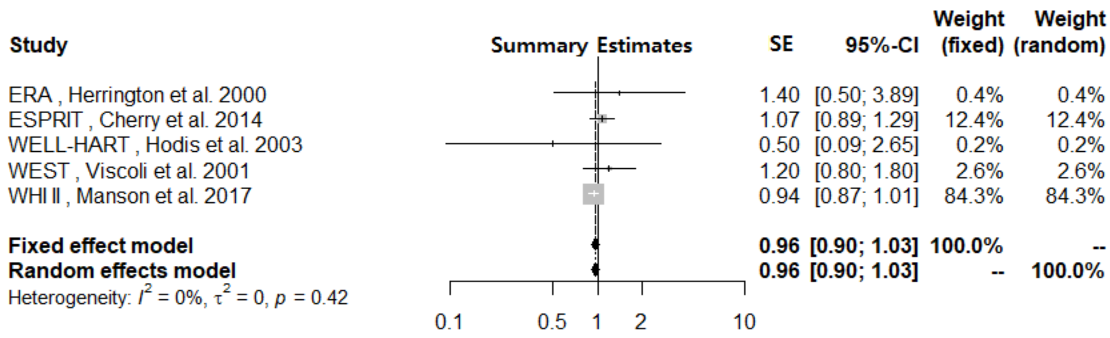

(b) combine EP

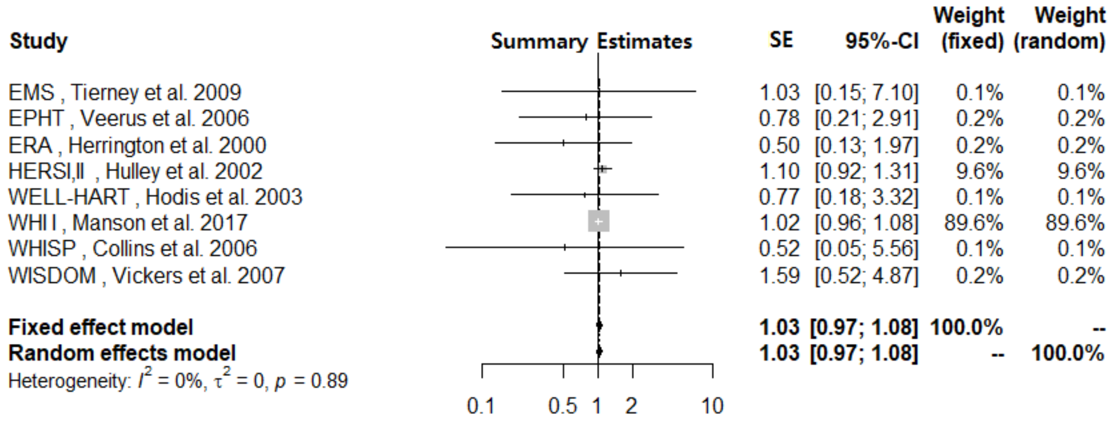

Summary estimates (95% CI) were measured by fixed-effect models if I^2 was <30% and P for heterogeneity was >0.05; otherwise, the summary estimates (95% CI) were measured by random-effect models. Forest and funnel plots were generated by "meta" package in R version 3.4.1. MHT, menopausal hormone therapy; RCTs, randomized controlled trials; SE, summary estimates.

Supplementary Figure S3.1.2. MHT and all-cause death in RCTs: subgroup results by duration of use.

(a) duration < 5 years

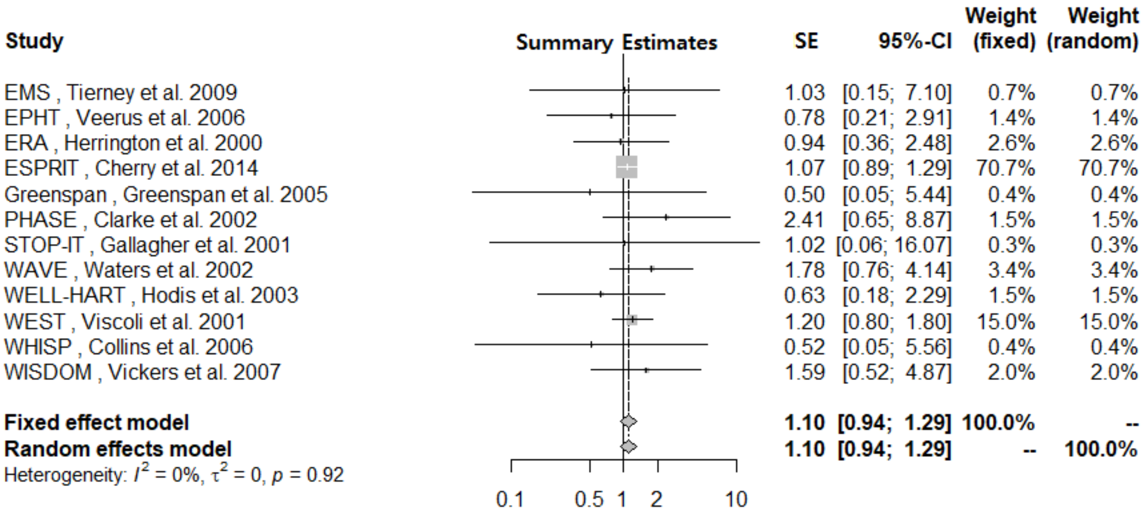

(b) duration ≥ 5 years

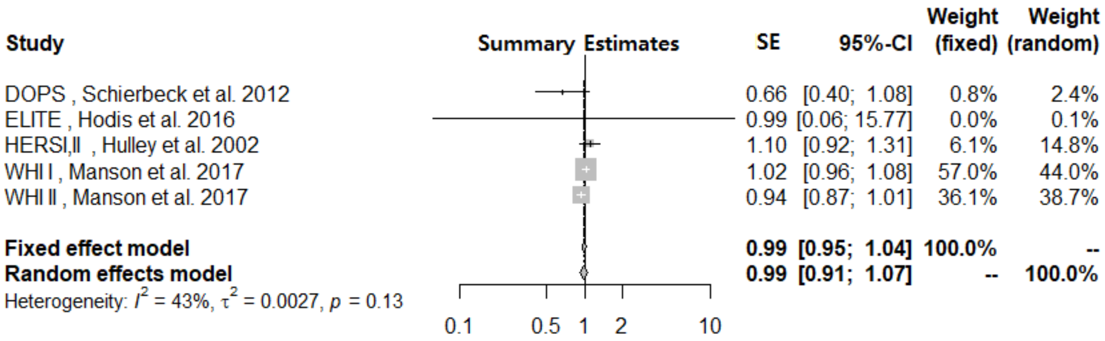

Summary estimates (95% CI) were measured by fixed-effect models if I^2 was <30% and P for heterogeneity was >0.05; otherwise, the summary estimates (95% CI) were measured by random-effect models. Forest and funnel plots were generated by "meta" package in R version 3.4.1. MHT, menopausal hormone therapy; RCTs, randomized controlled trials; SE, summary estimates.

Supplementary Figure S3.1.3. MHT and all-cause death in RCTs: subgroup results by timing of initiation.

(a) early users

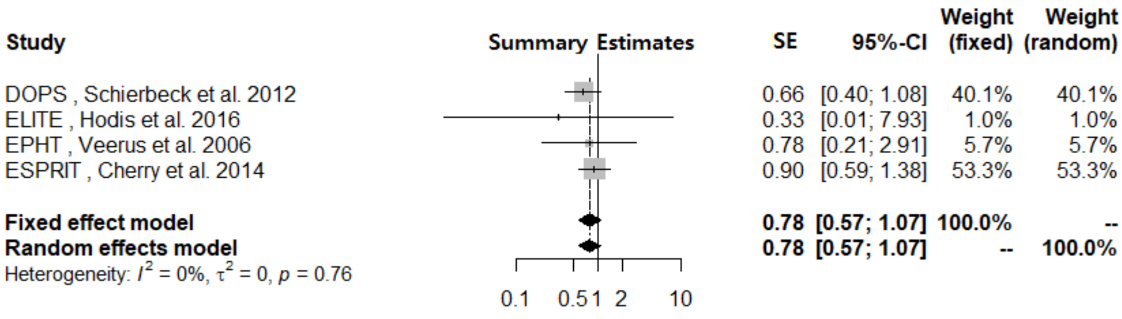

(b) late users

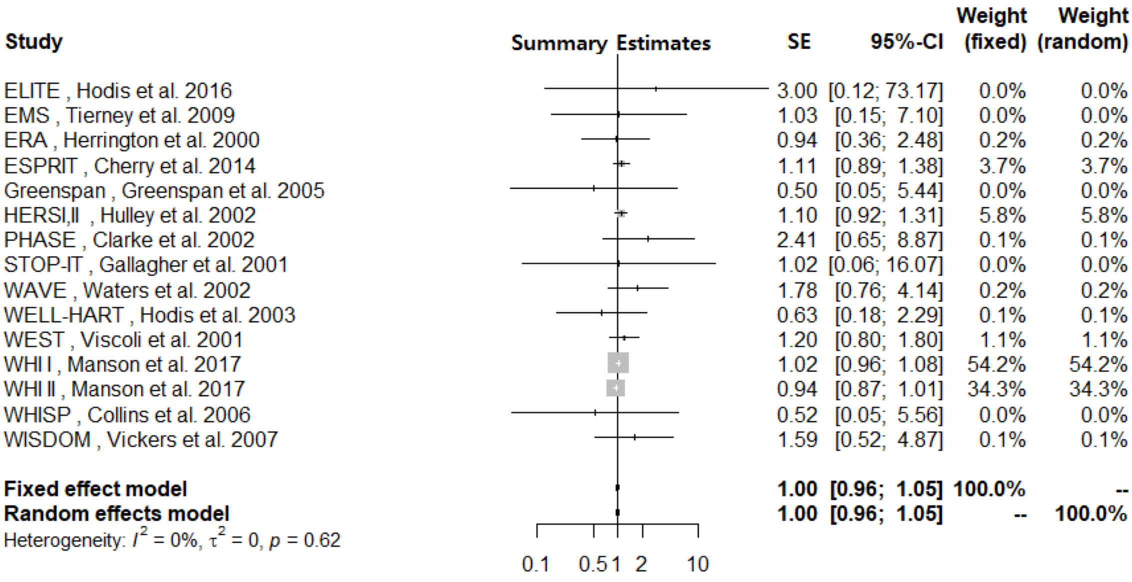

Summary estimates (95% CI) were measured by fixed-effect models if I^2 was <30% and P for heterogeneity was >0.05; otherwise, the summary estimates (95% CI) were measured by random-effect models. Forest and funnel plots were generated by "meta" package in R version 3.4.1. MHT, menopausal hormone therapy; RCTs, randomized controlled trials; SE, summary estimates.

Supplementary Figure S3.1.4. MHT and all-cause death in RCTs: subgroup results by underlying disease.

(a) women with diseases

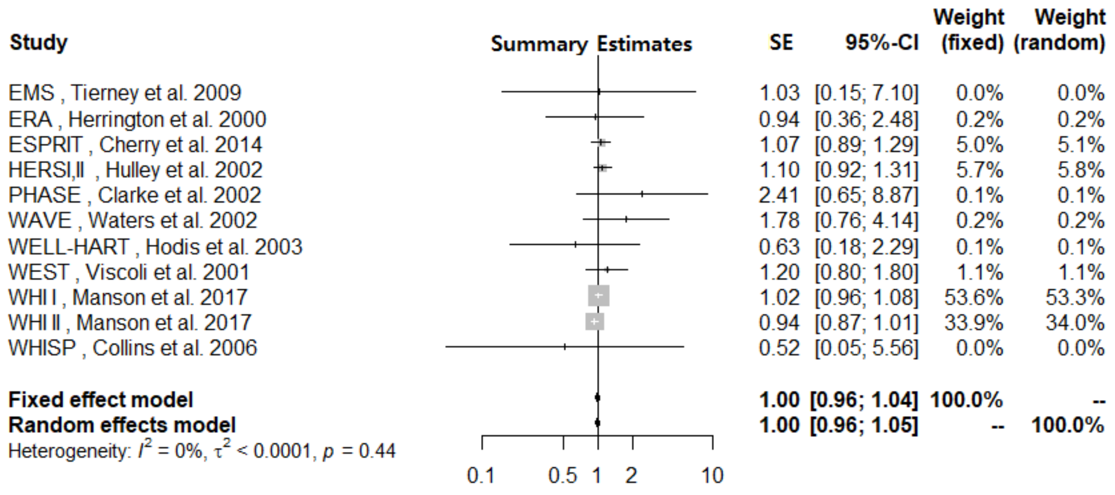

(b) women without diseases (relatively healthy)

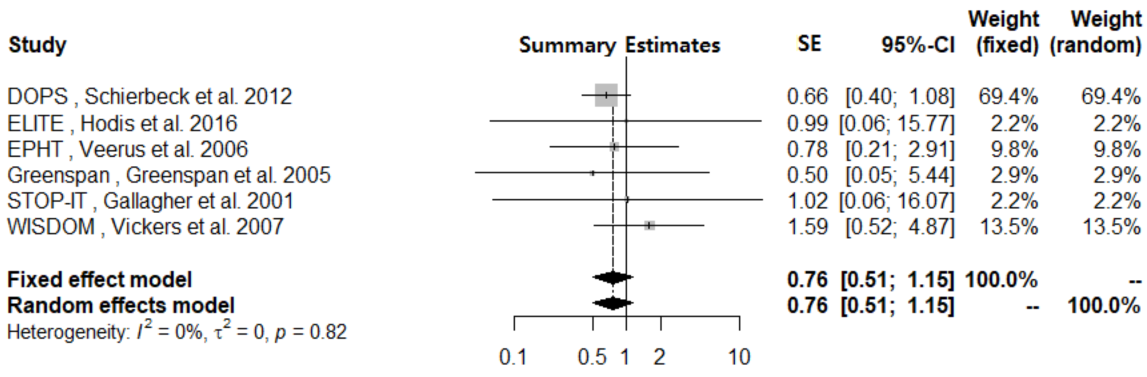

Summary estimates (95% CI) were measured by fixed-effect models if I^2 was <30% and P for heterogeneity was >0.05; otherwise, the summary estimates (95% CI) were measured by random-effect models. Forest and funnel plots were generated by "meta" package in R version 3.4.1. MHT, menopausal hormone therapy; RCTs, randomized controlled trials; SE, summary estimates.

Supplementary Figure S3.2.1. MHT and cardiovascular death in RCTs: subgroup results by regimen type.

(a) estrogen only

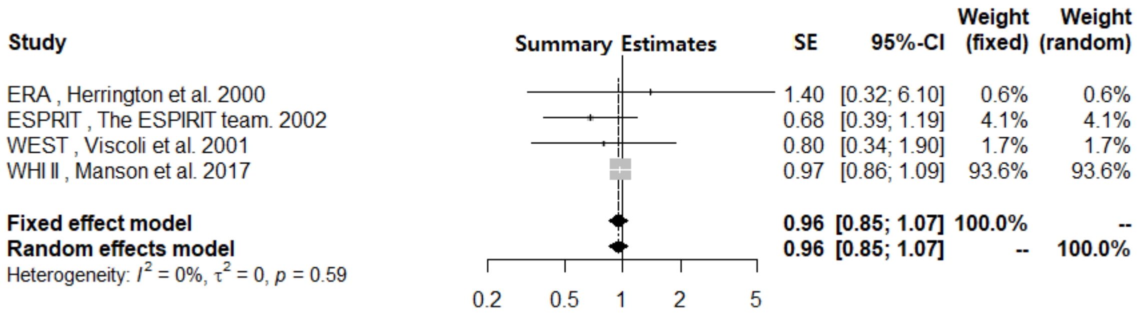

(b) combined EP

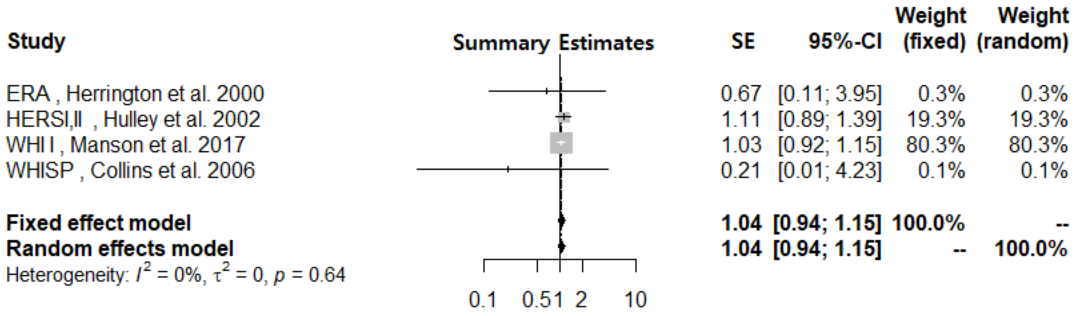

Summary estimates (95% CI) were measured by fixed-effect models if I^2 was <30% and P for heterogeneity was >0.05; otherwise, the summary estimates (95% CI) were measured by random-effect models. Forest and funnel plots were generated by "meta" package in R version 3.4.1. MHT, menopausal hormone therapy; RCTs, randomized controlled trials; SE, summary estimates.

Supplementary Figure S3.2.2. MHT and cardiovascular death in RCTs: subgroup results by duration of use.

(a) duration < 5 years

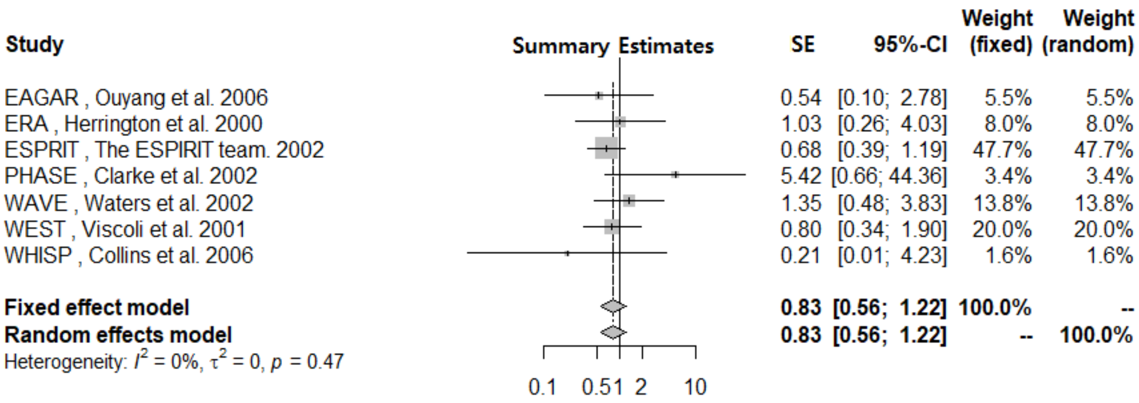

(b) duration ≥ 5 years

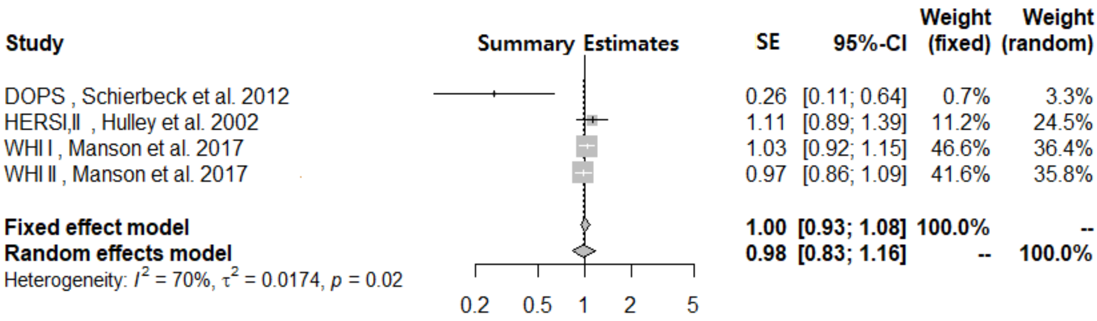

Summary estimates (95% CI) were measured by fixed-effect models if I^2 was <30% and P for heterogeneity was >0.05; otherwise, the summary estimates (95% CI) were measured by random-effect models. Forest and funnel plots were generated by "meta" package in R version 3.4.1. MHT, menopausal hormone therapy; RCTs, randomized controlled trials; SE, summary estimates.

Supplementary Figure S3.2.3. MHT and cardiovascular death in RCTs: subgroup results by timing of initiation.

(a) early users

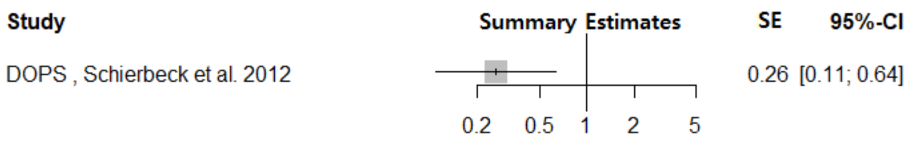

(b) late users

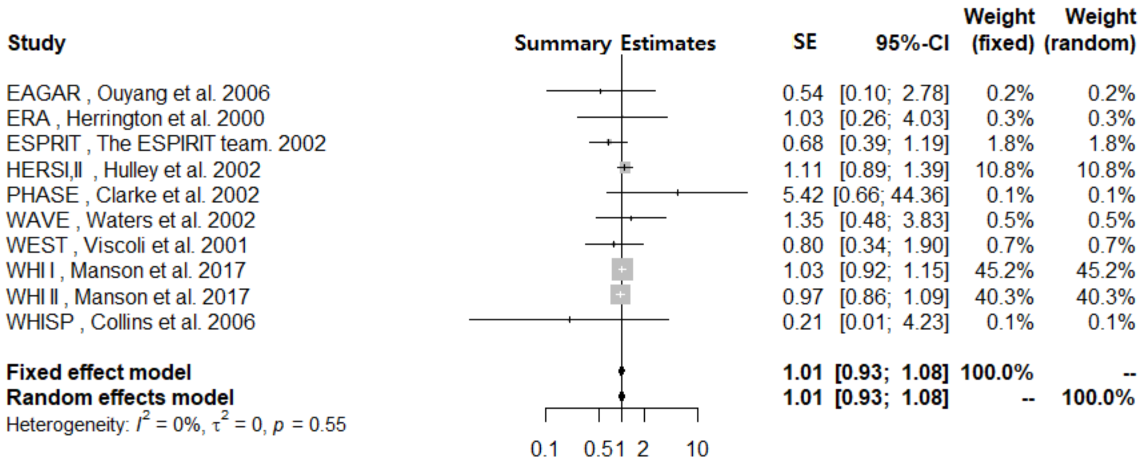

Summary estimates (95% CI) were measured by fixed-effect models if I^2 was <30% and P for heterogeneity was >0.05; otherwise, the summary estimates (95% CI) were measured by random-effect models. Forest and funnel plots were generated by "meta" package in R version 3.4.1. MHT, menopausal hormone therapy; RCTs, randomized controlled trials; SE, summary estimates.

Supplementary Figure S3.2.4. MHT and cardiovascular death in RCTs: subgroup results by underlying disease.

(a) women with diseases

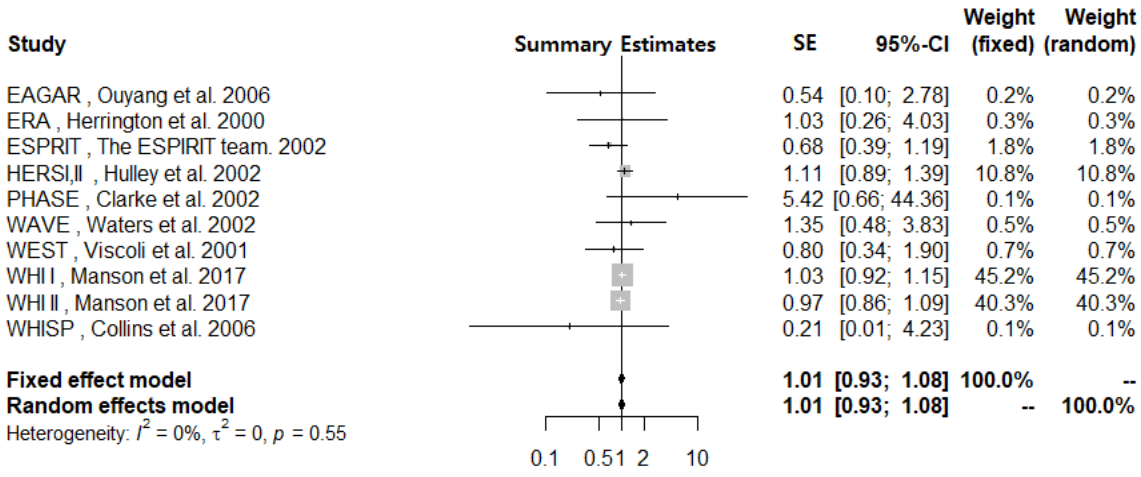

(b) women without diseases (relatively healthy)

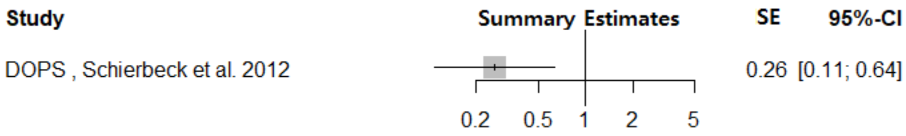

Summary estimates (95% CI) were measured by fixed-effect models if I^2 was <30% and P for heterogeneity was >0.05; otherwise, the summary estimates (95% CI) were measured by random-effect models. Forest and funnel plots were generated by "meta" package in R version 3.4.1. MHT, menopausal hormone therapy; RCTs, randomized controlled trials; SE, summary estimates.

Supplementary Figure S3.3.1. MHT and stroke in RCTs: subgroup results by regimen type.

(a) estrogen only

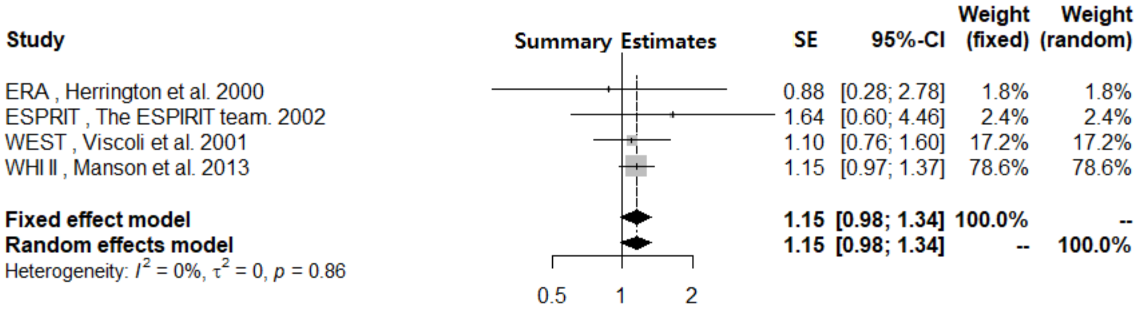

(b) combined EP

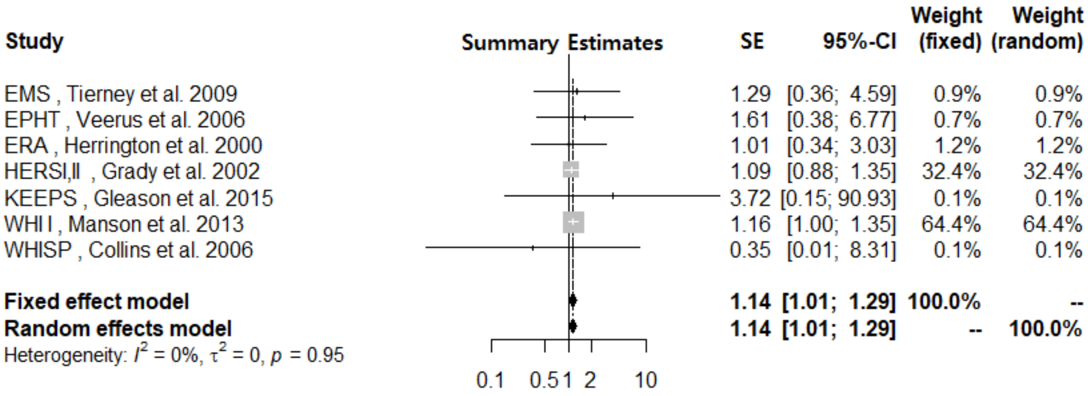

Summary estimates (95% CI) were measured by fixed-effect models if I^2 was <30% and P for heterogeneity was >0.05; otherwise, the summary estimates (95% CI) were measured by random-effect models. Forest and funnel plots were generated by "meta" package in R version 3.4.1. MHT, menopausal hormone therapy; RCTs, randomized controlled trials; SE, summary estimates.

Supplementary Figure S3.3.2. MHT and stroke in RCTs: subgroup results by duration of use.

(a) duration < 5 years

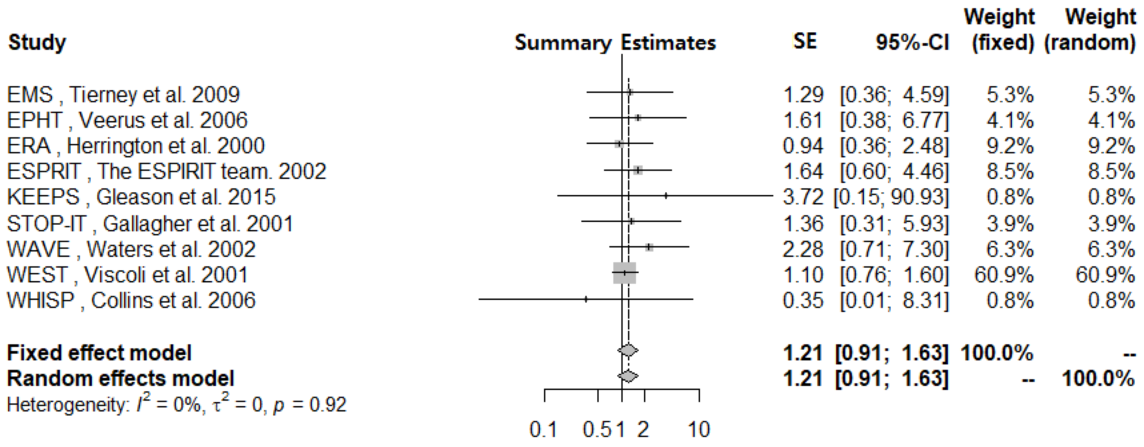

(b) duration ≥ 5 years

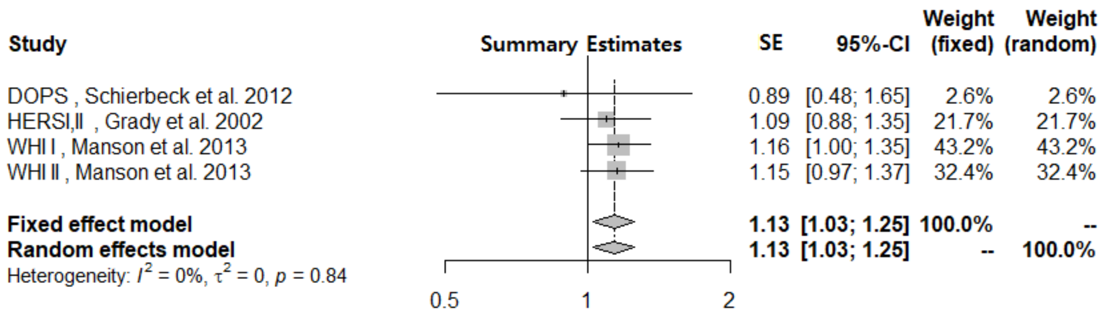

Summary estimates (95% CI) were measured by fixed-effect models if I^2 was <30% and P for heterogeneity was >0.05; otherwise, the summary estimates (95% CI) were measured by random-effect models. Forest and funnel plots were generated by "meta" package in R version 3.4.1. MHT, menopausal hormone therapy; RCTs, randomized controlled trials; SE, summary estimates.

Supplementary Figure S3.3.3. MHT and stroke in RCTs: subgroup results by timing of initiation.

(a) early users

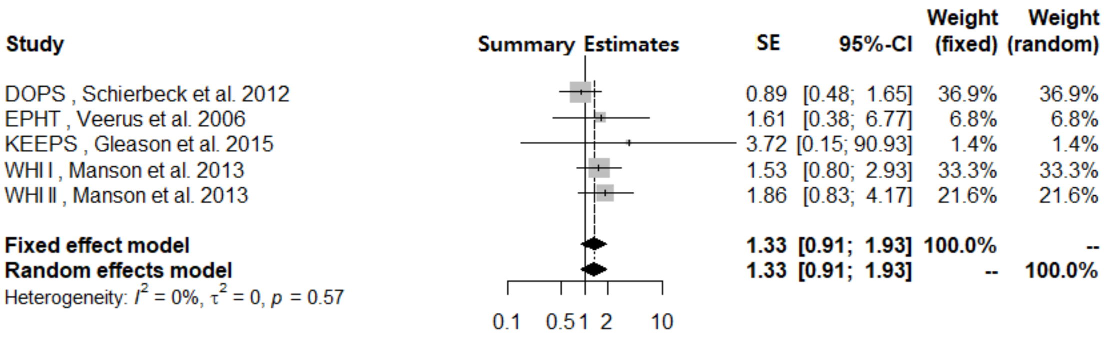

(b) late users

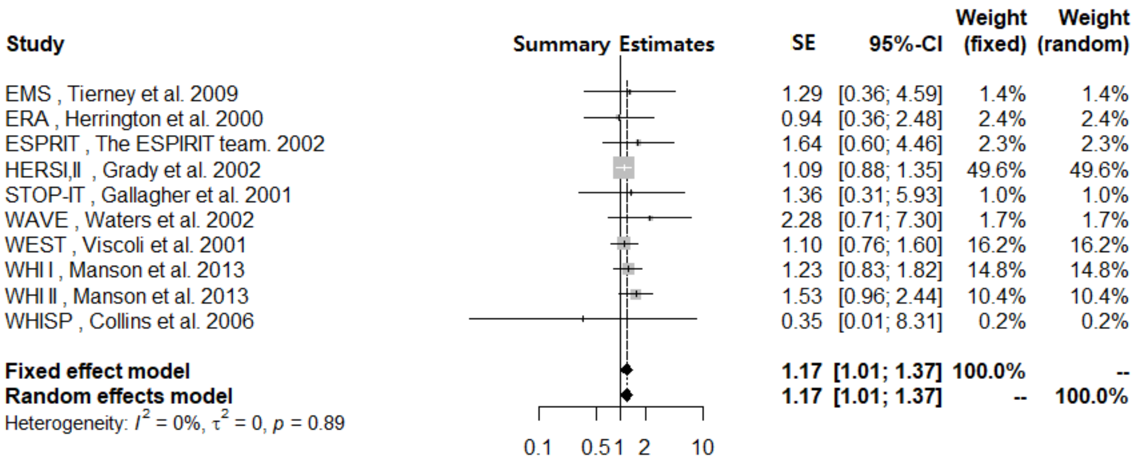

Summary estimates (95% CI) were measured by fixed-effect models if I^2 was <30% and P for heterogeneity was >0.05; otherwise, the summary estimates (95% CI) were measured by random-effect models. Forest and funnel plots were generated by "meta" package in R version 3.4.1. MHT, menopausal hormone therapy; RCTs, randomized controlled trials; SE, summary estimates.

Supplementary Figure S3.3.4. MHT and stroke in RCTs: subgroup results by underlying disease.

(a) women with diseases

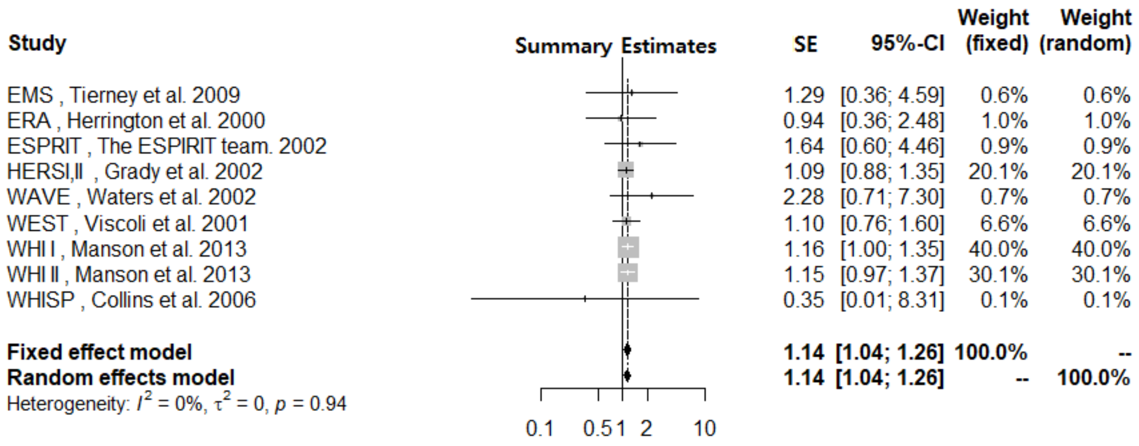

(b) women without diseases (relatively healthy)

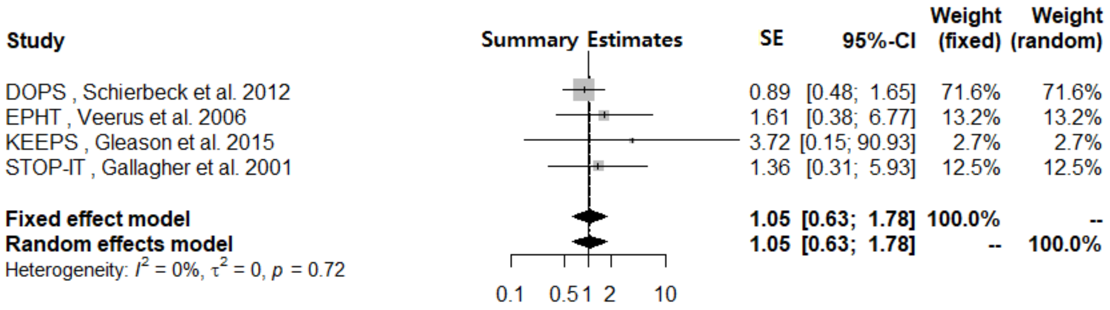

Summary estimates (95% CI) were measured by fixed-effect models if I^2 was <30% and P for heterogeneity was >0.05; otherwise, the summary estimates (95% CI) were measured by random-effect models. Forest and funnel plots were generated by "meta" package in R version 3.4.1. MHT, menopausal hormone therapy; RCTs, randomized controlled trials; SE, summary estimates.

Supplementary Figure S3.4.1. MHT and VTE in RCTs: subgroup results by regimen type.

(a) estrogen only

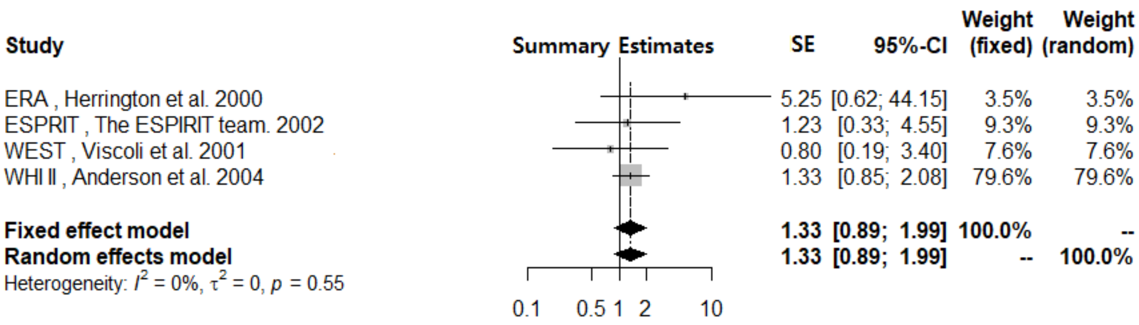

(b) combined EP

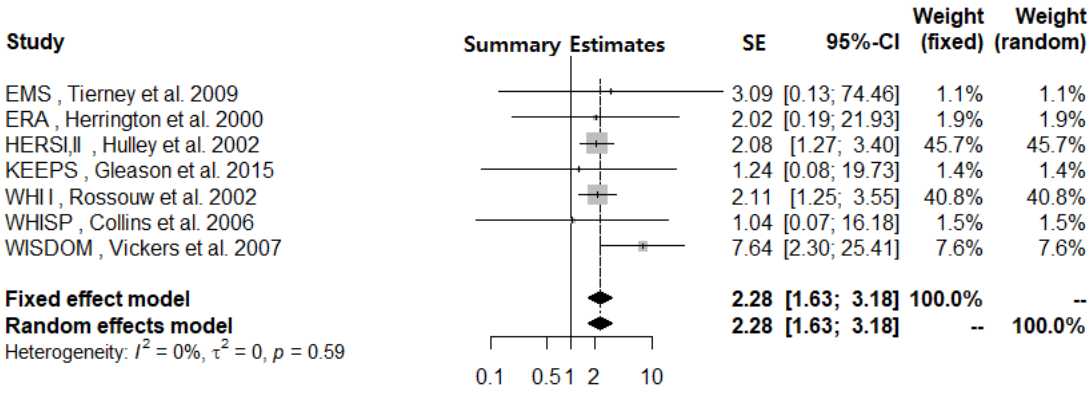

Summary estimates (95% CI) were measured by fixed-effect models if I^2 was <30% and P for heterogeneity was >0.05; otherwise, the summary estimates (95% CI) were measured by random-effect models. Forest and funnel plots were generated by "meta" package in R version 3.4.1. MHT, menopausal hormone therapy; RCTs, randomized controlled trials; SE, summary estimates; VTE, venous thromboembolism.

Supplementary Figure S3.4.2. MHT and VTE in RCTs: subgroup results by duration of use.

(a) duration < 5 years

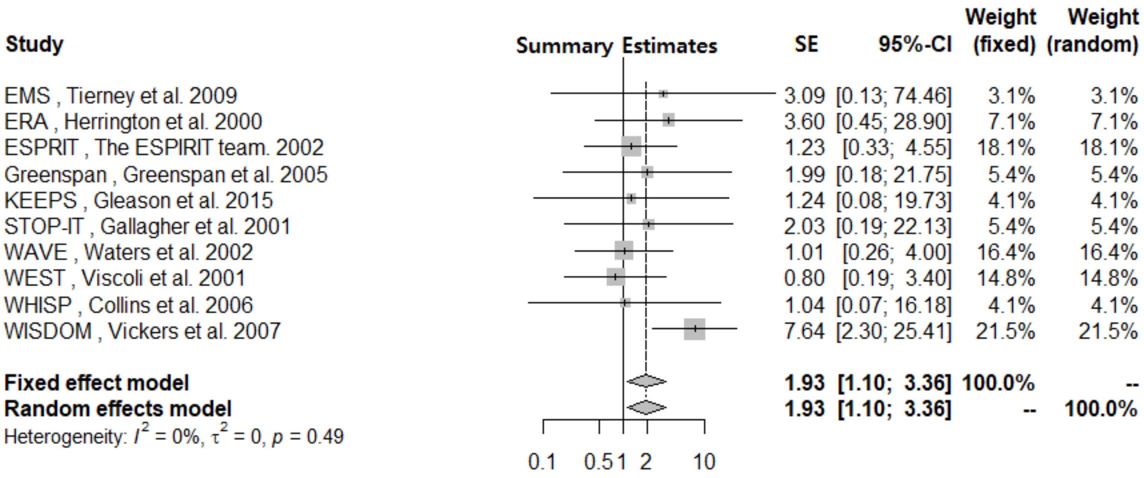

(b) duration ≥ 5 years

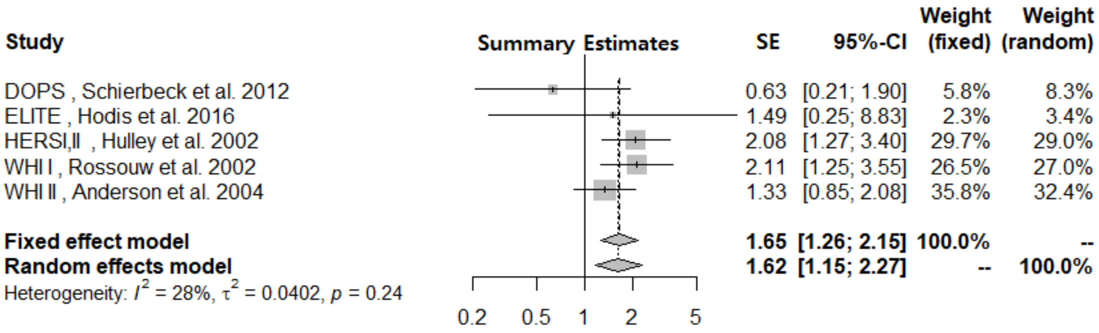

Summary estimates (95% CI) were measured by fixed-effect models if I^2 was <30% and P for heterogeneity was >0.05; otherwise, the summary estimates (95% CI) were measured by random-effect models. Forest and funnel plots were generated by "meta" package in R version 3.4.1. MHT, menopausal hormone therapy; RCTs, randomized controlled trials; SE, summary estimates; VTE, venous thromboembolism.

Supplementary Figure S3.4.3. MHT and VTE in RCTs: subgroup results by timing of initiation.

(a) early users

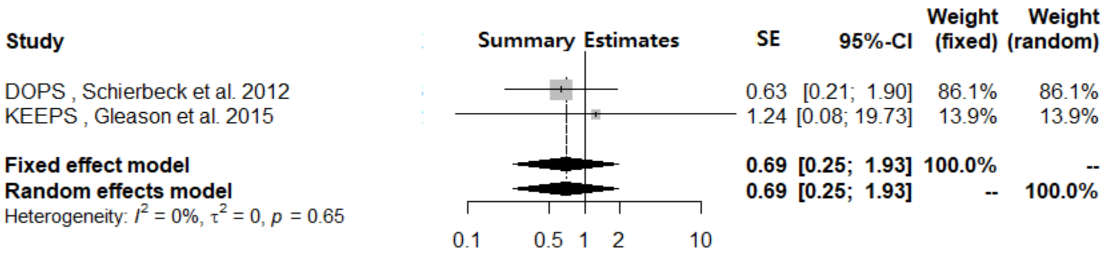

(b) late users

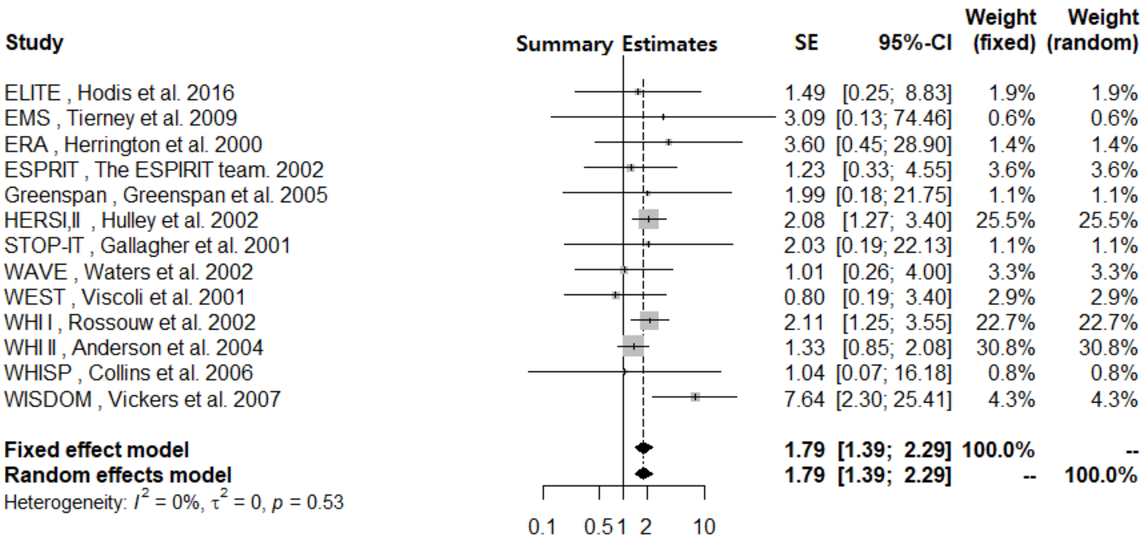

Summary estimates (95% CI) were measured by fixed-effect models if I^2 was <30% and P for heterogeneity was >0.05; otherwise, the summary estimates (95% CI) were measured by random-effect models. Forest and funnel plots were generated by "meta" package in R version 3.4.1. MHT, menopausal hormone therapy; RCTs, randomized controlled trials; SE, summary estimates; VTE, venous thromboembolism.

Supplementary Figure S3.4.4. MHT and VTE in RCTs: subgroup results by underlying disease.

(a) women with diseases

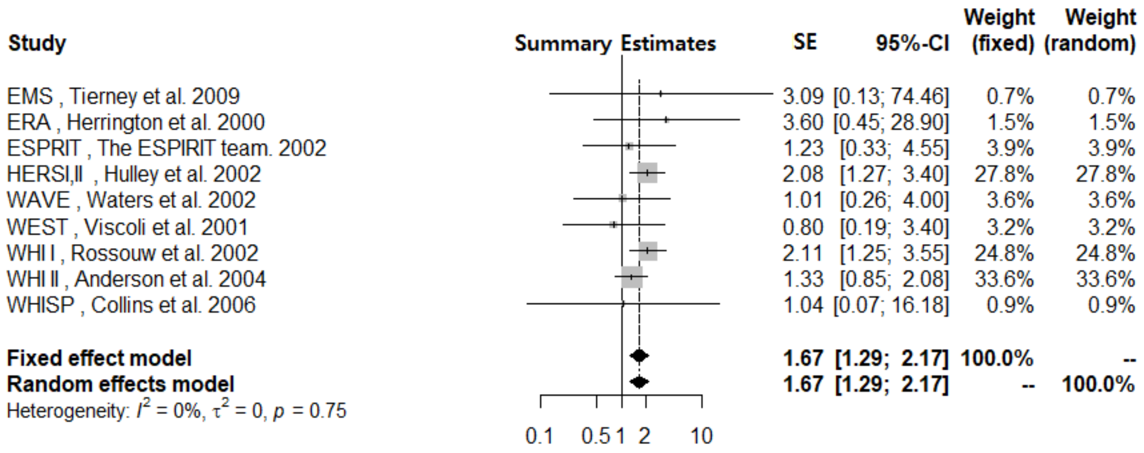

(b) women without diseases (relatively healthy)

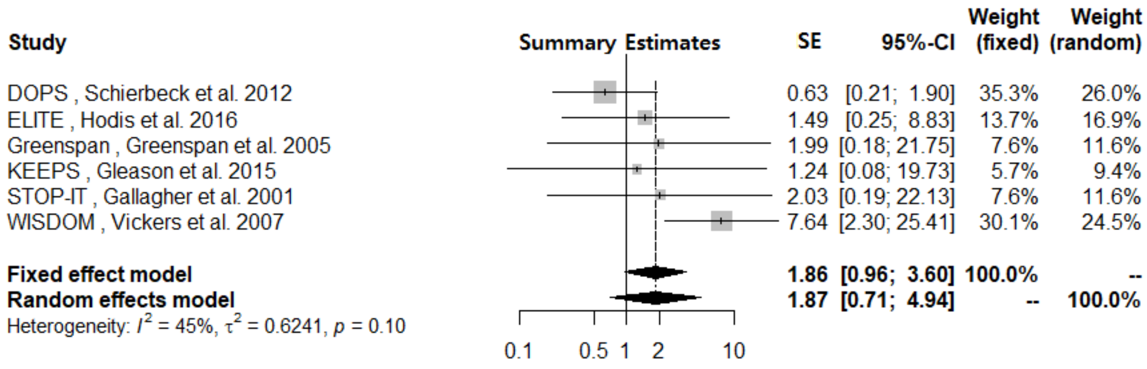

Summary estimates (95% CI) were measured by fixed-effect models if I^2 was <30% and P for heterogeneity was >0.05; otherwise, the summary estimates (95% CI) were measured by random-effect models. Forest and funnel plots were generated by "meta" package in R version 3.4.1. MHT, menopausal hormone therapy; RCTs, randomized controlled trials; SE, summary estimates; VTE, venous thromboembolism.

Supplementary Figure S3.5.1. MHT and PE in RCTs: subgroup results by regimen type.

(a) estrogen only

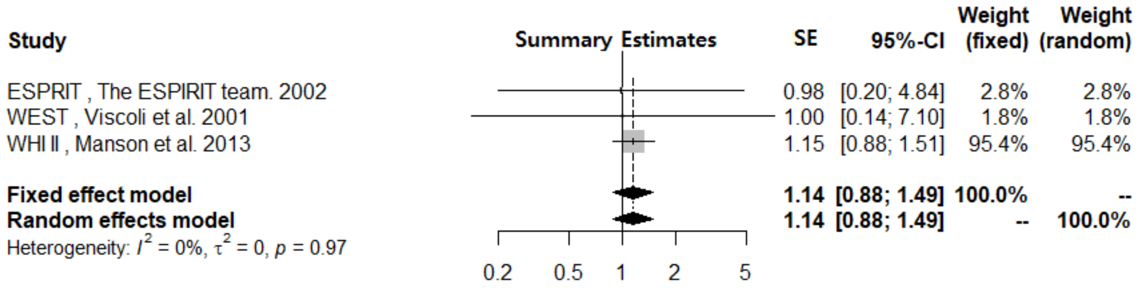

(b) combined EP

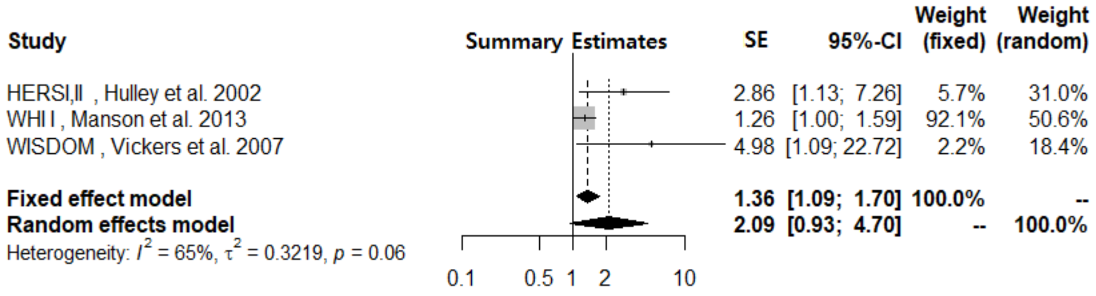

Summary estimates (95% CI) were measured by fixed-effect models if I^2 was <30% and P for heterogeneity was >0.05; otherwise, the summary estimates (95% CI) were measured by random-effect models. Forest and funnel plots were generated by "meta" package in R version 3.4.1. MHT, menopausal hormone therapy; PE, pulmonary embolism; RCTs, randomized controlled trials; SE, summary estimates.

Supplementary Figure S3.5.2. MHT and PE in RCTs: subgroup results by duration of use.

(a) duration < 5 years

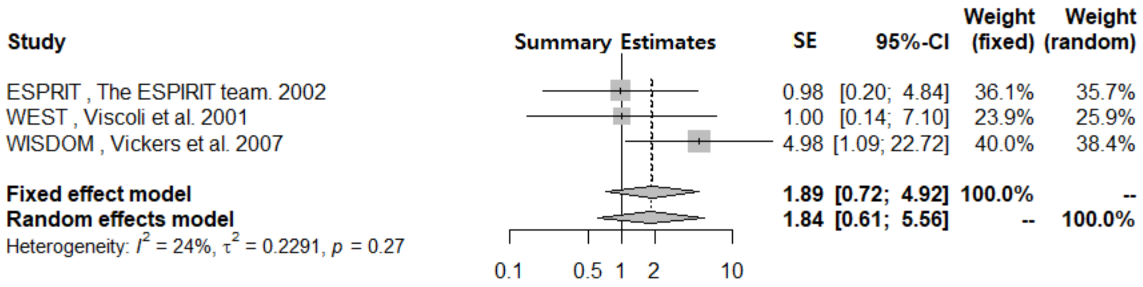

(b) duration ≥ 5 years

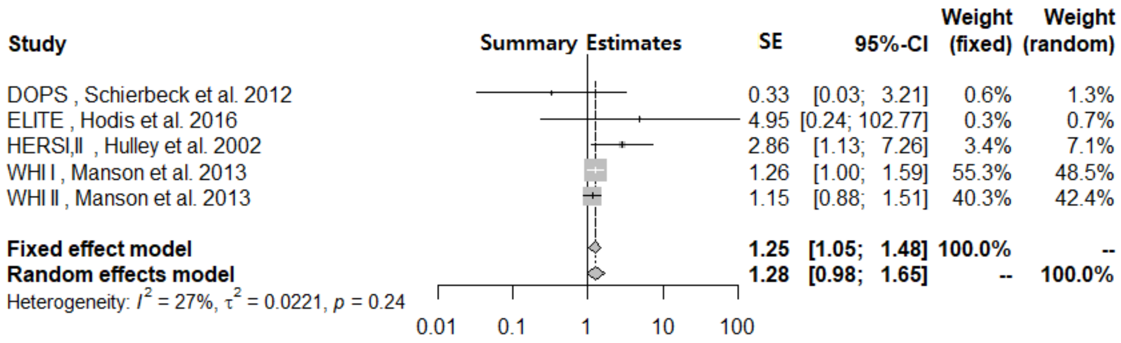

Summary estimates (95% CI) were measured by fixed-effect models if I^2 was <30% and P for heterogeneity was >0.05; otherwise, the summary estimates (95% CI) were measured by random-effect models. Forest and funnel plots were generated by "meta" package in R version 3.4.1. MHT, menopausal hormone therapy; PE, pulmonary embolism; RCTs, randomized controlled trials; SE, summary estimates.

Supplementary Figure S3.5.3. MHT and PE in RCTs: subgroup results by timing of initiation.

(a) early users

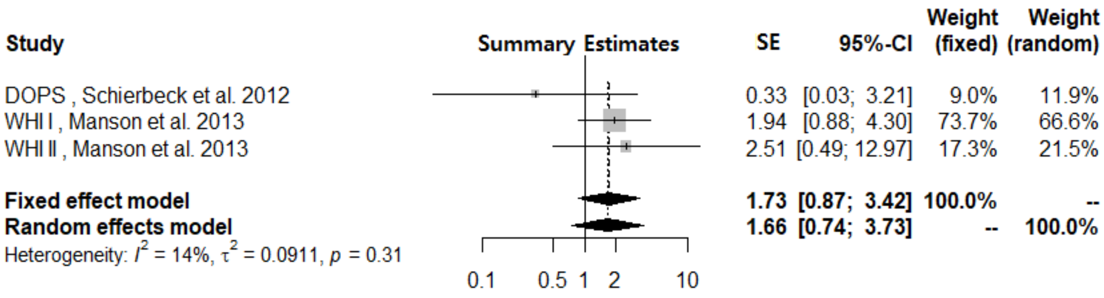

(b) late users

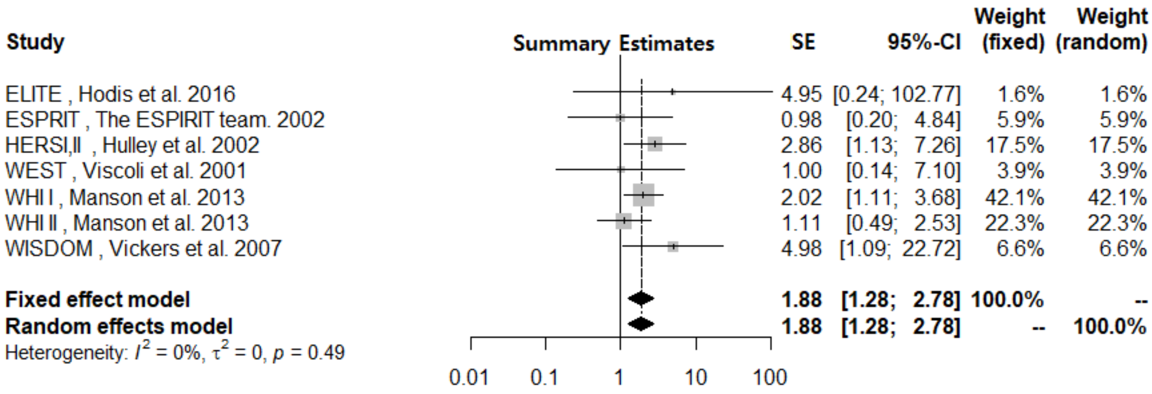

Summary estimates (95% CI) were measured by fixed-effect models if I^2 was <30% and P for heterogeneity was >0.05; otherwise, the summary estimates (95% CI) were measured by random-effect models. Forest and funnel plots were generated by "meta" package in R version 3.4.1. MHT, menopausal hormone therapy; PE, pulmonary embolism; RCTs, randomized controlled trials; SE, summary estimates.

Supplementary Figure S3.5.4. MHT and PE in RCTs: subgroup results by underlying disease.

(a) women with diseases

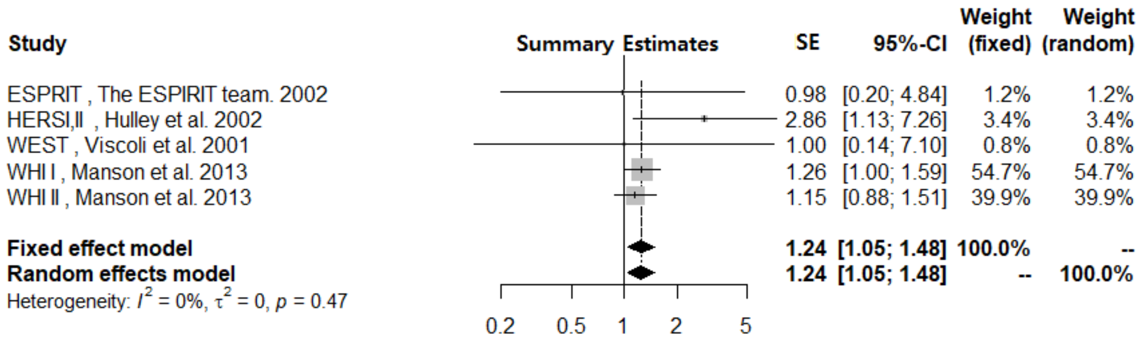

(b) women without diseases (relatively healthy)

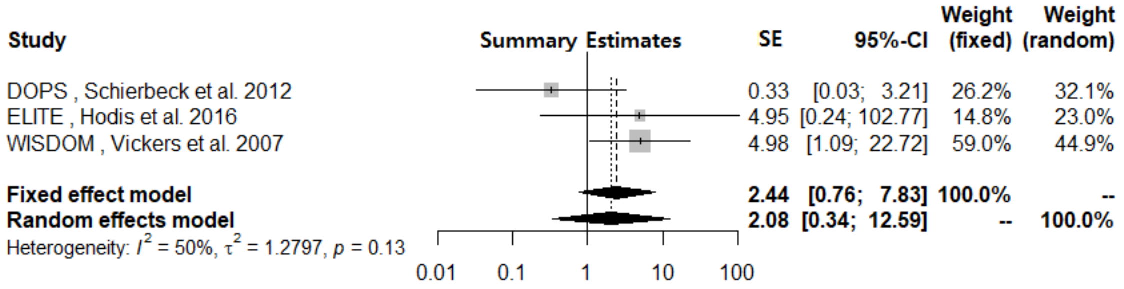

Summary estimates (95% CI) were measured by fixed-effect models if I^2 was <30% and P for heterogeneity was >0.05; otherwise, the summary estimates (95% CI) were measured by random-effect models. Forest and funnel plots were generated by "meta" package in R version 3.4.1. MHT, menopausal hormone therapy; PE, pulmonary embolism; RCTs, randomized controlled trials; SE, summary estimates.

Supplementary Figure S3.6.1. MHT and MI in RCTs: subgroup results by regimen type.

(a) estrogen only

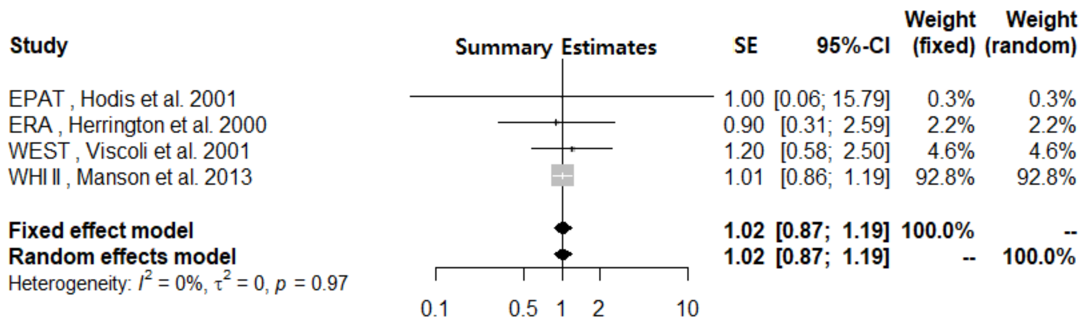

(b) combined EP

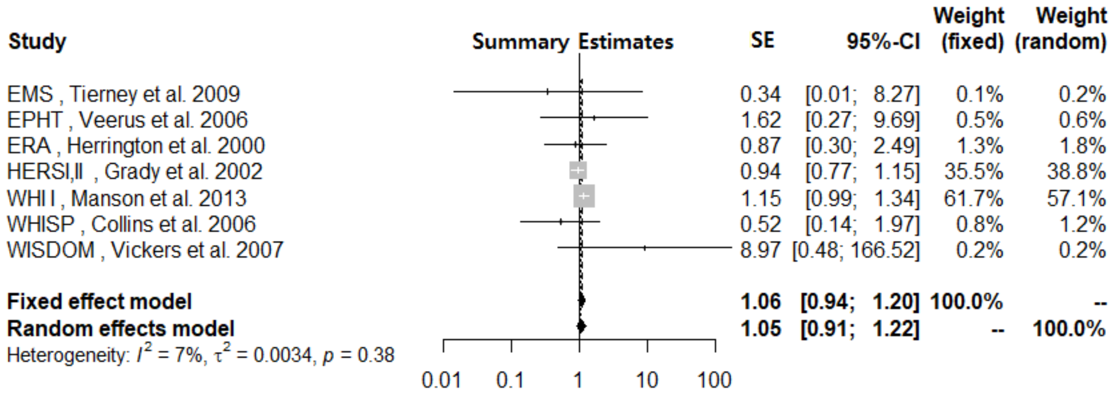

Summary estimates (95% CI) were measured by fixed-effect models if I^2 was <30% and P for heterogeneity was >0.05; otherwise, the summary estimates (95% CI) were measured by random-effect models. Forest and funnel plots were generated by "meta" package in R version 3.4.1. MHT, menopausal hormone therapy; PE, pulmonary embolism; RCTs, randomized controlled trials; SE, summary estimates.

Supplementary Figure S3.6.2. MHT and MI in RCTs: subgroup results by duration of use.

(a) duration < 5 years

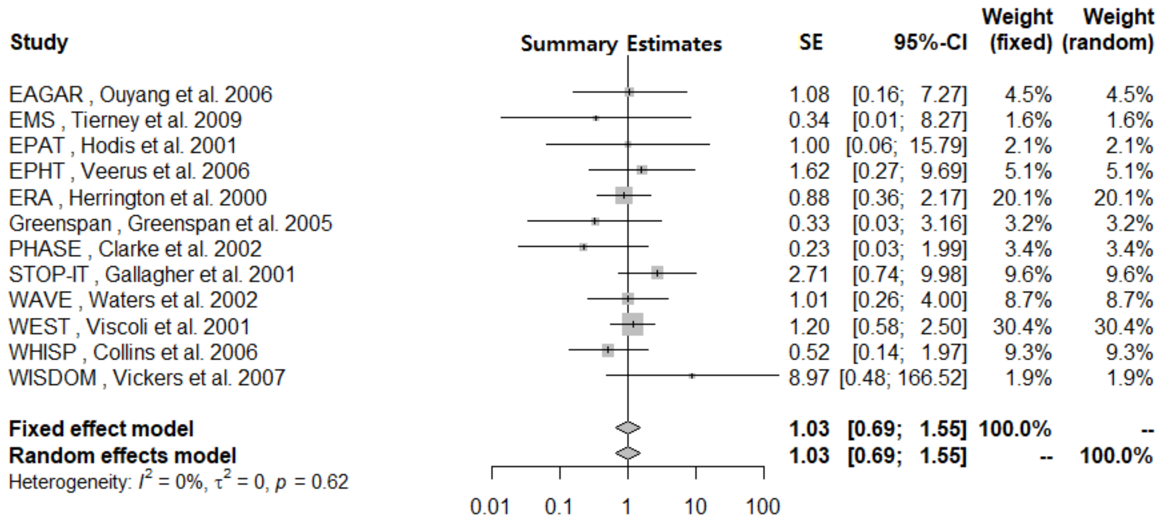

(b) duration ≥ 5 years

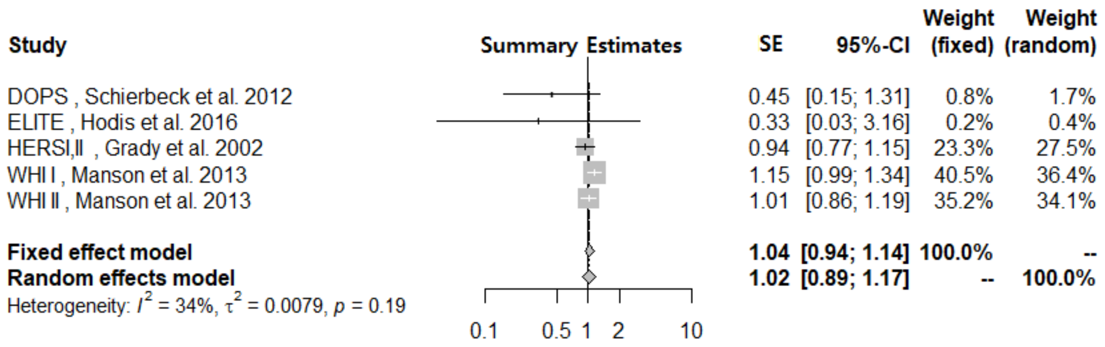

Summary estimates (95% CI) were measured by fixed-effect models if I^2 was <30% and P for heterogeneity was >0.05; otherwise, the summary estimates (95% CI) were measured by random-effect models. Forest and funnel plots were generated by "meta" package in R version 3.4.1. MHT, menopausal hormone therapy; MI, myocardial infarction; RCTs, randomized controlled trials; SE, summary estimates.

Supplementary Figure S3.6.3. MHT and MI in RCTs: subgroup results by timing of initiation.

(a) early users

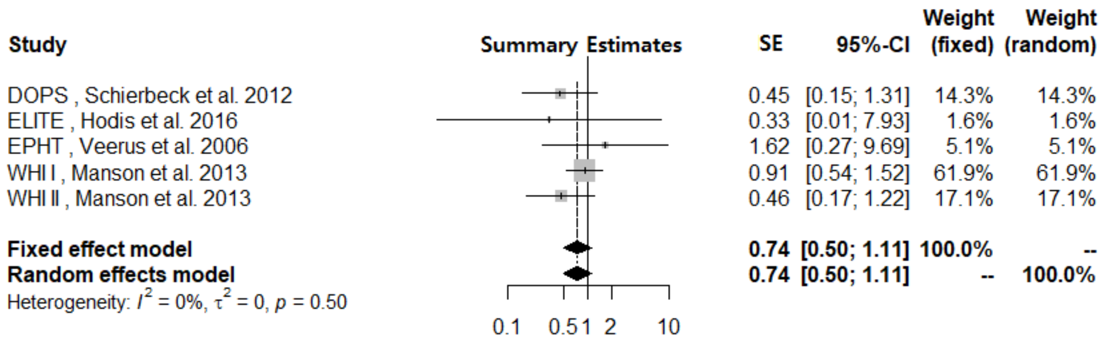

(b) late users

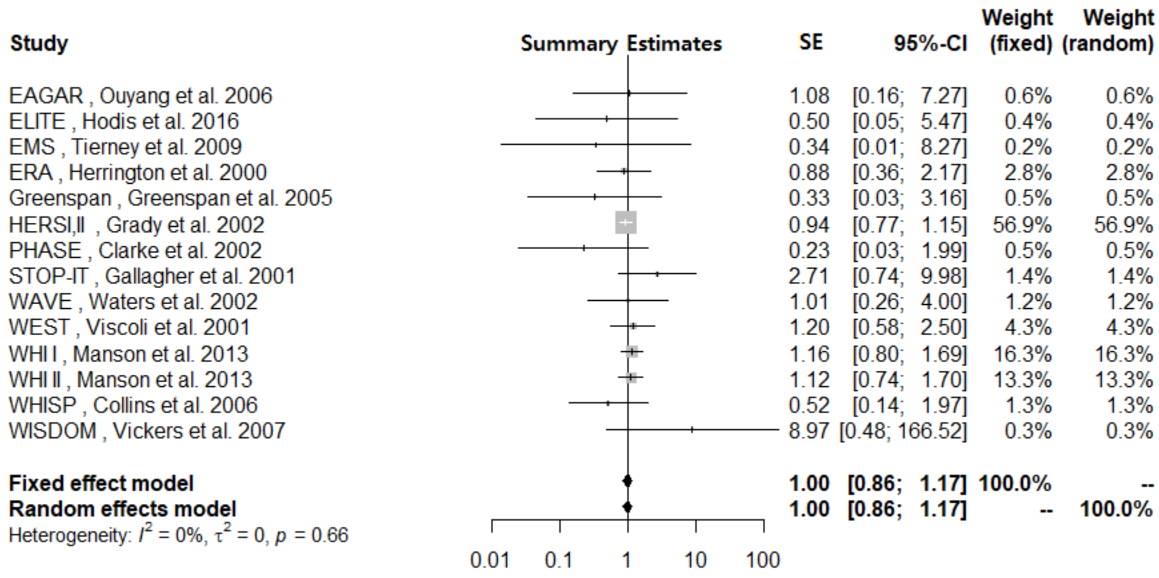

Summary estimates (95% CI) were measured by fixed-effect models if I^2 was <30% and P for heterogeneity was >0.05; otherwise, the summary estimates (95% CI) were measured by random-effect models. Forest and funnel plots were generated by "meta" package in R version 3.4.1. MHT, menopausal hormone therapy; MI, myocardial infarction; RCTs, randomized controlled trials; SE, summary estimates.

Supplementary Figure S3.6.4. MHT and MI in RCTs: subgroup results by underlying disease.

(a) women with diseases

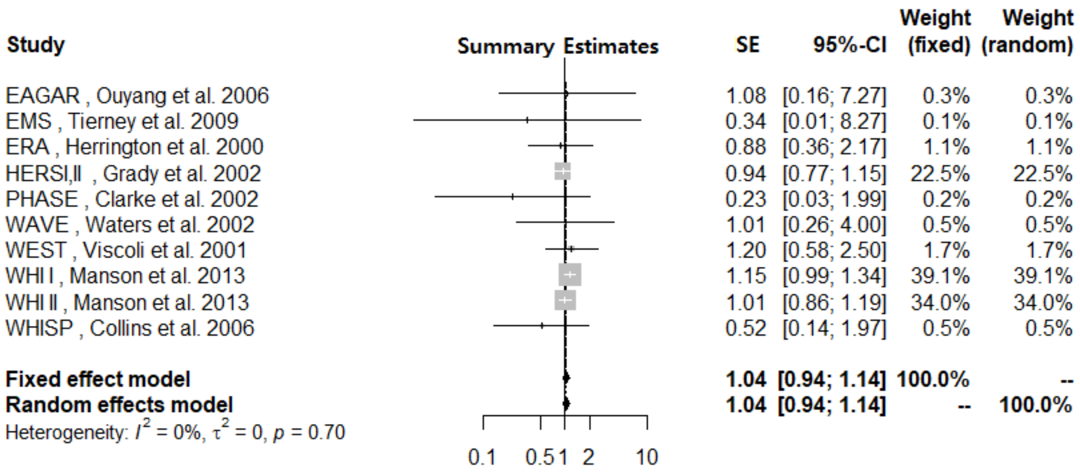

(b) women without diseases (relatively healthy)

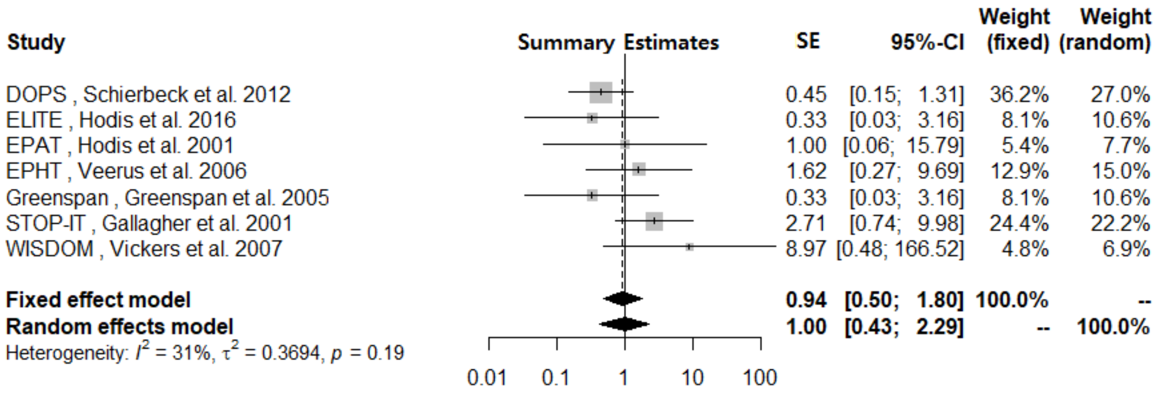

Summary estimates (95% CI) were measured by fixed-effect models if I^2 was <30% and P for heterogeneity was >0.05; otherwise, the summary estimates (95% CI) were measured by random-effect models. Forest and funnel plots were generated by "meta" package in R version 3.4.1. MHT, menopausal hormone therapy; MI, myocardial infarction; RCTs, randomized controlled trials; SE, summary estimates.

Supplementary Figure S3.7.1. MHT and CHD in RCTs: subgroup results by regimen type.

(a) estrogen only

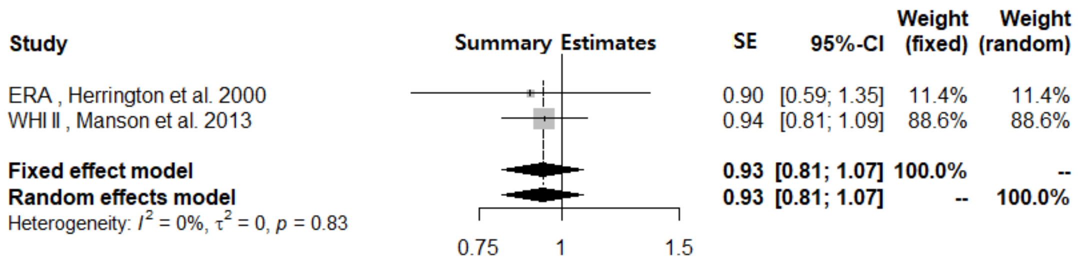

(b) combined EP

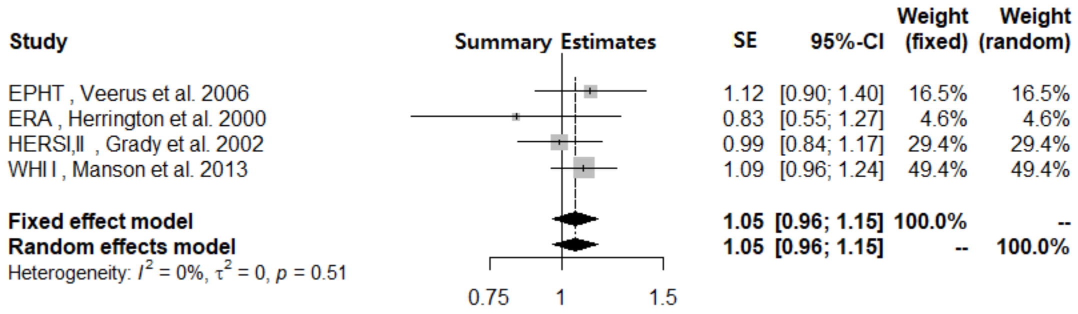

Summary estimates (95% CI) were measured by fixed-effect models if I^2 was <30% and P for heterogeneity was >0.05; otherwise, the summary estimates (95% CI) were measured by random-effect models. Forest and funnel plots were generated by "meta" package in R version 3.4.1. CHD, coronary heart disease; MHT, menopausal hormone therapy; RCTs, randomized controlled trials; SE, summary estimates.

Supplementary Figure S3.7.2. MHT and CHD in RCTs: subgroup results by duration of use.

(a) duration < 5 years

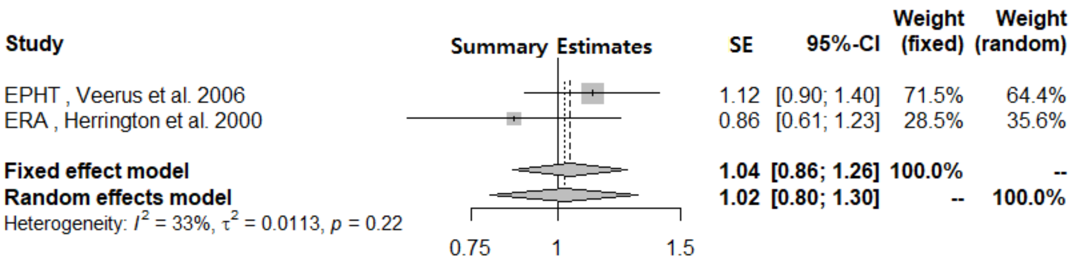

(b) duration ≥ 5 years

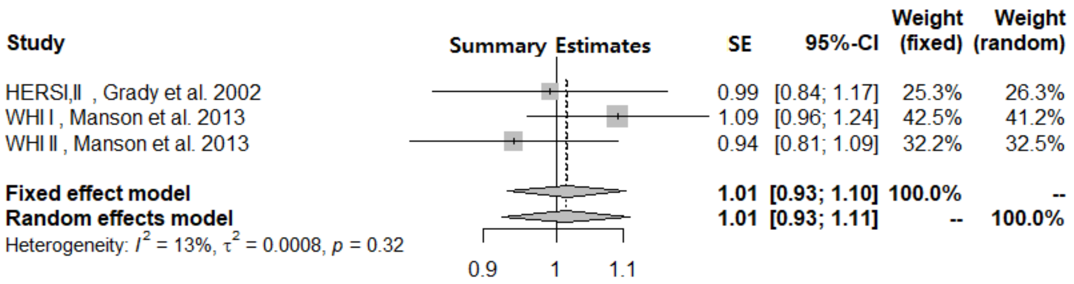

Summary estimates (95% CI) were measured by fixed-effect models if I^2 was <30% and P for heterogeneity was >0.05; otherwise, the summary estimates (95% CI) were measured by random-effect models. Forest and funnel plots were generated by "meta" package in R version 3.4.1. CHD, coronary heart disease; MHT, menopausal hormone therapy; RCTs, randomized controlled trials; SE, summary estimates.

Supplementary Figure S3.7.3. MHT and CHD in RCTs: subgroup results by timing of initiation.

(a) early users

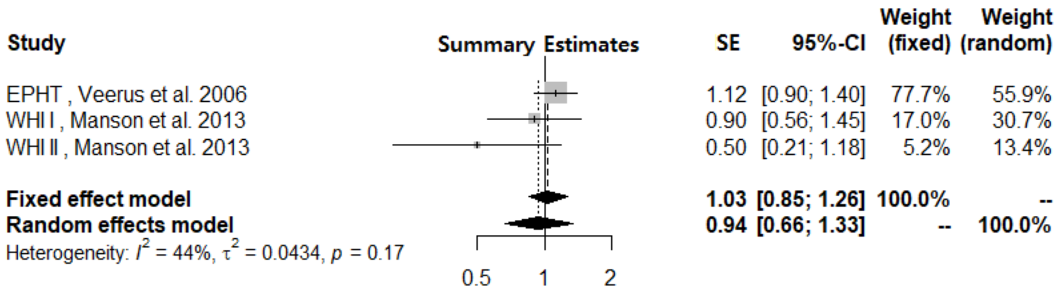

(b) late users

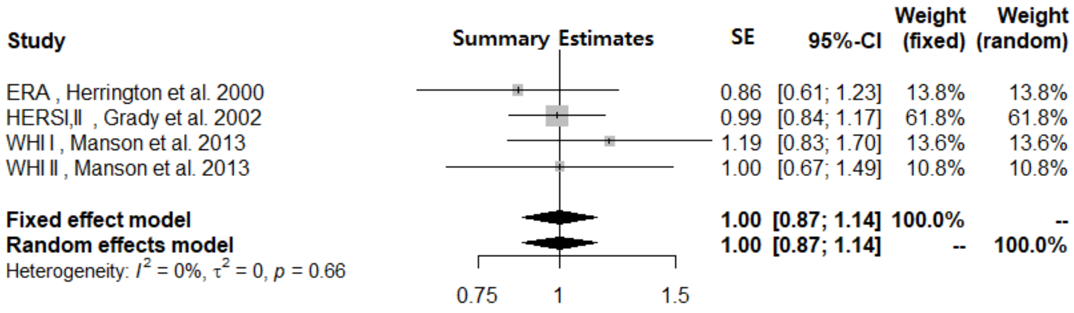

Summary estimates (95% CI) were measured by fixed-effect models if I^2 was <30% and P for heterogeneity was >0.05; otherwise, the summary estimates (95% CI) were measured by random-effect models. Forest and funnel plots were generated by "meta" package in R version 3.4.1. CHD, coronary heart disease; MHT, menopausal hormone therapy; RCTs, randomized controlled trials; SE, summary estimates.

Supplementary Figure S3.7.4. MHT and CHD in RCTs: subgroup results by underlying disease.

(a) women with diseases

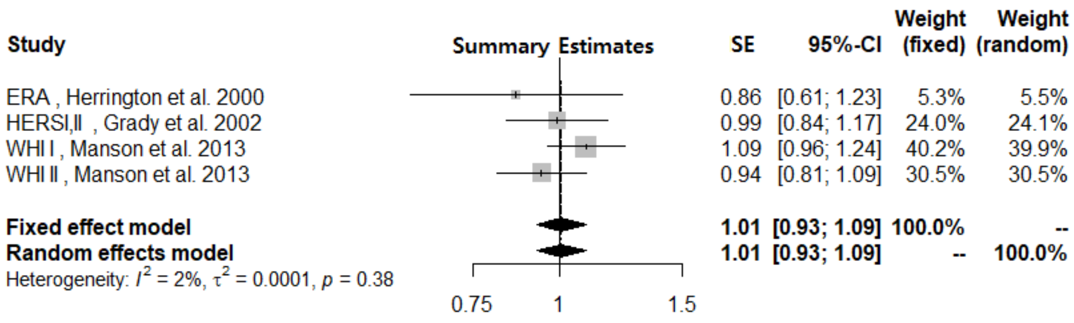

(b) women without diseases (relatively healthy)

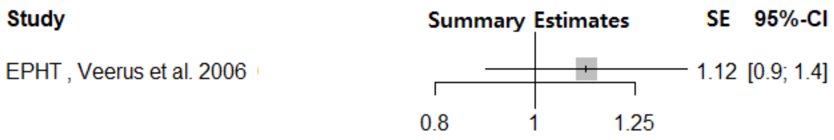

Summary estimates (95% CI) were measured by fixed-effect models if I^2 was <30% and P for heterogeneity was >0.05; otherwise, the summary estimates (95% CI) were measured by random-effect models. Forest and funnel plots were generated by "meta" package in R version 3.4.1. CHD, coronary heart disease; MHT, menopausal hormone therapy; RCTs, randomized controlled trials; SE, summary estimates.

Supplementary Figure S3.8.1. MHT and angina in RCTs: subgroup results by regimen type.

(a) estrogen only

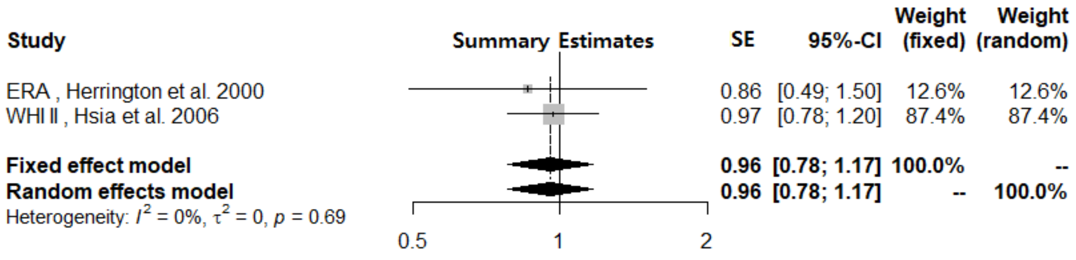

(b) combined EP

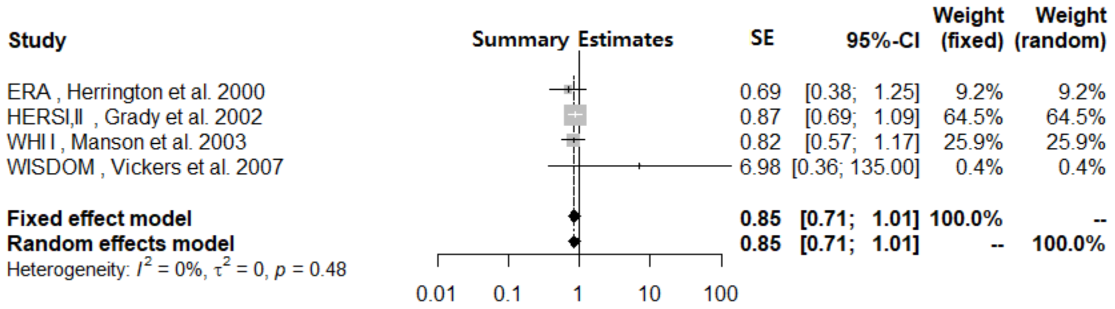

Summary estimates (95% CI) were measured by fixed-effect models if I^2 was <30% and P for heterogeneity was >0.05; otherwise, the summary estimates (95% CI) were measured by random-effect models. Forest and funnel plots were generated by "meta" package in R version 3.4.1. MHT, menopausal hormone therapy; RCTs, randomized controlled trials; SE, summary estimates.

Supplementary Figure S3.8.2. MHT and angina in RCTs: subgroup results by duration of use.

(a) duration < 5 years

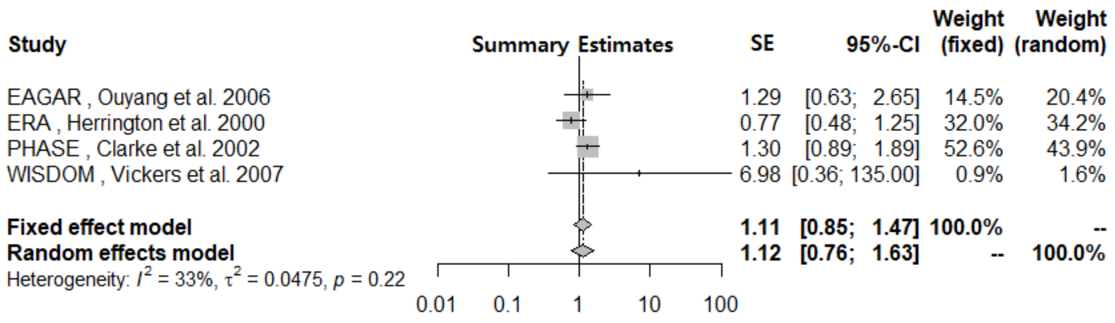

(b) duration ≥ 5 years

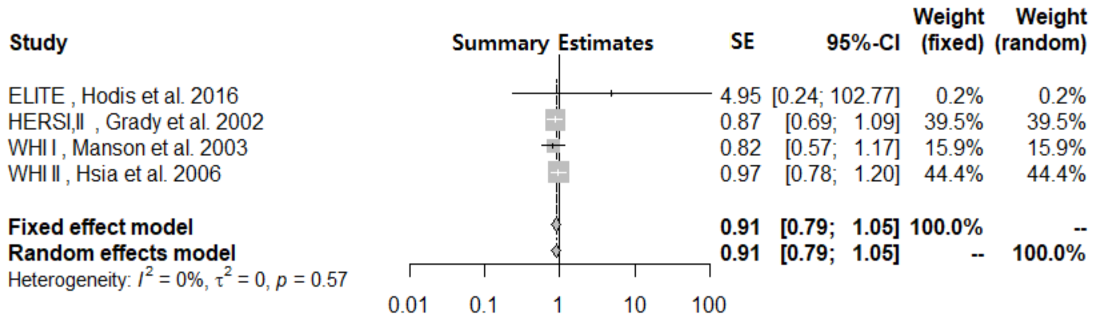

Summary estimates (95% CI) were measured by fixed-effect models if I^2 was <30% and P for heterogeneity was >0.05; otherwise, the summary estimates (95% CI) were measured by random-effect models. Forest and funnel plots were generated by "meta" package in R version 3.4.1. MHT, menopausal hormone therapy; RCTs, randomized controlled trials; SE, summary estimates.

Supplementary Figure S3.8.3. MHT and angina in RCTs: subgroup results by timing of initiation.

(a) early users

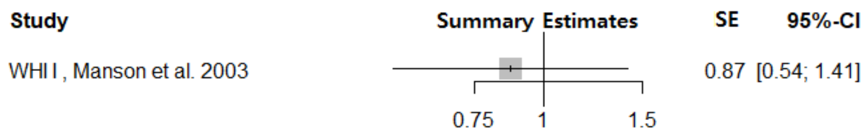

(b) late users

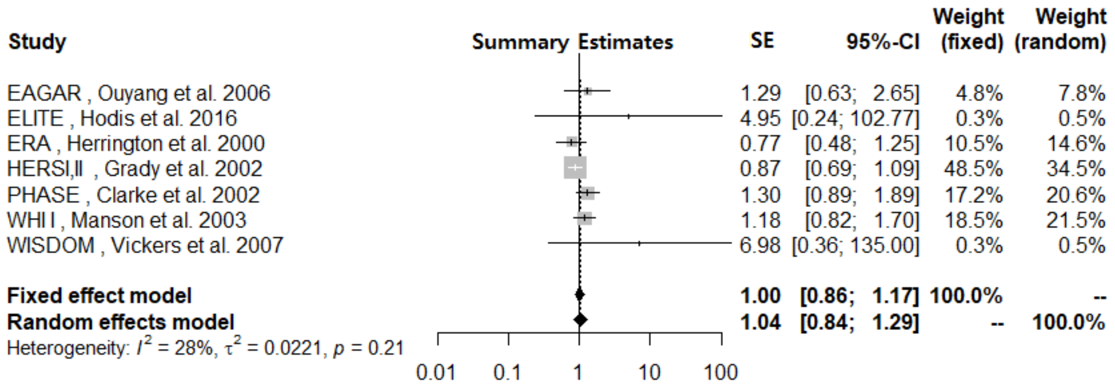

Summary estimates (95% CI) were measured by fixed-effect models if I^2 was <30% and P for heterogeneity was >0.05; otherwise, the summary estimates (95% CI) were measured by random-effect models. Forest and funnel plots were generated by "meta" package in R version 3.4.1. MHT, menopausal hormone therapy; RCTs, randomized controlled trials; SE, summary estimates.

Supplementary Figure S3.8.4. MHT and angina in RCTs: subgroup results by underlying disease.

(a) women with diseases

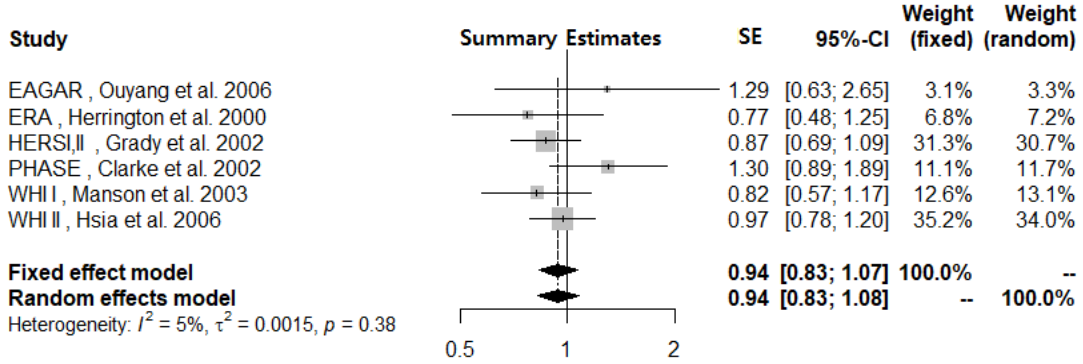

(b) women without diseases (relatively healthy)

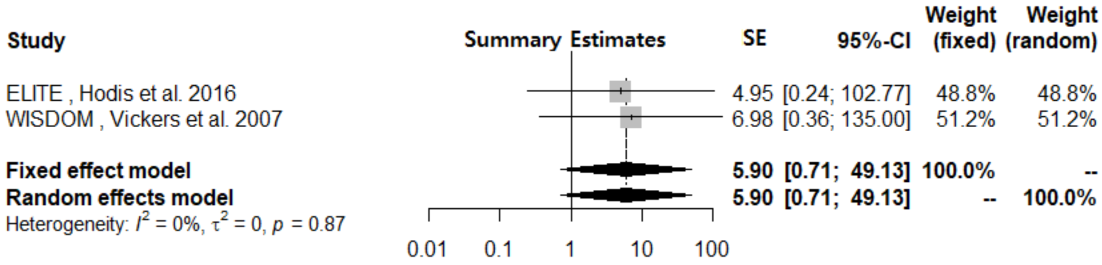

Summary estimates (95% CI) were measured by fixed-effect models if I^2 was <30% and P for heterogeneity was >0.05; otherwise, the summary estimates (95% CI) were measured by random-effect models. Forest and funnel plots were generated by "meta" package in R version 3.4.1. MHT, menopausal hormone therapy; RCTs, randomized controlled trials; SE, summary estimates.

Supplementary Figure S3.9.1. MHT and revascularization in RCTs: subgroup results by regimen type.

(a) estrogen only

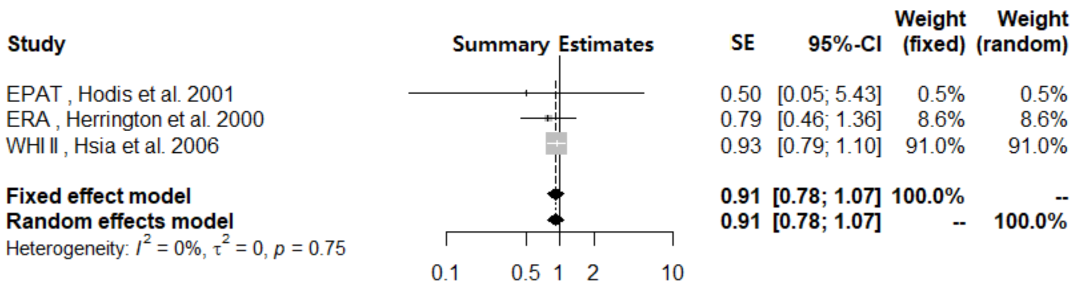

(b) combined EP

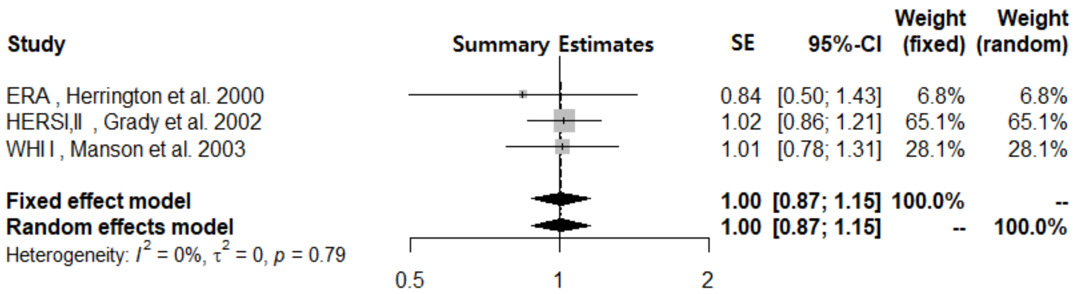

Summary estimates (95% CI) were measured by fixed-effect models if I^2 was <30% and P for heterogeneity was >0.05; otherwise, the summary estimates (95% CI) were measured by random-effect models. Forest and funnel plots were generated by "meta" package in R version 3.4.1. MHT, menopausal hormone therapy; RCTs, randomized controlled trials; SE, summary estimates.

Supplementary Figure S3.9.2. MHT and revascularization in RCTs: subgroup results by duration of use.

(a) duration < 5 years

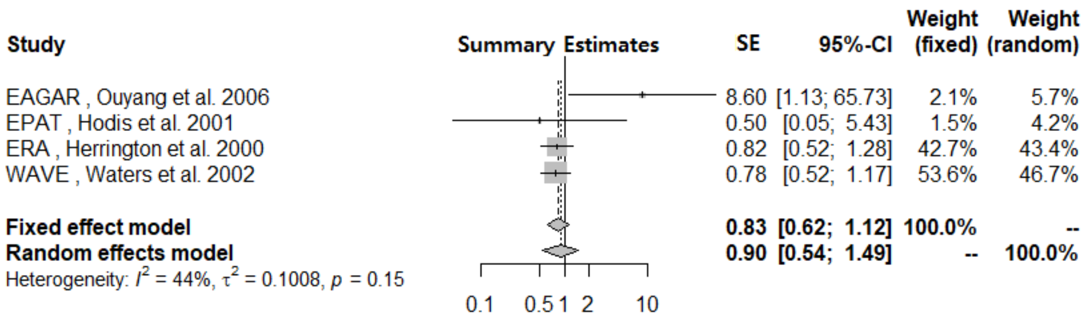

(b) duration ≥ 5 years

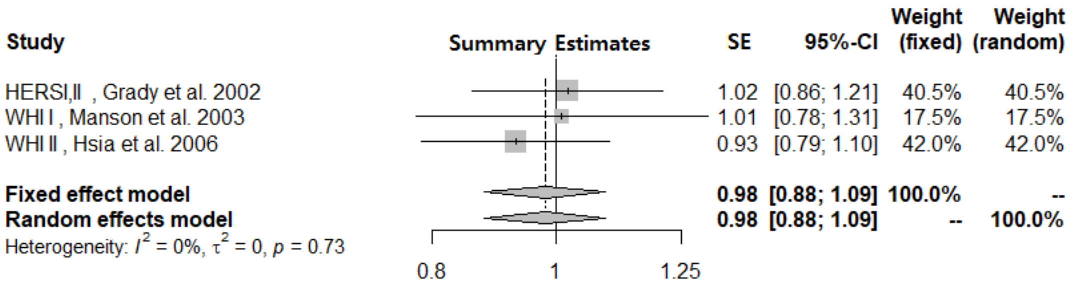

Summary estimates (95% CI) were measured by fixed-effect models if I^2 was <30% and P for heterogeneity was >0.05; otherwise, the summary estimates (95% CI) were measured by random-effect models. Forest and funnel plots were generated by "meta" package in R version 3.4.1. MHT, menopausal hormone therapy; RCTs, randomized controlled trials; SE, summary estimates.

Supplementary Figure S3.9.3. MHT and revascularization in RCTs: subgroup results by timing of initiation.

(a) early users

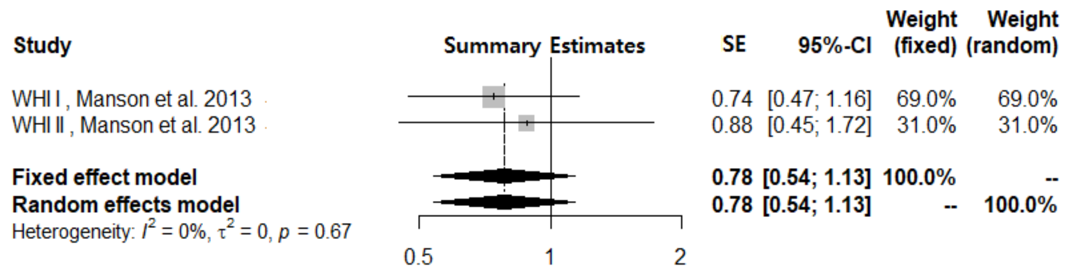

(b) late users

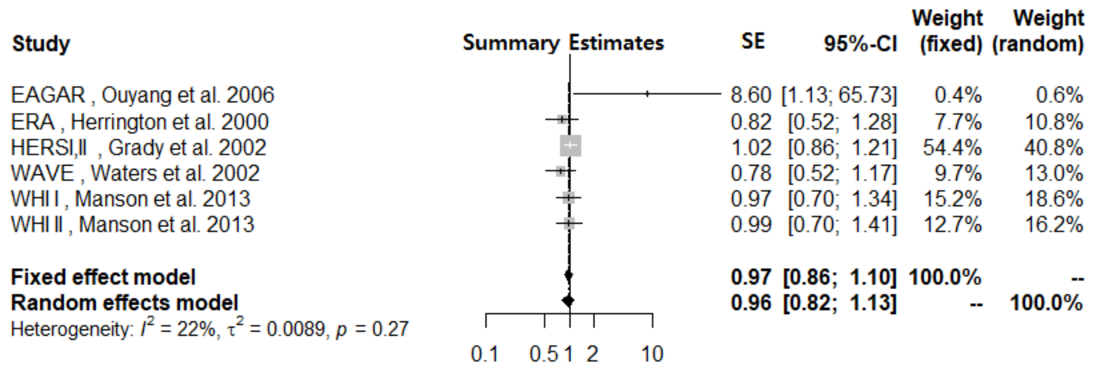

Summary estimates (95% CI) were measured by fixed-effect models if I^2 was <30% and P for heterogeneity was >0.05; otherwise, the summary estimates (95% CI) were measured by random-effect models. Forest and funnel plots were generated by "meta" package in R version 3.4.1. MHT, menopausal hormone therapy; RCTs, randomized controlled trials; SE, summary estimates.

Supplementary Figure S3.9.4. MHT and revascularization in RCTs: subgroup results by underlying disease.

(a) women with diseases

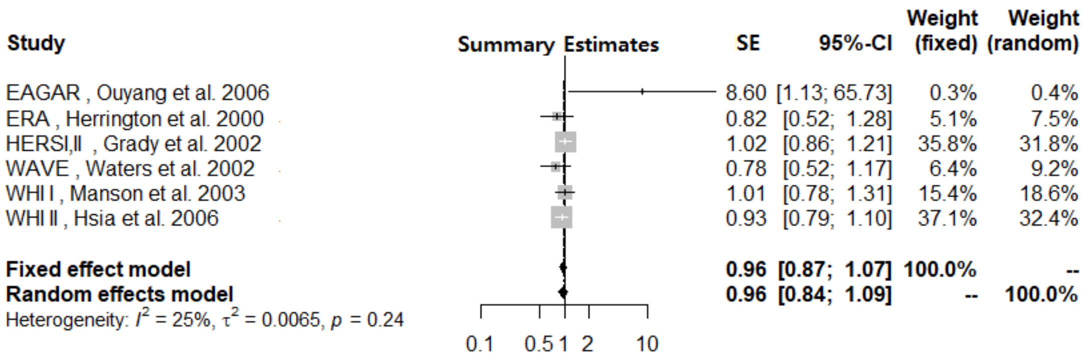

(b) women without diseases (relatively healthy)

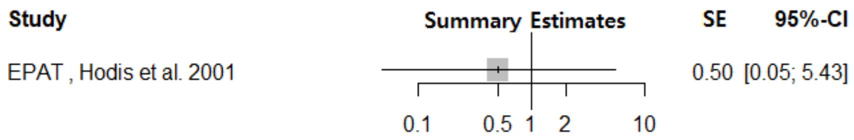

Summary estimates (95% CI) were measured by fixed-effect models if I^2 was <30% and P for heterogeneity was >0.05; otherwise, the summary estimates (95% CI) were measured by random-effect models. Forest and funnel plots were generated by "meta" package in R version 3.4.1. MHT, menopausal hormone therapy; RCTs, randomized controlled trials; SE, summary estimates.

Supplementary Figure S4.1.1. MHT and all-cause death in observational studies: subgroup results by regimen type.

(a) estrogen only

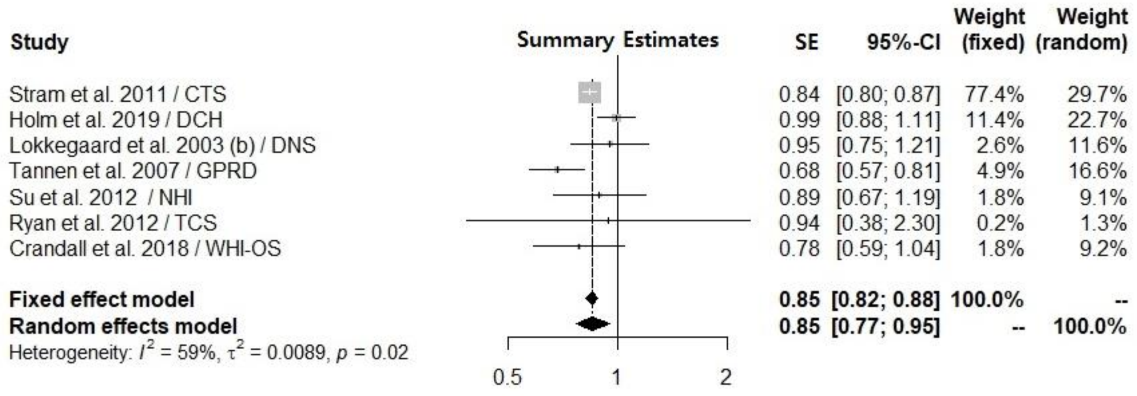

(b) combined EP

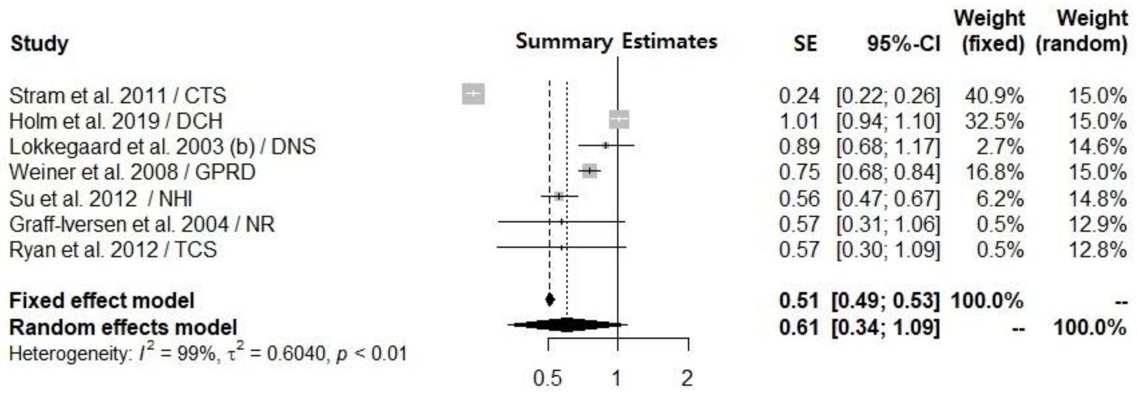

Summary estimates (95% CI) were measured by fixed-effect models if I^2 was <30% and P for heterogeneity was >0.05; otherwise, the summary estimates (95% CI) were measured by random-effect models. Forest and funnel plots were generated by "meta" package in R version 3.4.1. MHT, menopausal hormone therapy; SE, summary estimates.

Supplementary Figure S4.1.2. MHT and all-cause death in observational studies: subgroup results by duration of use.

(a) duration < 5 years

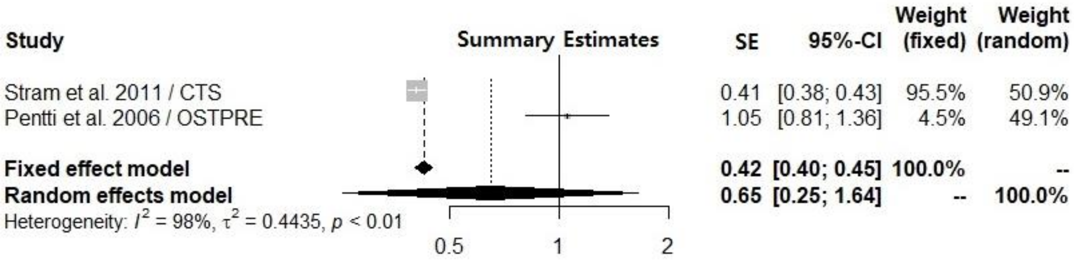

(b) duration ≥ 5 years

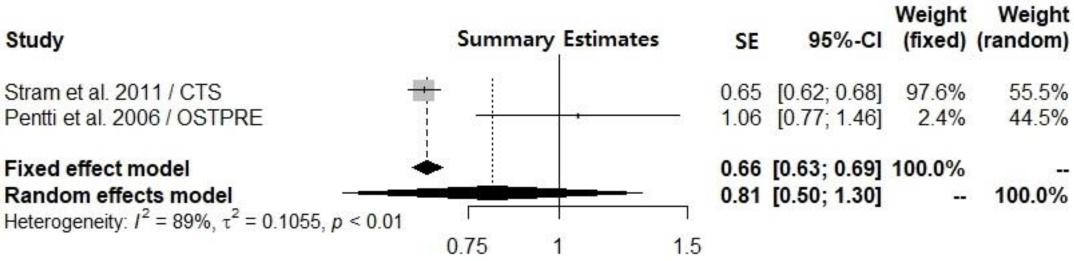

Summary estimates (95% CI) were measured by fixed-effect models if I^2 was <30% and P for heterogeneity was >0.05; otherwise, the summary estimates (95% CI) were measured by random-effect models. Forest and funnel plots were generated by "meta" package in R version 3.4.1. MHT, menopausal hormone therapy; SE, summary estimates.

Supplementary Figure S4.1.3. MHT and all-cause death in observational studies: subgroup results by timing of initiation.

(a) early users

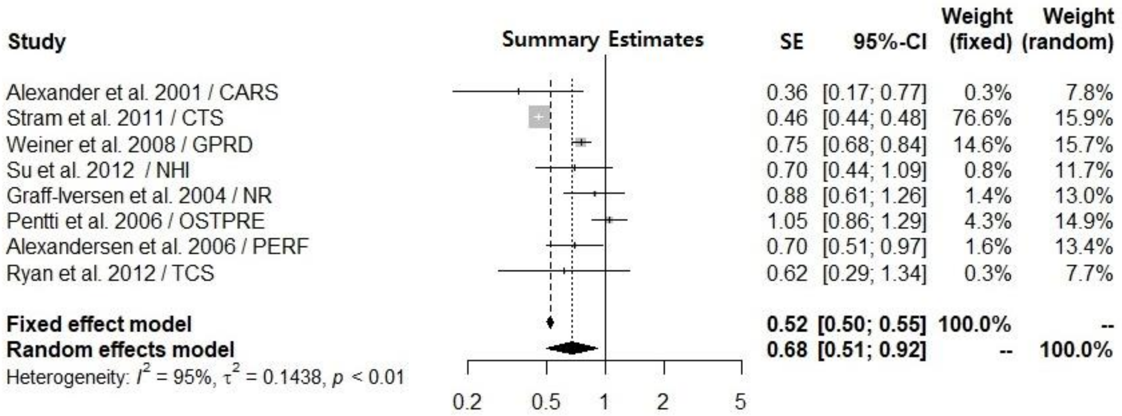

(b) late users

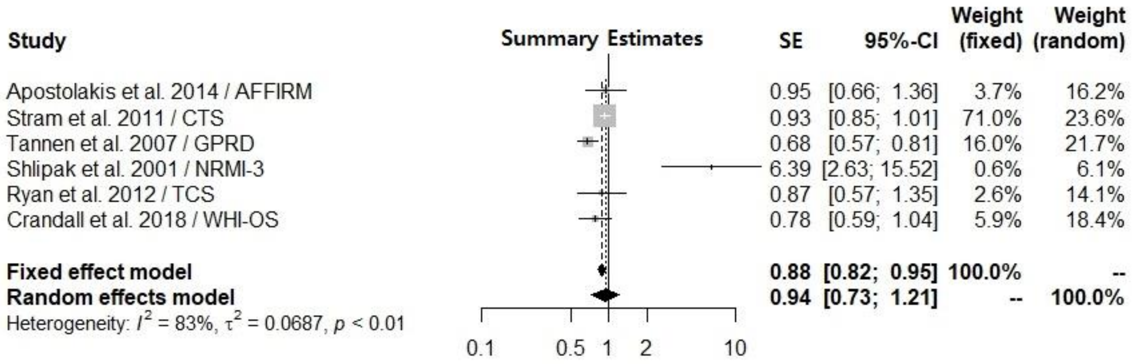

Summary estimates (95% CI) were measured by fixed-effect models if I^2 was <30% and P for heterogeneity was >0.05; otherwise, the summary estimates (95% CI) were measured by random-effect models. Forest and funnel plots were generated by "meta" package in R version 3.4.1. MHT, menopausal hormone therapy; SE, summary estimates.

Supplementary Figure S4.1.4. MHT and all-cause death in observational studies: subgroup results by route of administration.

(a) oral

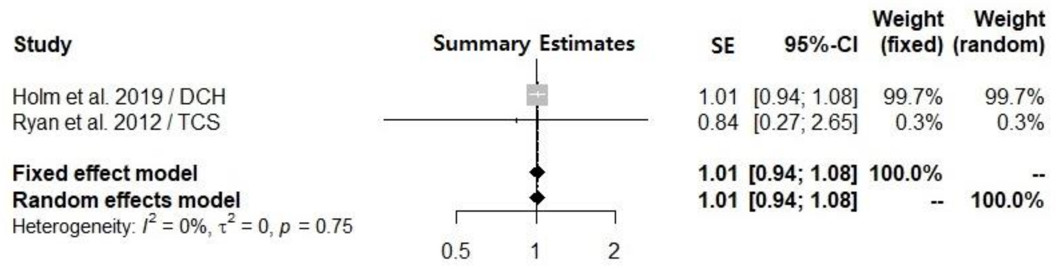

(a) non-oral

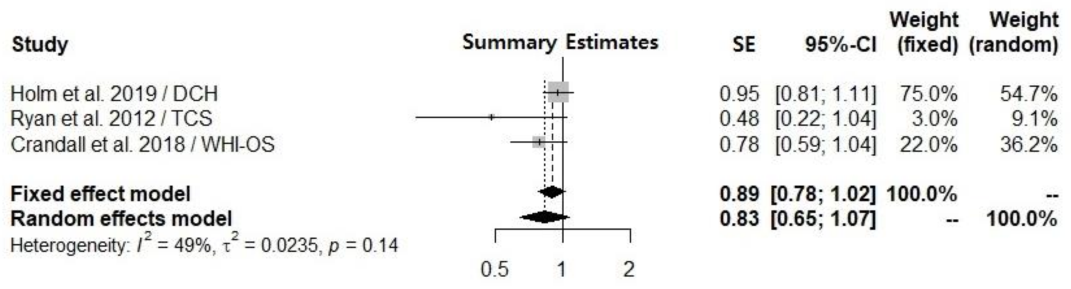

Summary estimates (95% CI) were measured by fixed-effect models if I^2 was <30% and P for heterogeneity was >0.05; otherwise, the summary estimates (95% CI) were measured by random-effect models. Forest and funnel plots were generated by "meta" package in R version 3.4.1. MHT, menopausal hormone therapy; SE, summary estimates.

Supplementary Figure S4.1.5. MHT and all-cause death in observational studies: subgroup results by underlying disease.

(a) women with diseases

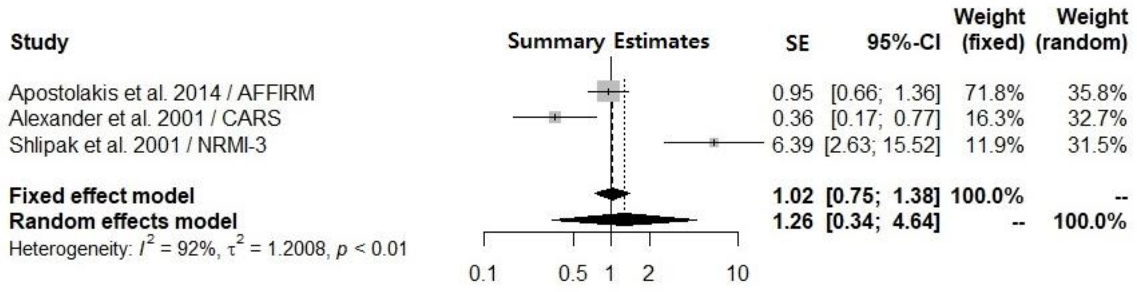

(b) women without diseases (relatively healthy)

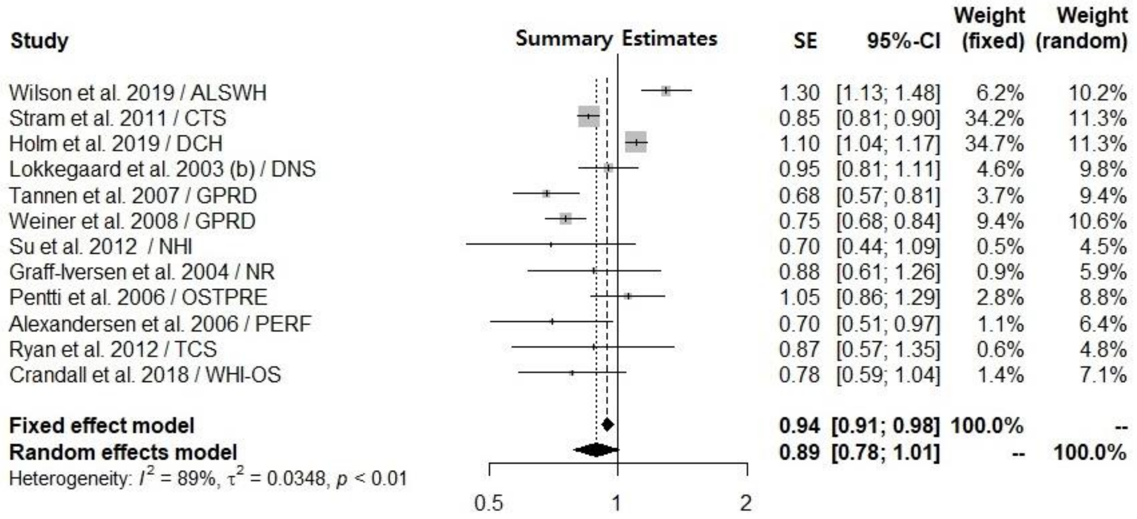

Summary estimates (95% CI) were measured by fixed-effect models if I^2 was <30% and P for heterogeneity was >0.05; otherwise, the summary estimates (95% CI) were measured by random-effect models. Forest and funnel plots were generated by "meta" package in R version 3.4.1. MHT, menopausal hormone therapy; SE, summary estimates.

Supplementary Figure S4.1.6. MHT and all-cause death in observational studies: subgroup results by recency of MHT.

(a) past

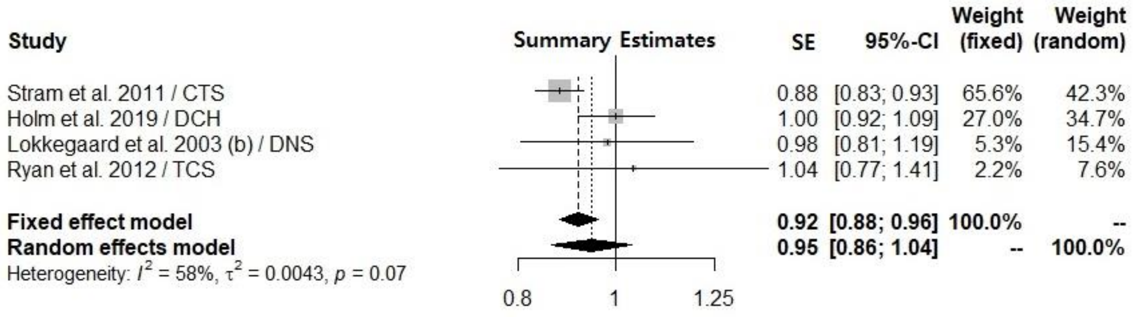

(b) current

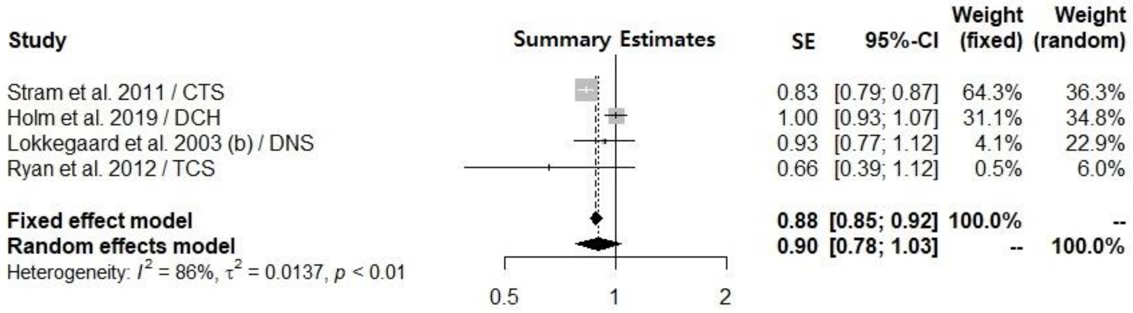

Summary estimates (95% CI) were measured by fixed-effect models if I^2 was <30% and P for heterogeneity was >0.05; otherwise, the summary estimates (95% CI) were measured by random-effect models. Forest and funnel plots were generated by "meta" package in R version 3.4.1. MHT, menopausal hormone therapy; SE, summary estimates.

Supplementary Figure S4.1.7. MHT and all-cause death in observational studies: subgroup results by study design.

(a) cohort

Study	SE	95%-CI	Weight (fixed)	Weight (random)
Apostolakis et al. 2014 / AFFIRM	0.95	[0.66; 1.36]	0.8%	5.5%
Wilson et al. 2019 / ALSWH	1.30	[1.13; 1.48]	6.1%	9.0%
Alexander et al. 2001 / CARS	0.36	[0.17; 0.77]	0.2%	2.2%
Stram et al. 2011 / CTS	0.85	[0.81; 0.90]	33.8%	9.8%
Holm et al. 2019 / DCH	1.10	[1.04; 1.17]	34.3%	9.8%
Lokkegaard et al. 2003 (b) / DNS	0.95	[0.81; 1.11]	4.6%	8.7%
Tannen et al. 2007 / GPRD	0.68	[0.57; 0.81]	3.6%	8.4%
Weiner et al. 2008 / GPRD	0.75	[0.68; 0.84]	9.3%	9.3%
Su et al. 2012 / NHI	0.70	[0.44; 1.09]	0.5%	4.4%
Graff-Iversen et al. 2004 / NR	0.88	[0.61; 1.26]	0.9%	5.6%
Shlipak et al. 2001 / NRMI-3	6.39	[2.63; 15.52]	0.1%	1.7%
Pentti et al. 2006 / OSTPRE	1.05	[0.86; 1.29]	2.7%	8.0%
Alexandersen et al. 2006 / PERF	0.70	[0.51; 0.97]	1.0%	6.1%
Ryan et al. 2012 / TCS	0.87	[0.57; 1.35]	0.6%	4.6%
Crandall et al. 2018 / WHI-OS	0.78	[0.59; 1.04]	1.3%	6.6%
**Fixed effect model**	**0.95**	**[0.91; 0.98]**	**100.0%**	**--**
**Random effects model**	**0.90**	**[0.79; 1.02]**	**--**	**100.0%**

Heterogeneity: $I^2 = 89\%$, $\tau^2 = 0.0422$, $p < 0.01$

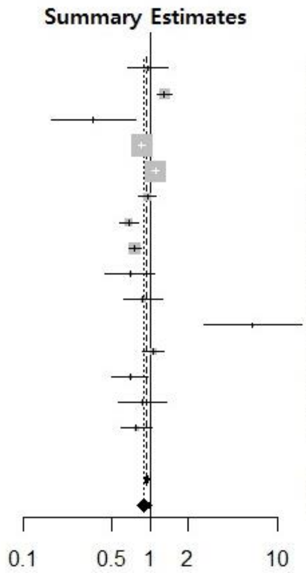

Summary estimates (95% CI) were measured by fixed-effect models if I^2 was <30% and P for heterogeneity was >0.05; otherwise, the summary estimates (95% CI) were measured by random-effect models. Forest and funnel plots were generated by "meta" package in R version 3.4.1. MHT, menopausal hormone therapy; SE, summary estimates.

Supplementary Figure S4.1.8. MHT and all-cause death in observational studies: subgroup results by study quality.

(a) good and fair

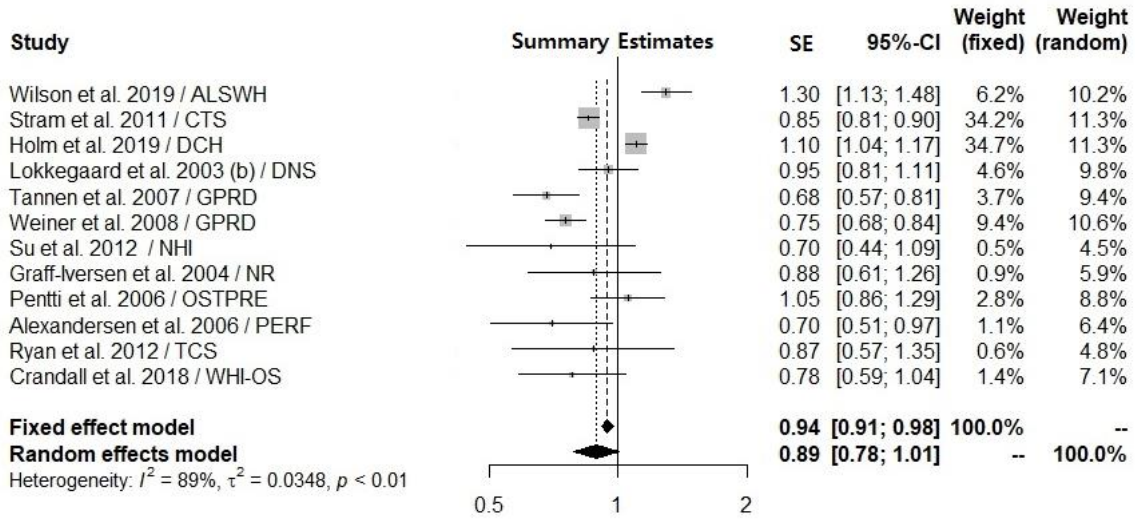

(b) poor

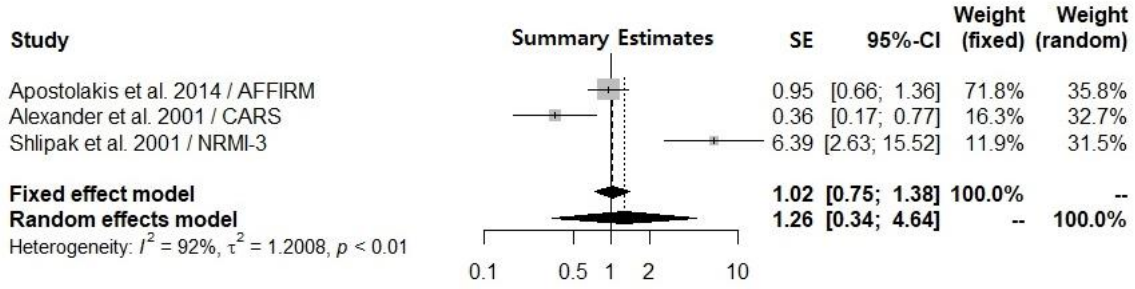

Summary estimates (95% CI) were measured by fixed-effect models if I^2 was <30% and P for heterogeneity was >0.05; otherwise, the summary estimates (95% CI) were measured by random-effect models. Forest and funnel plots were generated by "meta" package in R version 3.4.1. MHT, menopausal hormone therapy; SE, summary estimates.

Supplementary Figure S4.2.1. MHT and stroke in observational studies: subgroup results by regimen type.

(a) estrogen only

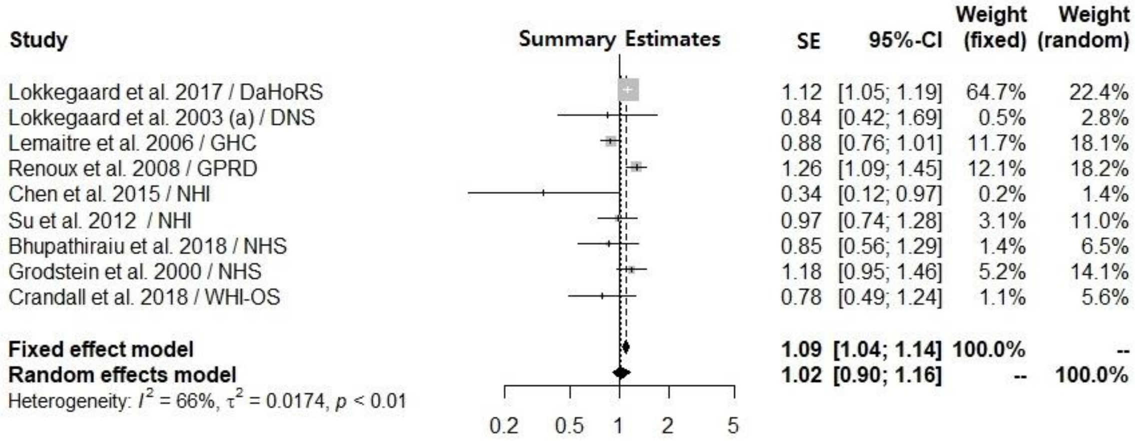

(b) combined EP

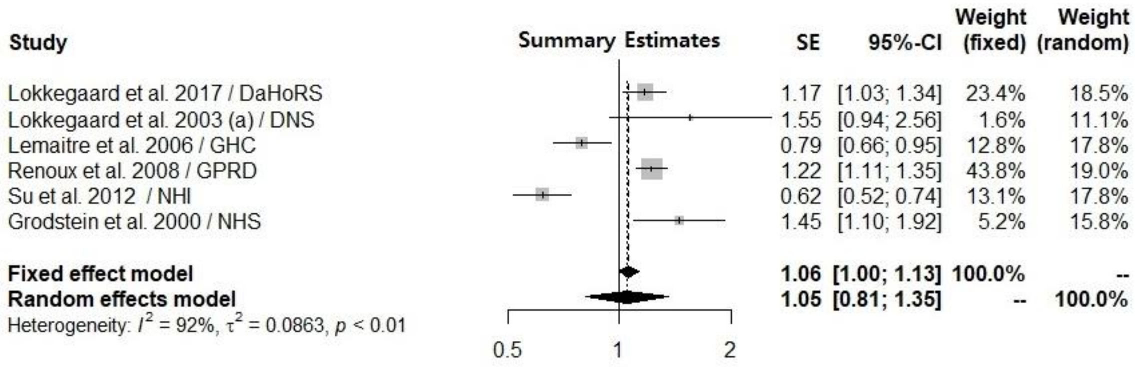

Summary estimates (95% CI) were measured by fixed-effect models if I^2 was <30% and P for heterogeneity was >0.05; otherwise, the summary estimates (95% CI) were measured by random-effect models. Forest and funnel plots were generated by "meta" package in R version 3.4.1. MHT, menopausal hormone therapy; SE, summary estimates.

Supplementary Figure S4.2.2. MHT and stroke in observational studies: subgroup results by duration of use.

(a) duration < 5 years

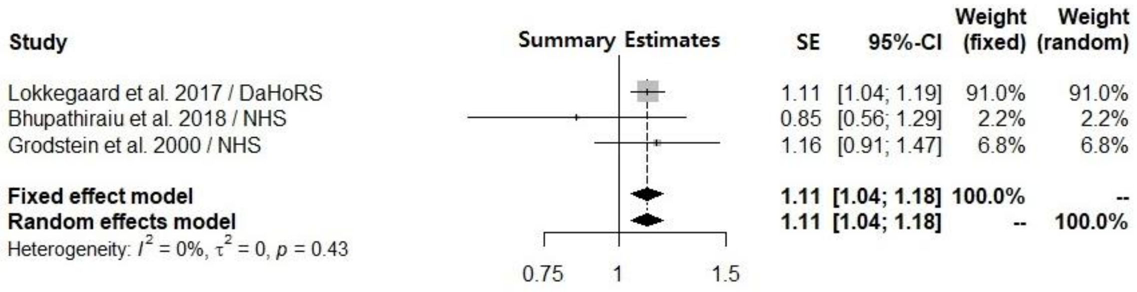

(b) duration ≥ 5 years

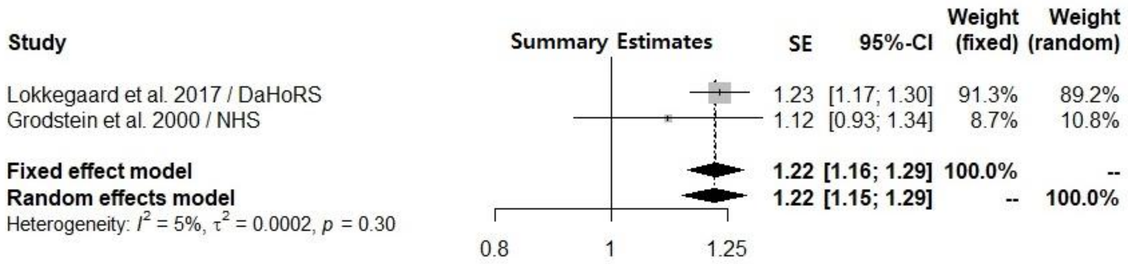

Summary estimates (95% CI) were measured by fixed-effect models if I^2 was <30% and P for heterogeneity was >0.05; otherwise, the summary estimates (95% CI) were measured by random-effect models. Forest and funnel plots were generated by "meta" package in R version 3.4.1. MHT, menopausal hormone therapy; SE, summary estimates.

Supplementary Figure S4.2.3. MHT and stroke in observational studies: subgroup results by timing of initiation.

(a) early users

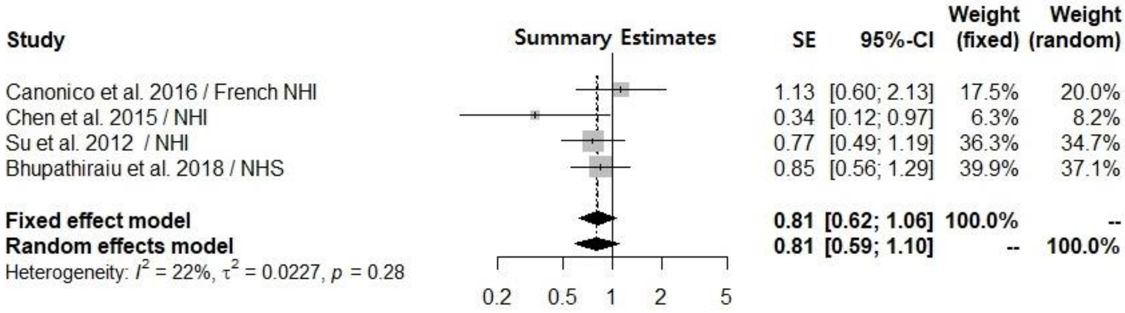

(b) late users

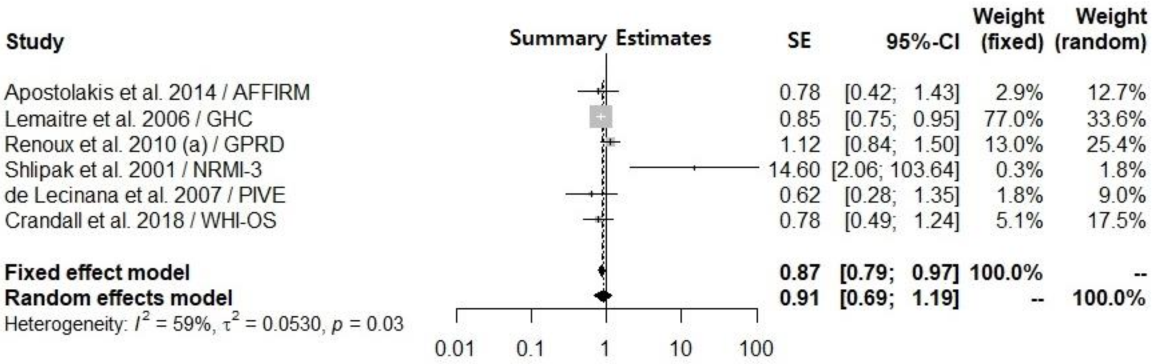

Summary estimates (95% CI) were measured by fixed-effect models if I^2 was <30% and P for heterogeneity was >0.05; otherwise, the summary estimates (95% CI) were measured by random-effect models. Forest and funnel plots were generated by "meta" package in R version 3.4.1. MHT, menopausal hormone therapy; SE, summary estimates.

Supplementary Figure S4.2.4. MHT and stroke in observational studies: subgroup results by route of administration.

(a) oral

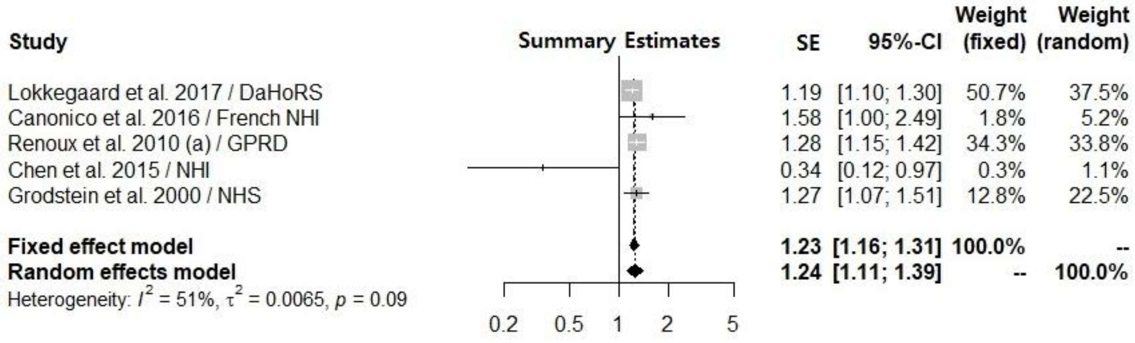

(b) non-oral

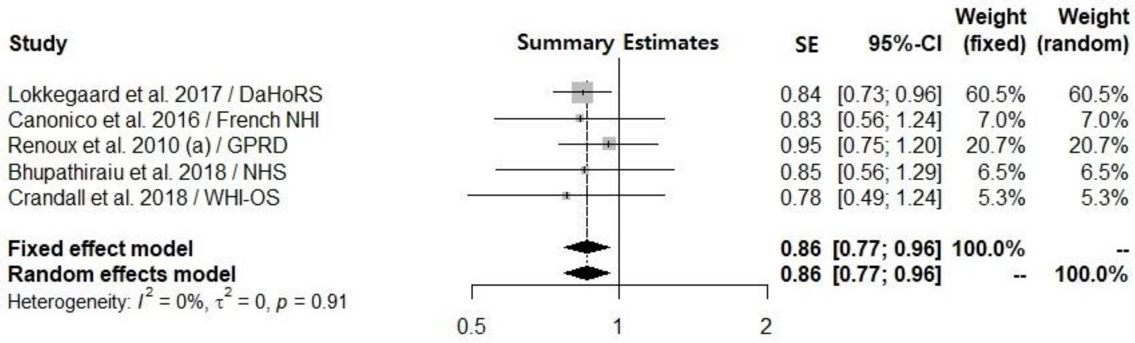

Summary estimates (95% CI) were measured by fixed-effect models if I^2 was <30% and P for heterogeneity was >0.05; otherwise, the summary estimates (95% CI) were measured by random-effect models. Forest and funnel plots were generated by "meta" package in R version 3.4.1. MHT, menopausal hormone therapy; SE, summary estimates.

Supplementary Figure S4.2.5. MHT and stroke in observational studies: subgroup results by underlying disease.

(a) women with diseases

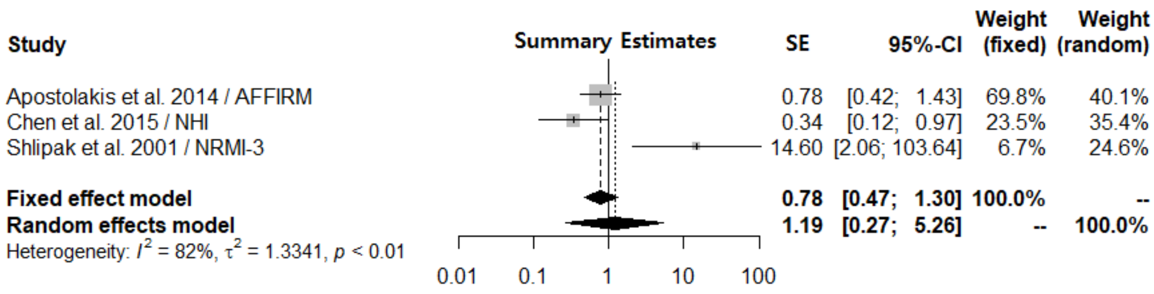

(b) women without diseases (relatively healthy)

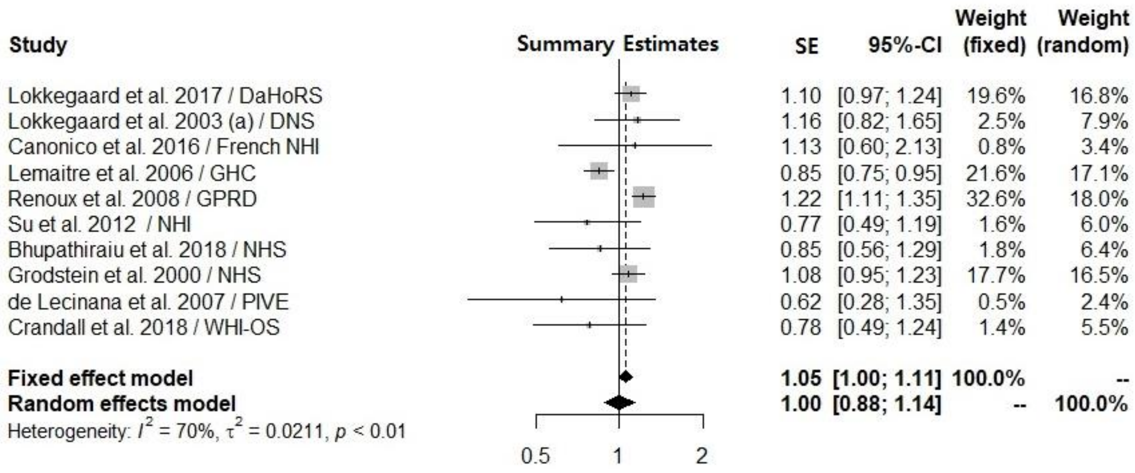

Summary estimates (95% CI) were measured by fixed-effect models if I^2 was <30% and P for heterogeneity was >0.05; otherwise, the summary estimates (95% CI) were measured by random-effect models. Forest and funnel plots were generated by "meta" package in R version 3.4.1. MHT, menopausal hormone therapy; SE, summary estimates.

Supplementary Figure S4.2.6. MHT and stroke in observational studies: subgroup results by recency of MHT.

(a) past

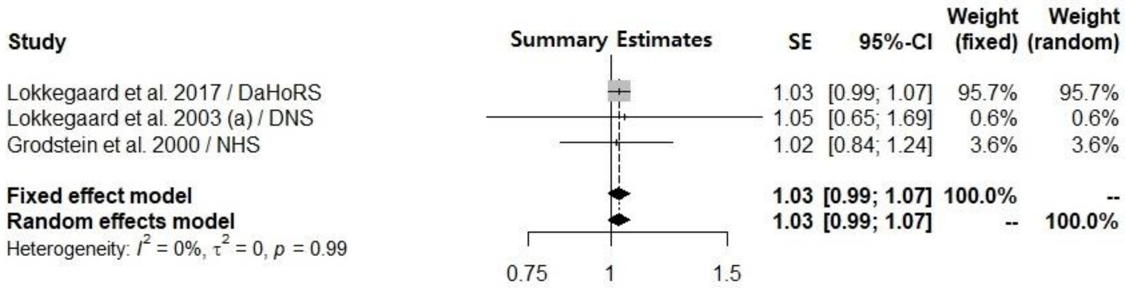

(b) current

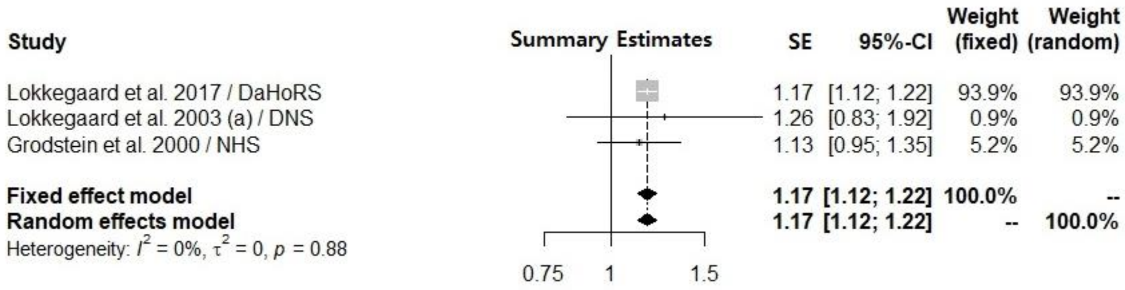

Summary estimates (95% CI) were measured by fixed-effect models if I^2 was <30% and P for heterogeneity was >0.05; otherwise, the summary estimates (95% CI) were measured by random-effect models. Forest and funnel plots were generated by "meta" package in R version 3.4.1. MHT, menopausal hormone therapy; SE, summary estimates.

Supplementary Figure S4.2.7. MHT and stroke in observational studies: subgroup results by study design.

(a) cohort

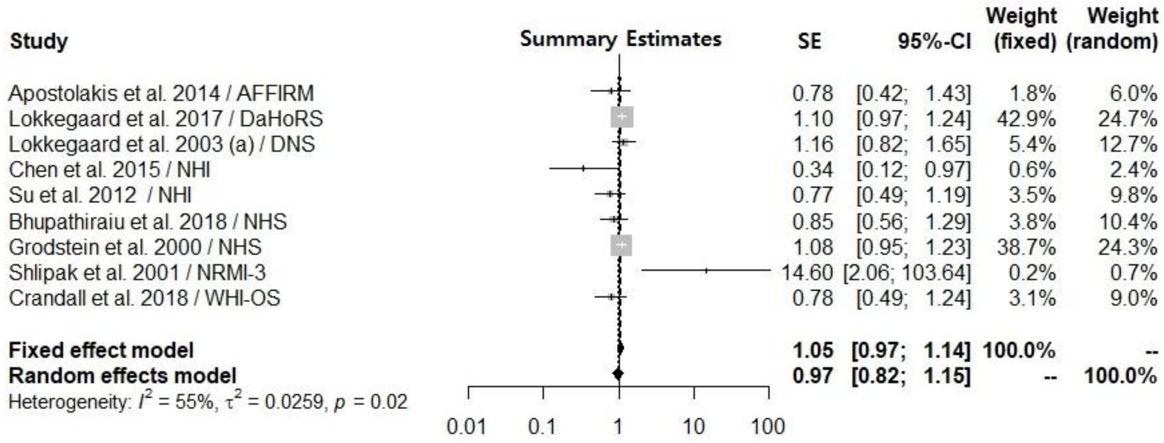

(b) case-control study

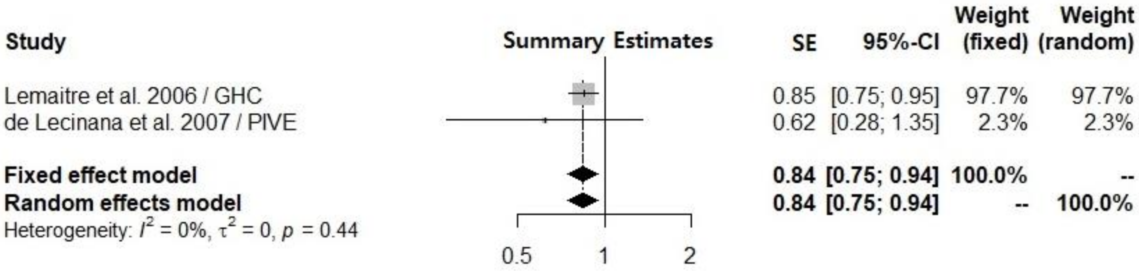

(c) nested case-control study

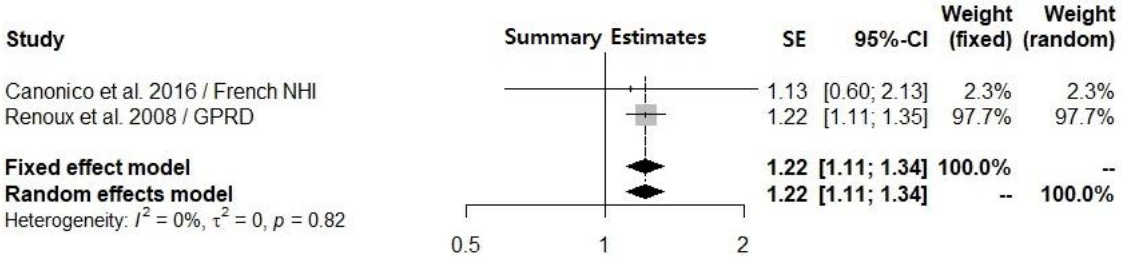

Summary estimates (95% CI) were measured by fixed-effect models if I^2 was <30% and P for heterogeneity was >0.05; otherwise, the summary estimates (95% CI) were measured by random-effect models. Forest and funnel plots were generated by "meta" package in R version 3.4.1. MHT, menopausal hormone therapy; SE, summary estimates.

Supplementary Figure S4.2.8. MHT and stroke in observational studies: subgroup results by study quality.

(a) good and fair

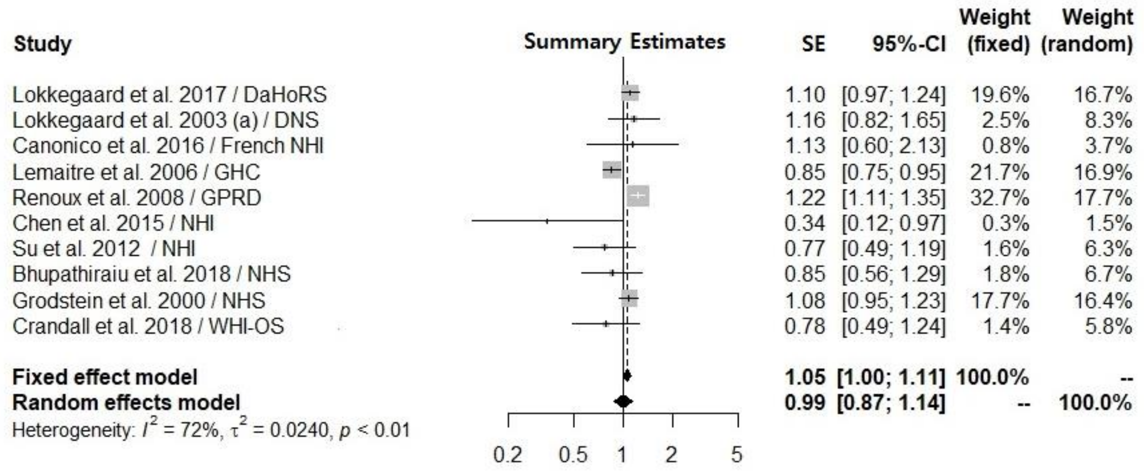

(b) poor

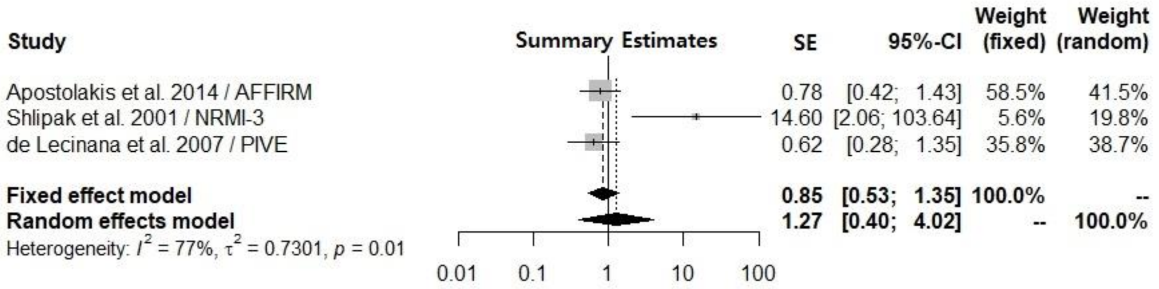

Summary estimates (95% CI) were measured by fixed-effect models if I^2 was <30% and P for heterogeneity was >0.05; otherwise, the summary estimates (95% CI) were measured by random-effect models. Forest and funnel plots were generated by "meta" package in R version 3.4.1. MHT, menopausal hormone therapy; SE, summary estimates.

Supplementary Figure S4.3.1. MHT and VTE in observational studies: subgroup results by regimen type.

(a) estrogen only

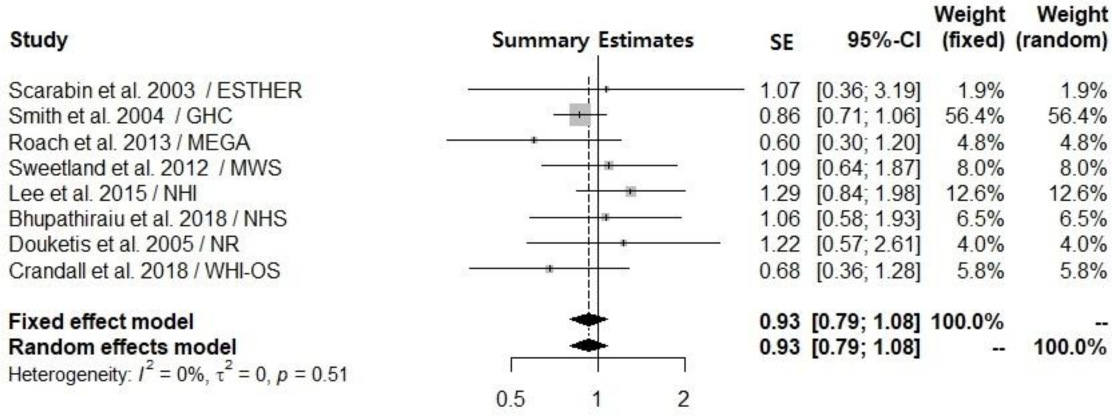

(b) combined EP

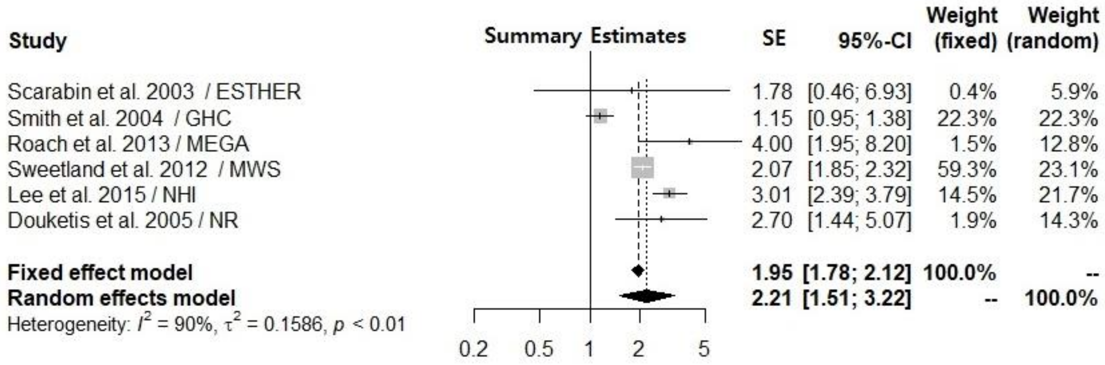

Summary estimates (95% CI) were measured by fixed-effect models if I^2 was <30% and P for heterogeneity was >0.05; otherwise, the summary estimates (95% CI) were measured by random-effect models. Forest and funnel plots were generated by "meta" package in R version 3.4.1. MHT, menopausal hormone therapy; SE, summary estimates; VTE, venous thromboembolism.

Supplementary Figure S4.3.2. MHT and VTE in observational studies: subgroup results by duration of use.

(a) duration < 5 years

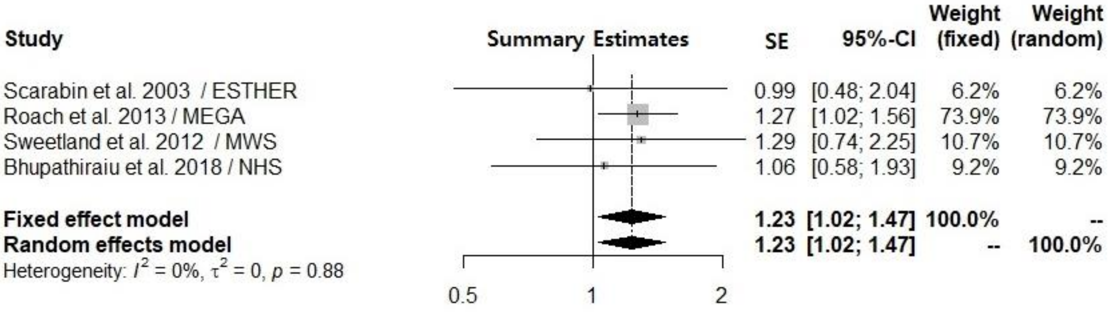

(b) duration ≥ 5 years

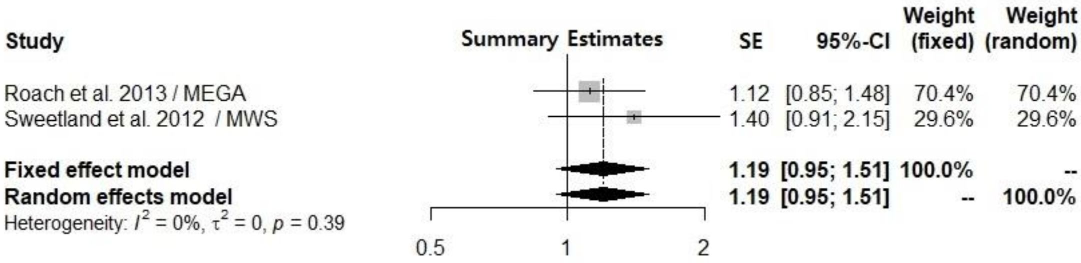

Summary estimates (95% CI) were measured by fixed-effect models if I^2 was <30% and P for heterogeneity was >0.05; otherwise, the summary estimates (95% CI) were measured by random-effect models. Forest and funnel plots were generated by "meta" package in R version 3.4.1. MHT, menopausal hormone therapy; SE, summary estimates; VTE, venous thromboembolism.

Supplementary Figure S4.3.3. MHT and VTE in observational studies: subgroup results by timing of initiation.

(a) early users

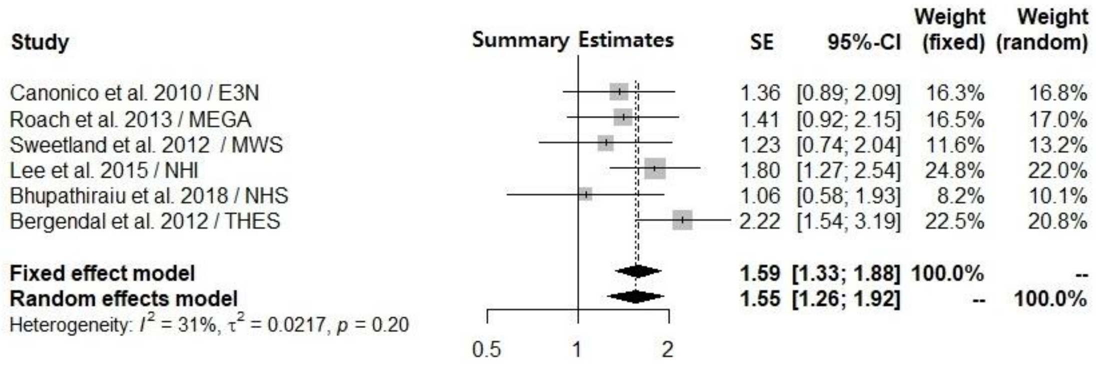

(b) late users

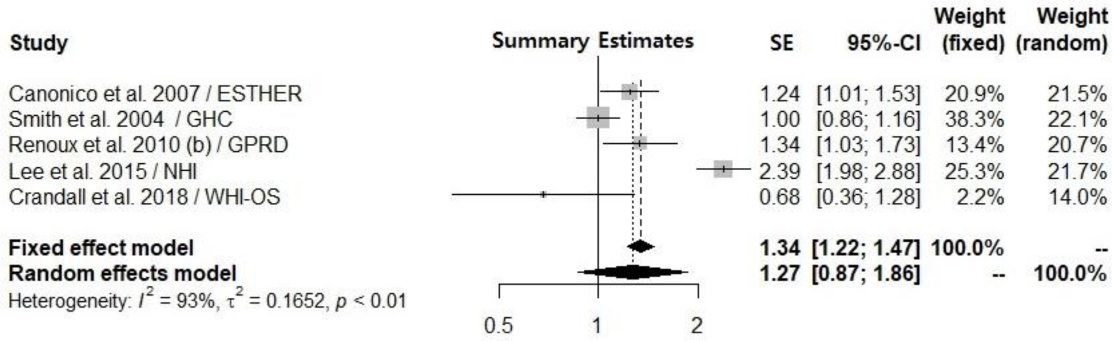

Summary estimates (95% CI) were measured by fixed-effect models if I^2 was <30% and P for heterogeneity was >0.05; otherwise, the summary estimates (95% CI) were measured by random-effect models. Forest and funnel plots were generated by "meta" package in R version 3.4.1. MHT, menopausal hormone therapy; SE, summary estimates; VTE, venous thromboembolism.

Supplementary Figure S4.3.4. MHT and VTE in observational studies: subgroup results by route of administration.

(a) oral

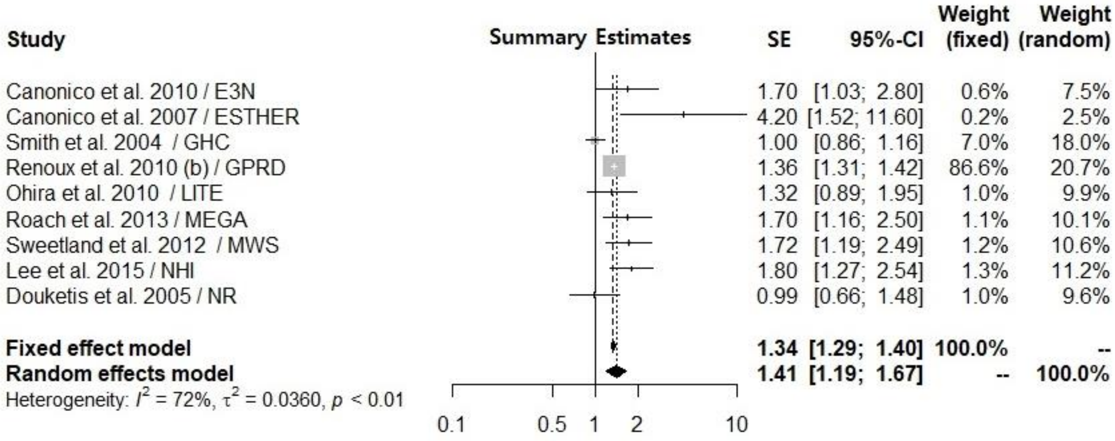

(b) non-oral

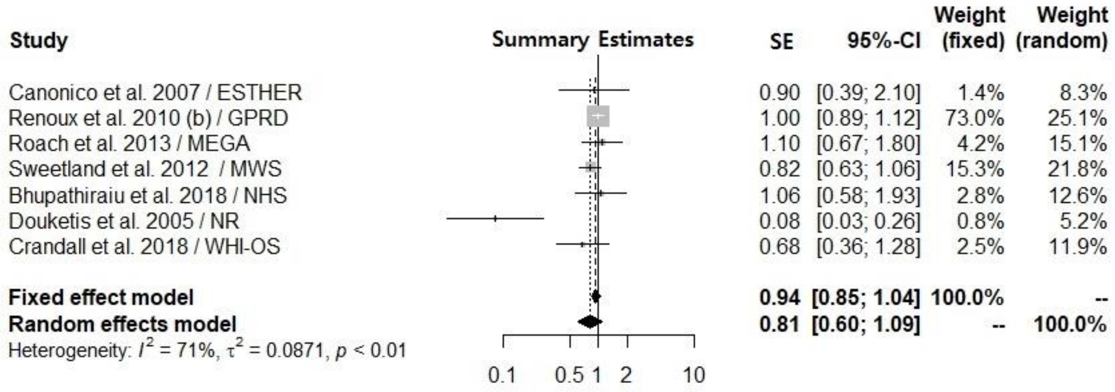

Summary estimates (95% CI) were measured by fixed-effect models if I^2 was <30% and P for heterogeneity was >0.05; otherwise, the summary estimates (95% CI) were measured by random-effect models. Forest and funnel plots were generated by "meta" package in R version 3.4.1. MHT, menopausal hormone therapy; SE, summary estimates; VTE, venous thromboembolism.

Supplementary Figure S4.3.5. MHT and VTE in observational studies: subgroup results by underlying disease.

(a) women without diseases (relatively healthy)

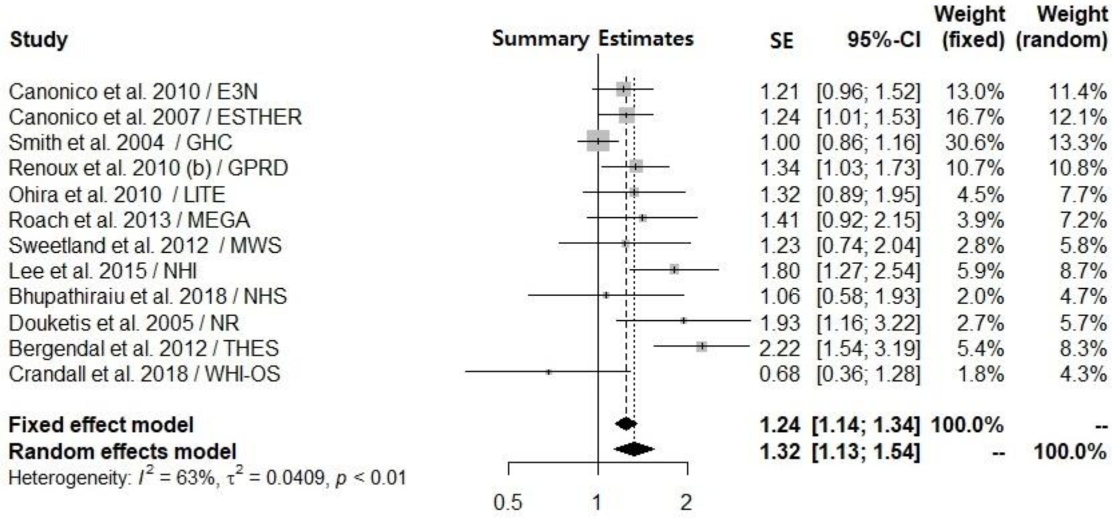

Summary estimates (95% CI) were measured by fixed-effect models if I^2 was <30% and P for heterogeneity was >0.05; otherwise, the summary estimates (95% CI) were measured by random-effect models. Forest and funnel plots were generated by "meta" package in R version 3.4.1. MHT, menopausal hormone therapy; SE, summary estimates; VTE, venous thromboembolism.

Supplementary Figure S4.3.6. MHT and VTE in observational studies: subgroup results by recency of MHT.

(a) past

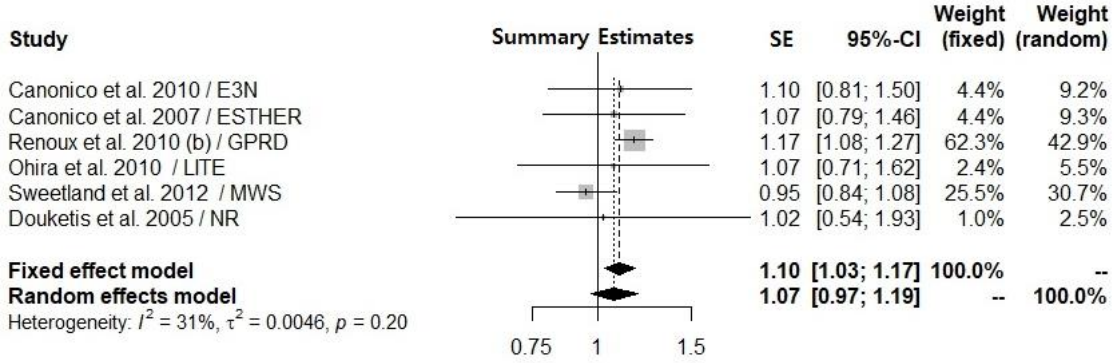

(b) current

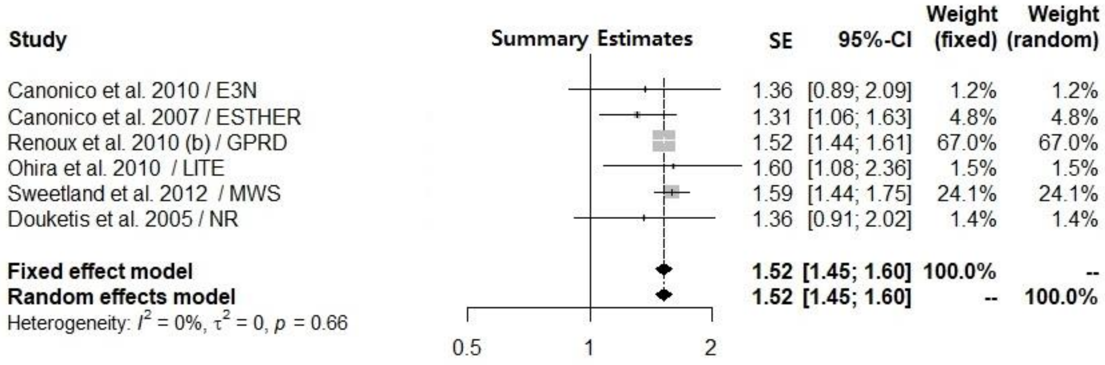

Summary estimates (95% CI) were measured by fixed-effect models if I^2 was <30% and P for heterogeneity was >0.05; otherwise, the summary estimates (95% CI) were measured by random-effect models. Forest and funnel plots were generated by "meta" package in R version 3.4.1. MHT, menopausal hormone therapy; SE, summary estimates; VTE, venous thromboembolism.

Supplementary Figure S4.3.7. MHT and VTE in observational studies: subgroup results by study design.

(a) cohort

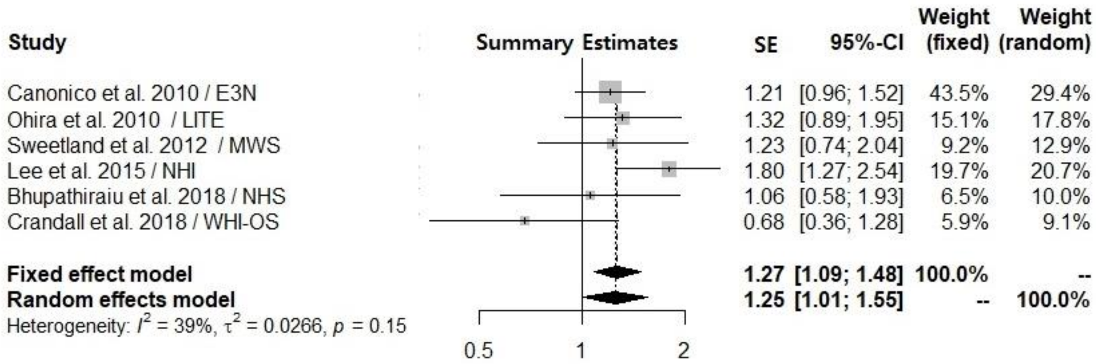

(b) case-control study

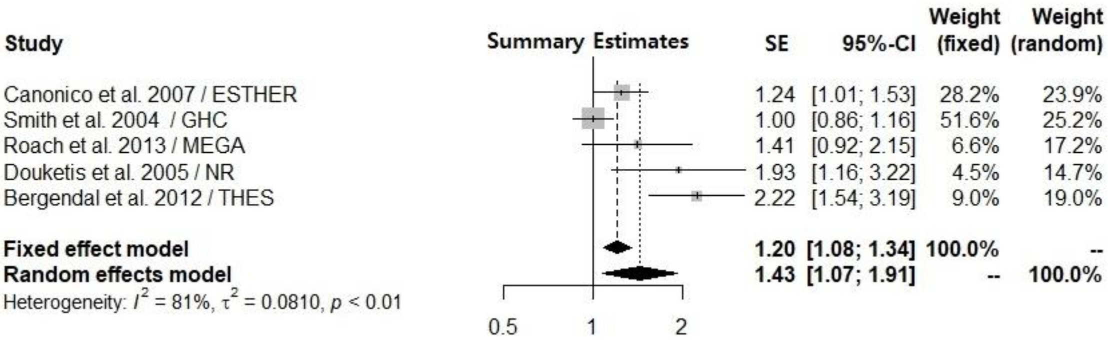

(b) nested case-control study

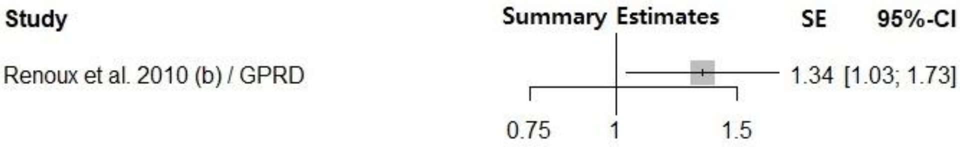

Summary estimates (95% CI) were measured by fixed-effect models if I^2 was <30% and P for heterogeneity was >0.05; otherwise, the summary estimates (95% CI) were measured by random-effect models. Forest and funnel plots were generated by "meta" package in R version 3.4.1. MHT, menopausal hormone therapy; SE, summary estimates; VTE, venous thromboembolism.

Supplementary Figure S4.3.8. MHT and VTE in observational studies: subgroup results by study quality.

(a) good and fair

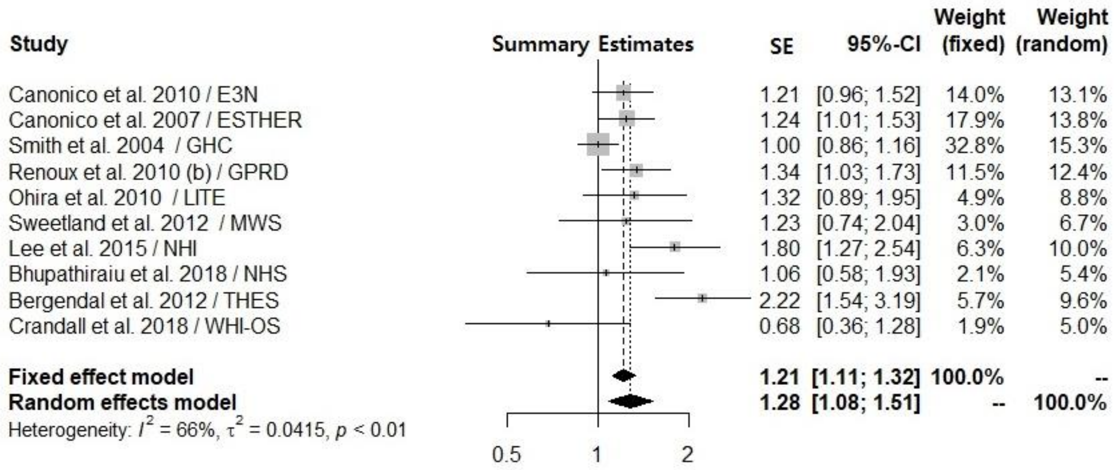

(b) poor

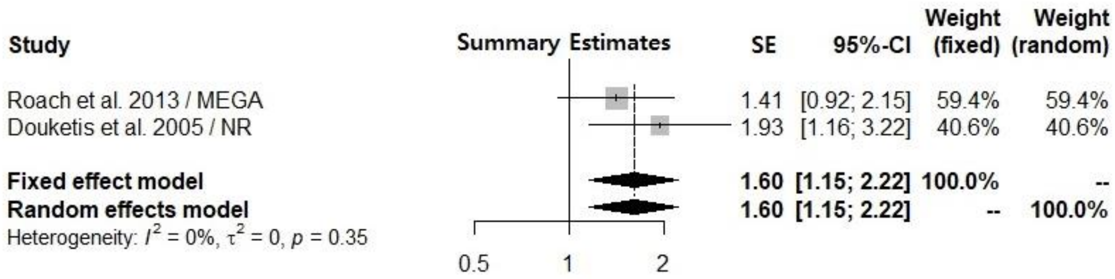

Summary estimates (95% CI) were measured by fixed-effect models if I^2 was <30% and P for heterogeneity was >0.05; otherwise, the summary estimates (95% CI) were measured by random-effect models. Forest and funnel plots were generated by "meta" package in R version 3.4.1. MHT, menopausal hormone therapy; SE, summary estimates; VTE, venous thromboembolism.

Supplementary Figure S4.4.1. MHT and MI in observational studies: subgroup results by regimen type.

(a) estrogen only

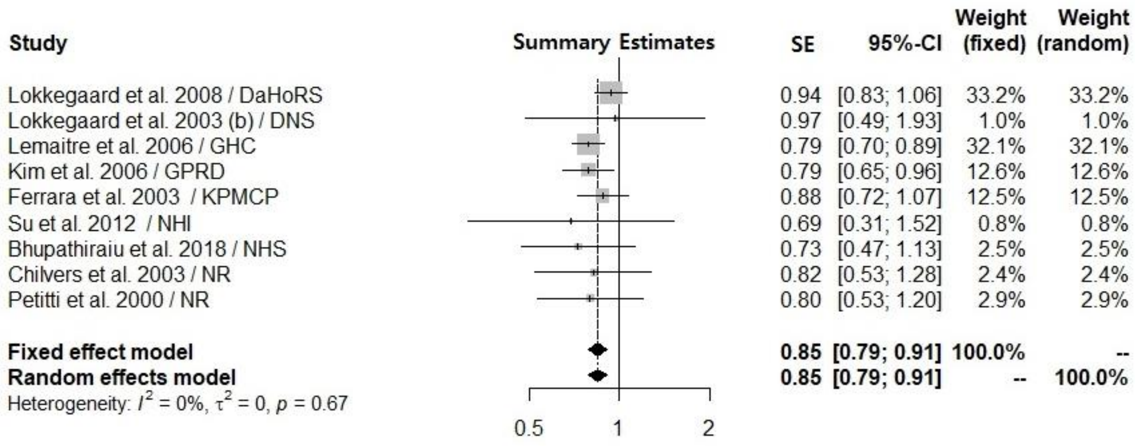

(b) combined EP

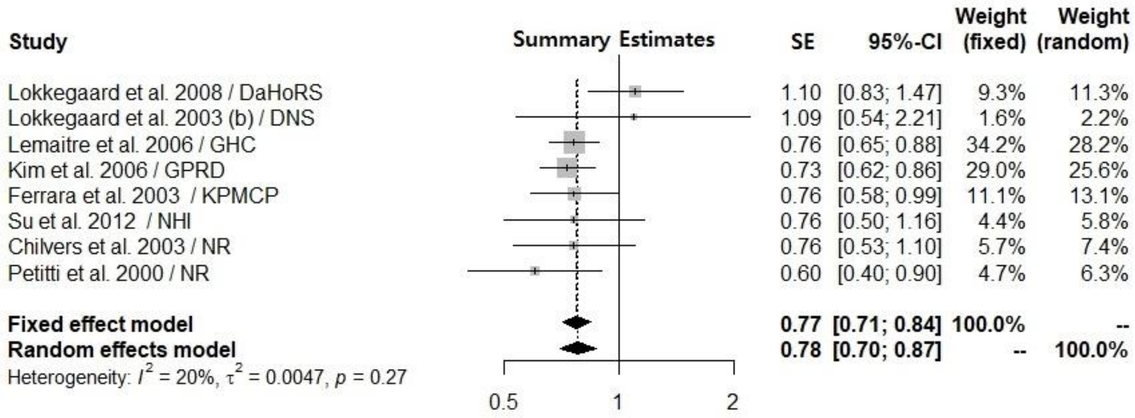

Summary estimates (95% CI) were measured by fixed-effect models if I^2 was <30% and P for heterogeneity was >0.05; otherwise, the summary estimates (95% CI) were measured by random-effect models. Forest and funnel plots were generated by "meta" package in R version 3.4.1. MHT, menopausal hormone therapy; MI, myocardial infarction; SE, summary estimates.

Supplementary Figure S4.4.2. MHT and MI in observational studies: subgroup results by duration of use.

(a) duration < 5 years

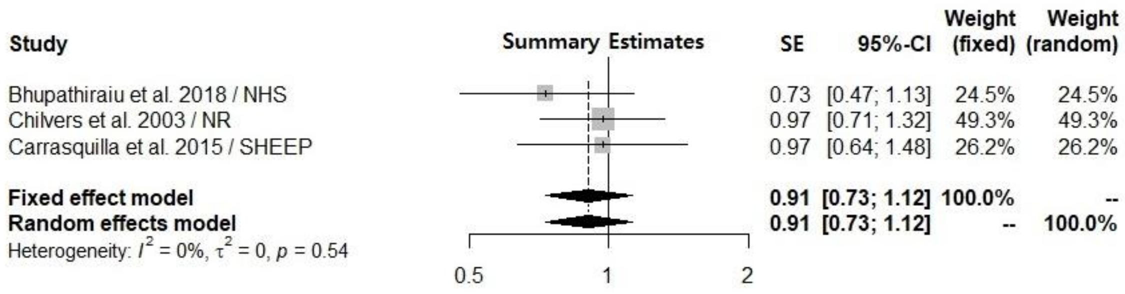

(b) duration ≥ 5 years

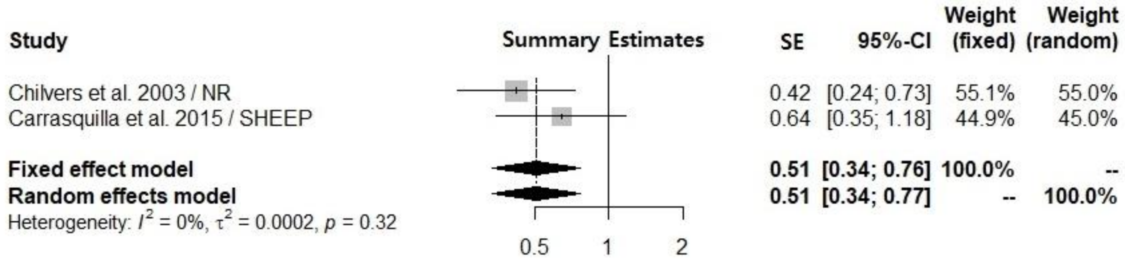

Summary estimates (95% CI) were measured by fixed-effect models if I^2 was <30% and P for heterogeneity was >0.05; otherwise, the summary estimates (95% CI) were measured by random-effect models. Forest and funnel plots were generated by "meta" package in R version 3.4.1. MHT, menopausal hormone therapy; MI, myocardial infarction; SE, summary estimates.

Supplementary Figure S4.4.3. MHT and MI in observational studies: subgroup results by timing of initiation.

(a) early users

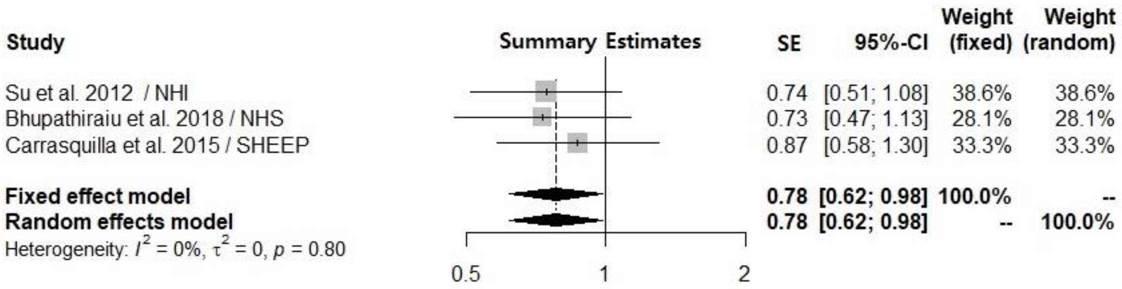

(b) late users

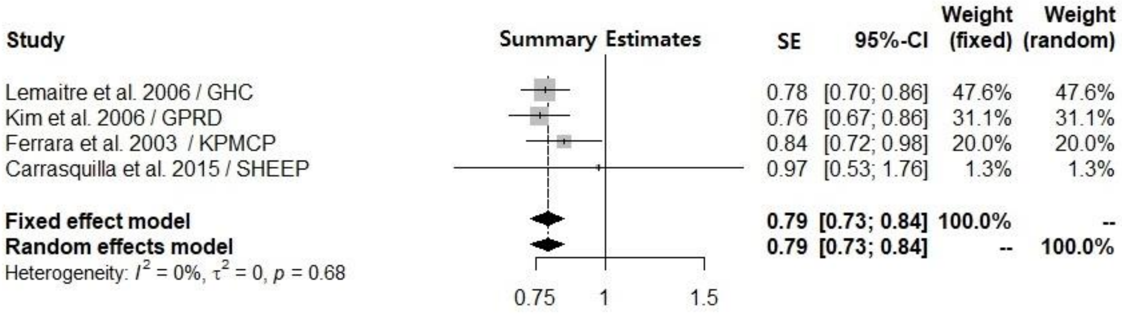

Summary estimates (95% CI) were measured by fixed-effect models if I^2 was <30% and P for heterogeneity was >0.05; otherwise, the summary estimates (95% CI) were measured by random-effect models. Forest and funnel plots were generated by "meta" package in R version 3.4.1. MHT, menopausal hormone therapy; MI, myocardial infarction; SE, summary estimates.

Supplementary Figure S4.4.4. MHT and MI in observational studies: subgroup results by route of administration.

(a) oral

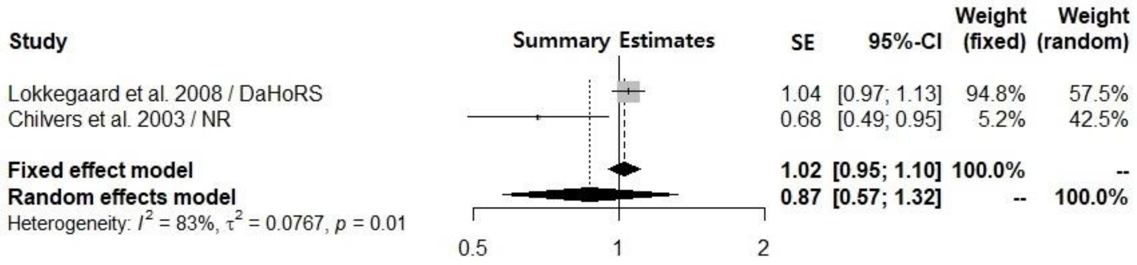

(b) non-oral

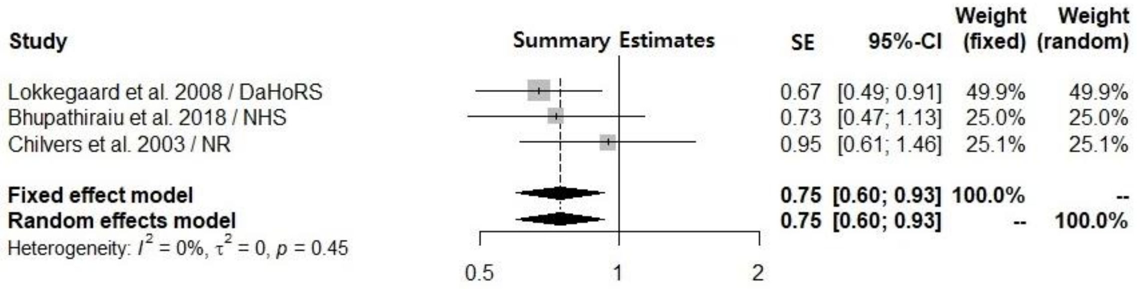

Summary estimates (95% CI) were measured by fixed-effect models if I^2 was <30% and P for heterogeneity was >0.05; otherwise, the summary estimates (95% CI) were measured by random-effect models. Forest and funnel plots were generated by "meta" package in R version 3.4.1. MHT, menopausal hormone therapy; MI, myocardial infarction; SE, summary estimates.

Supplementary Figure S4.4.5. MHT and MI in observational studies: subgroup results by underlying disease.

(a) women with diseases

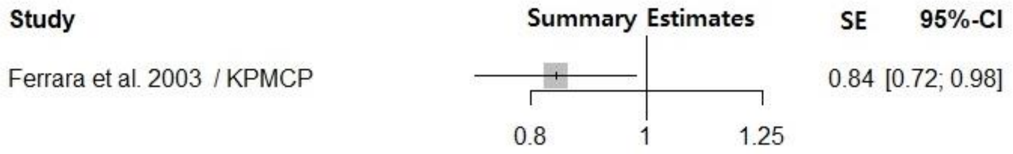

(b) women without diseases (relatively healthy)

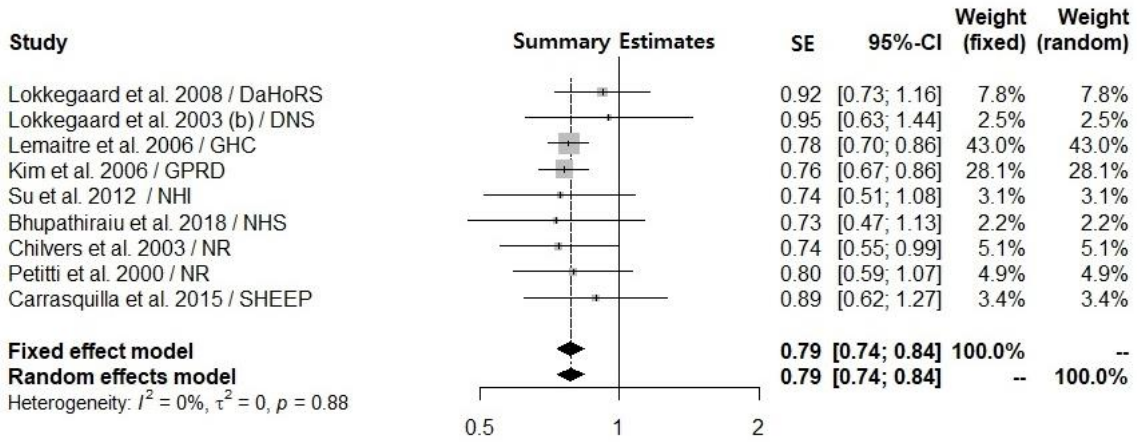

Summary estimates (95% CI) were measured by fixed-effect models if I^2 was <30% and P for heterogeneity was >0.05; otherwise, the summary estimates (95% CI) were measured by random-effect models. Forest and funnel plots were generated by "meta" package in R version 3.4.1. MHT, menopausal hormone therapy; MI, myocardial infarction; SE, summary estimates.

Supplementary Figure S4.4.6. MHT and MI in observational studies: subgroup results by recency of MHT.

(a) past

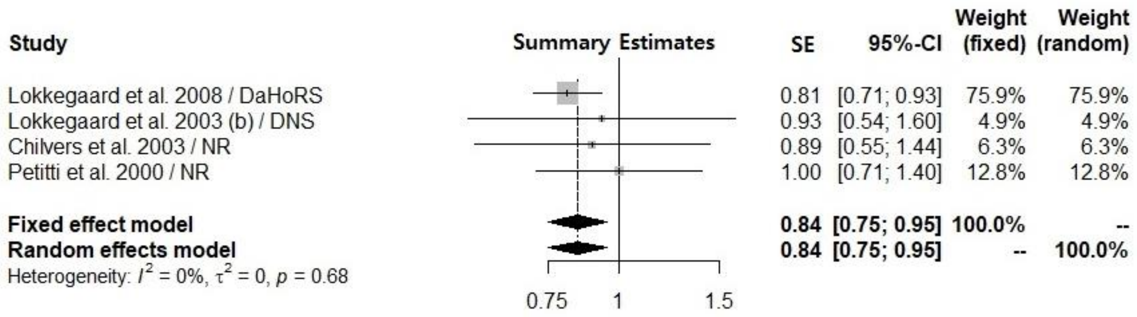

(b) current

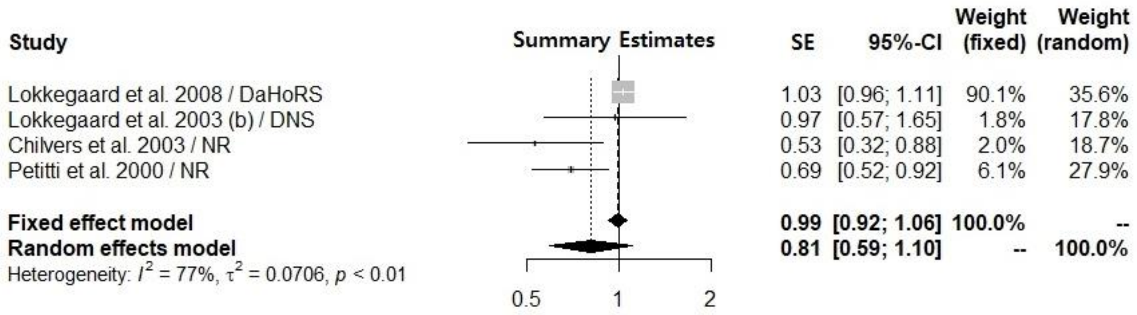

Summary estimates (95% CI) were measured by fixed-effect models if I^2 was <30% and P for heterogeneity was >0.05; otherwise, the summary estimates (95% CI) were measured by random-effect models. Forest and funnel plots were generated by "meta" package in R version 3.4.1. MHT, menopausal hormone therapy; MI, myocardial infarction; SE, summary estimates.

Supplementary Figure S4.4.7. MHT and MI in observational studies: subgroup results by study design.

(a) cohort

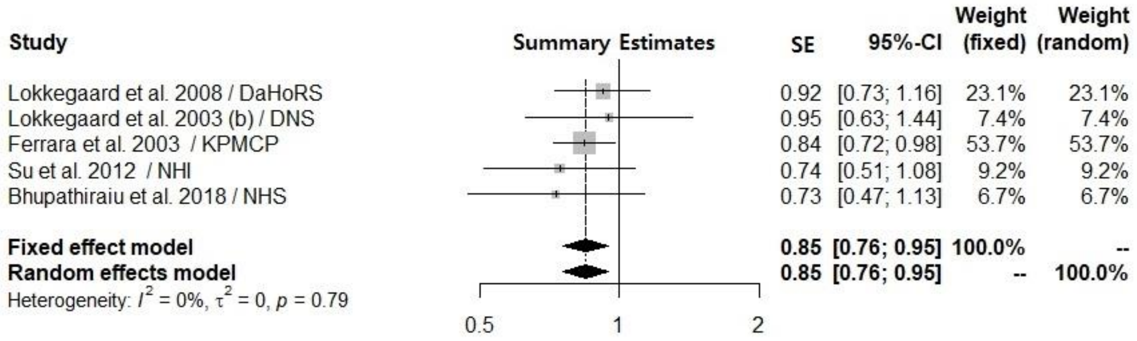

(b) case-control study

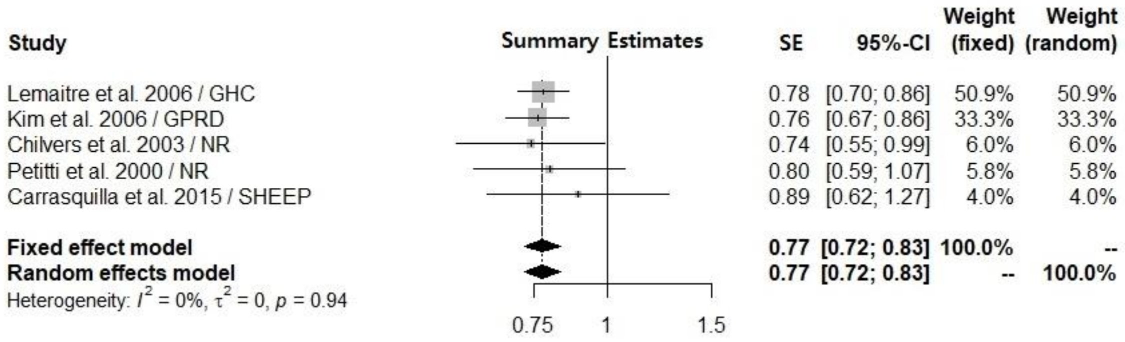

Summary estimates (95% CI) were measured by fixed-effect models if I^2 was <30% and P for heterogeneity was >0.05; otherwise, the summary estimates (95% CI) were measured by random-effect models. Forest and funnel plots were generated by "meta" package in R version 3.4.1. MHT, menopausal hormone therapy; MI, myocardial infarction; SE, summary estimates.

Supplementary Figure S4.4.8. MHT and MI in observational studies: subgroup results by study quality.

(a) good and fair

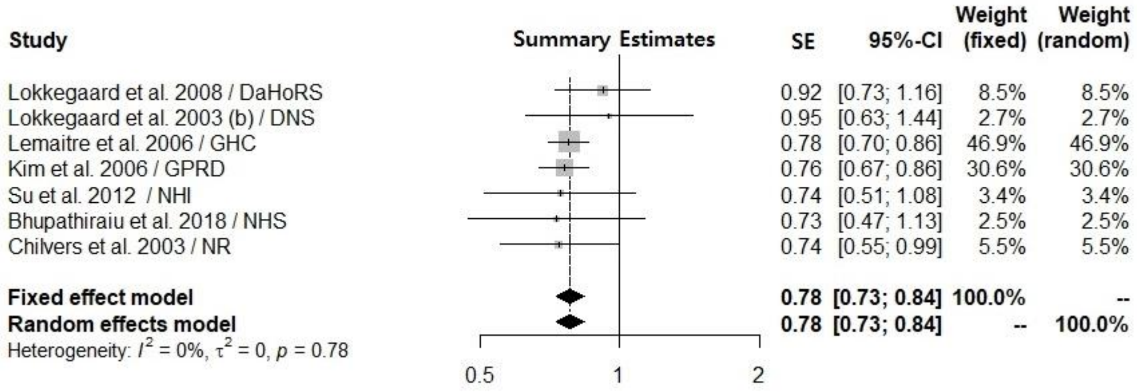

(b) poor

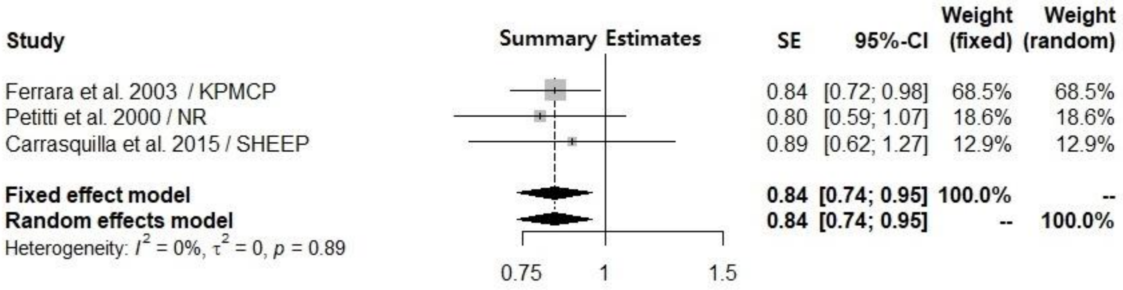

Summary estimates (95% CI) were measured by fixed-effect models if I^2 was <30% and P for heterogeneity was >0.05; otherwise, the summary estimates (95% CI) were measured by random-effect models. Forest and funnel plots were generated by "meta" package in R version 3.4.1. MHT, menopausal hormone therapy; MI, myocardial infarction; SE, summary estimates.
