# Supplementary material for: A systematic review and meta-analysis of effects of menopausal hormone therapy on cardiovascular diseases
Source: Sci Rep. 2020 Nov 26;10:20631. doi: 10.1038/s41598-020-77534-9 (PMC7691511; doi:10.1038/s41598-020-77534-9)
Supplement: Supplementary file 2 — Supplementary Information. [file 41598_2020_77534_MOESM2_ESM.pdf]

Supplementary material

A systematic review and meta-analysis of effects of menopausal hormone therapy on cardiovascular diseases

Ji-Eun Kim[1, 2], Jae-Hyuck Chang[3], Min-Ji Jeong[3], Jaesung Choi[1, 2], JooYong Park[1, 2], Chaewon Baek[3], Aesun Shin[4, 5, 6], Sang Min Park[1, 7], Daehee Kang[4, 5, 6, 8] and Ji-Yeob Choi[1, 2, 5, 9 *]

[1]Department of Biomedical Sciences, Seoul National University Graduate School, Seoul, Korea;

[2]BK21plus Biomedical Science Project, Seoul National University College of Medicine, Seoul, Korea

[3]Northeastern University Bouve College of Health Sciences School of Pharmacy, Boston, MA, USA;

[4]Department of Preventive Medicine, Seoul National University College of Medicine, Seoul, Korea;

[5]Cancer Research Institute, Seoul National University, Seoul, Korea;

[6]Department of Innovative Medical Science, Seoul National University College of Medicine, Seoul, Korea;

[7]Department of Family Medicine, Seoul National University College of Medicine, Seoul, Korea;

[8]Institute of Environmental Medicine, Seoul National University Medical Research Center, Seoul, Korea;

[9]Institute of Health Policy and Management, Seoul National University Medical Research Center, Seoul, Korea

Supplementary File 1. Search terms in PubMed and EMBASE

Supplementary Table S1. Summary of randomized controlled trials included in the meta-analysis

Supplementary Table S2. Summary of cohort studies included in the meta-analysis

Supplementary Table S3. Summary of nested case-control studies included in the meta-analysis

Supplementary Table S4. Summary of case-control studies included in the meta-analysis

Supplementary Table S5. The included studies in the meta-analysis of randomized controlled trials according to the inclusion criteria of the duplicated study population

Supplementary Table S6. The included studies in the meta-analysis of observational studies according to the inclusion criteria of the duplicated study population

Supplementary Table S7. Risk of bias assessment for randomized controlled trials using the Jadad scale

Supplementary Table S8. Risk of bias assessment for cohort studies using the Newcastle-Ottawa Scale

Supplementary Table S9. Risk of bias assessment for nested case-control studies using the Newcastle-Ottawa Scale

Supplementary Table S10. Risk of bias assessment for case-control studies using the Newcastle-Ottawa Scale

Supplementary information. Reference list of included studies in the meta-analysis

Supplementary File 1. Search terms in PubMed and EMBASE

1) PubMed (2000/01/01-2019/12/31)

- RCTs
: ("**Cardiovascular diseases**"[Mesh] OR "**Cerebrovascular Disorders**"[Mesh] OR ("cardiovascular"[TI] OR "coronary"[TI] OR "stroke"[TI] OR "myocardial"[TI] OR "myocardial infarction"[TI] OR "all-cause death"[TI] OR "cardiovascular death"[TI] OR "death"[TI] OR "mortality"[TI] OR "coronary heart disease"[TI] OR "coronary artery disease"[TI] OR "ischemic heart disease"[TI] OR "ischaemic heart disease"[TI] OR "heart attack"[TI] OR "Angina"[TI] OR "embol"[TI] OR "thrombo"[TI] OR "hypertension"[TI] OR "arteriosclerosis"[TI] OR "hyperlipidemias"[TI] OR "cholesterol"[TI] OR "blood coagulation factors" OR "lipid"[TI] OR "fibrin"[TI])) AND ("**Hormone replacement therapy**"[Mesh] OR "hormone therapy" OR "menopausal hormone therapy"[TI] OR "**postmenopause**"[Mesh] OR ("HRT"[TI] OR "ERT"[TI] OR "ORT"[TI] OR "Progestins"[TI] OR "medroxyprogestrone acetate" OR "dydrogesterone" OR "norethisterone" OR "norethindrone" OR "oestrogen"[TI] OR "estrogen"[TI] OR "CEE"[TI] OR "premarin"[TI] OR "estriol"[TI] OR "oestradiol"[TI])) AND ("trial"[TI] OR "randomized controlled trial" OR "clinical trials") NOT "animals" NOT "cohort" NOT "case-control study"

- Observational studies
: ("**Cardiovascular diseases**"[Mesh] OR "**Cerebrovascular Disorders**"[Mesh] OR ("cardiovascular"[TI] OR "coronary"[TI] OR "stroke"[TI] OR "myocardial"[TI] OR "myocardial infarction"[TI] OR "all-cause death"[TI] OR "cardiovascular death"[TI] OR "death"[TI] OR "mortality"[TI] OR "coronary heart disease"[TI] OR "coronary artery disease"[TI] OR "ischemic heart disease"[TI] OR "ischaemic heart disease"[TI] OR "heart attack"[TI] OR "Angina"[TI] OR "embol"[TI] OR "thrombo"[TI] OR "hypertension"[TI] OR "arteriosclerosis"[TI] OR "hyperlipidemias"[TI] OR "cholesterol"[TI] OR "blood coagulation factors" OR "lipid"[TI] OR "fibrin"[TI])) AND ("**Hormone replacement therapy**"[Mesh] OR "hormone therapy" OR "Menopausal hormone therapy"[TI] OR "**postmenopause**"[Mesh] OR ("HRT"[TI] OR "ERT"[TI] OR "ORT"[TI] OR "Progestins"[TI] OR "medroxyprogestrone acetate" OR "dydrogesterone" OR "norethisterone" OR "norethindrone" OR "oestrogen"[TI] OR "estrogen"[TI] OR "CEE"[TI] OR "premarin"[TI] OR "estriol"[TI] OR "oestradiol"[TI])) AND ("cohort" OR "case-control study") NOT "animals"

2) EMBASE (2000/01/01-2019/12/31)

- RCTs

: (‘**Cardiovascular diseases**’ OR ‘**Cerebrovascular Disorders**’ OR (‘cardiovascular’ OR ‘Coronary’ OR ‘Stroke’ OR ‘myocardial’ OR ‘myocardial infarction’ OR ‘all-cause death’ OR ‘cardiovascular death’ OR ‘death’ OR ‘mortality’ OR ‘coronary heart disease’ OR ‘coronary artery disease’ OR ‘ischemic heart disease’ OR ‘ischaemic heart disease’ OR ‘heart attack’ OR ‘Angina’ OR ‘embol’ OR ‘thrombo’ OR ‘hypertension’ OR ‘arteriosclerosis’ OR ‘hyperlipidemias’ OR ‘cholesterol’ OR ‘blood coagulation factors’ OR ‘lipid’ OR ‘fibrin’)) AND (‘**Hormone replacement therapy**’ OR ‘hormone therapy’ OR ‘menopausal hormone therapy’ OR ‘**postmenopause**’ OR (‘HRT’ OR ‘ERT’ OR ‘ORT’ OR ‘Progestins’ OR ‘medroxyprogestrone acetate’ OR ‘dydrogesterone’ OR ‘norethisterone’ OR ‘norethindrone’ OR ‘oestrogen’ OR ‘estrogen’ OR ‘CEE’ OR ‘premarin’ OR ‘estriol’ OR ‘oestradiol’)) AND (‘trial’ OR ‘randomized controlled trial’ OR ‘clinical trials’) NOT ‘animals’ NOT ‘cohort’ NOT ‘case-control study’

- Observational studies

: (‘**Cardiovascular diseases**’ OR ‘**Cerebrovascular Disorders**’ OR (‘cardiovascular’ OR ‘Coronary’ OR ‘Stroke’ OR ‘myocardial’ OR ‘myocardial infarction’ OR ‘all-cause death’ OR ‘cardiovascular death’ OR ‘death’ OR ‘mortality’ OR ‘coronary heart disease’ OR ‘coronary artery disease’ OR ‘ischemic heart disease’ OR ‘ischaemic heart disease’ OR ‘heart attack’ OR ‘Angina’ OR ‘embol’ OR ‘thrombo’ OR ‘hypertension’ OR ‘arteriosclerosis’ OR ‘hyperlipidemias’ OR ‘cholesterol’ OR ‘blood coagulation factors’ OR ‘lipid’ OR ‘fibrin’)) AND (‘**Hormone replacement therapy**’ OR ‘hormone therapy’ OR ‘menopausal hormone therapy’ OR ‘**postmenopause**’ OR (‘HRT’ OR ‘ERT’ OR ‘ORT’ OR ‘Progestins’ OR ‘medroxyprogestrone acetate’ OR ‘dydrogesterone’ OR ‘norethisterone’ OR ‘norethindrone’ OR ‘oestrogen’ OR ‘estrogen’ OR ‘CEE’ OR ‘premarin’ OR ‘estriol’ OR ‘oestradiol’)) AND (‘cohort’ OR ‘case-control study’) NOT ‘animals’

Supplementary Table S1. Summary of randomized controlled trials included in the meta-analysis

First author, year	Trial name	Participants (exposure/ non-exposure)	Country	Age (mean, years)	Follow-up duration (mean, years)	Regimen types	Treatment duration	Outcomes (no. of events in exposure/non-exposure)	Adjustment
Schierbeck et al. 2012[1]	DOPS	1,006 (502/504)	Denmark	49.7 (45-58)	15.8	**Combined EP**: 2 mg 17β-estradiol + 1 mg norethisterone acetate, **Estrogen:** 1mg or 2 mg 17β-estradiol	10.1	All-cause death (27/40), CVD death (6/23), VTE (4/5), PE (1/3), stroke (19/21), MI (5/11)	Age
Ouyang et al. 2006[2]	EAGAR	83 (40/43)<br><br>Underlying disease: coronary artery	USA	64 (>55+)	2.75	**Combined EP:** 1 mg 17ß-estradiol + 2.5 mg medroxyprogesterone **Estrogen:** 1 mg 17ß-estradiol	2.75	CVD death (2/4), MI (2/2), angina (12/10), revascularization (8/1)	NA
Hodis et al. 2016[3]	ELITE	643 (137/134 in early, 186/186 in late)	USA	early: 53.4, late: 63.6	7.5	**Combined EP**: 1 mg 17ß-estradiol + 45 mg micronized progesterone (as a 4% vaginal gel) **Estrogen**: 1mg 17ß-estradiol	7.5	All-cause death(1/1), VTE (3/2), PE (2/0), MI (1/3), angina (2/0)	NA
Tierney et al. 2009[4]	EMS	142 (70/72)<br><br>Underlying disease: pre-existing diseases	Canada	75 (61-87)	2	**Combined EP:** 1 mg 17β-estradiol micronized + 0.35 mg norethindrone	2	All-cause death (2/2), VTE (1/0), stroke (5/4), MI (0/1)	NA
Hodis et al. 2001[5]	EPAT	222 (111/111)	USA	62 (>44+)	2	**Estrogen**: 1 mg 17ß-estradiol micronized	2	MI (1/1), revascularization (1/2)	NA
Veerus et al. 2006[6]	EPHT	4,170 (898/880)	Estonia	58.8	3.43	**Combined EP:** 0.625 mg conjugated equine estrogen +2.5 mg (or 5 mg) medroxyprogesterone acetate	3.43	All-cause death (3/4), stroke (6/4), MI (3/2), CHD (171/159)	Age, oral contraceptive use and stratified by blinding
Herrington et al. 2000[7]	ERA	309 (204/105)<br><br>Underlying disease: coronary diseases	USA	65.8 (41-79)	3.2	**Combined EP:** 0.625 mg conjugated equine estrogen + 2.5 mg medroxyprogesterone acetate	3.2	All-cause death (11/6), CVD death (6/3), VTE (7/1), stroke (11/6), MI (12/7), CHD (57/34),	NA

						**Estrogen:** 0.625 mg conjugated equine estrogen		angina (33/22), revascularization (38/24)	
The ESPRIT team, 2002[8]	ESPRIT	1,017 (513/504)<br><br>Underlying disease: MI	UK	62.6 (50-69)	2	**Estrogen:** 2 mg estradiol valerate	2	CVD death (21/30), VTE (5/4), PE (3/3), stroke (10/6)	Age, reported history of diabetes, high blood pressure, hysterectomy, BMI, and smoking habit
Cherry et al. 2014[9]	ESPRIT	1,017 (513/504)<br><br>Underlying disease: MI	UK	62.6 (50-69)	14.1	**Estrogen:** 2 mg estradiol valerate	2	All-cause death (214/204)	Age
Greenspan et al. 2005[10]	Greenspa n	373 (187/186)	USA	71.2 (65-90)	3	**Combined EP:** 0.625 mg conjugated equine estrogen + 2.5 mg medroxyprogesterone<br>**Estrogen:** 0.625 mg conjugated equine estrogen	3	All-cause death (1/2), VTE (2/1), MI (1/3)	NA
Grady et al. 2002[11]	HERS I, II	2763 (1380/1383)<br><br>Underlying disease: CHD	USA	67	6.8	**Combined EP:** 0.625 mg conjugated estrogen + 2.5 mg medroxyprogesterone acetate	6.8	Stroke (171/158/), MI (183/196), CHD (290/293), angina (137/157), revascularization (255/253)	NA
Hulley et al. 2002[12]	HERS I, II	2763 (1380/1383)<br><br>Underlying disease: CHD	USA	67	6.8	**Combined EP:** 0.625 mg conjugated estrogen, 2.5 mg medroxyprogesterone acetate	6.8	All-cause death (261/239), CVD death (159/144), VTE (54/25), PE (17/6)	NA
Gleason et al. 2015[13]	KEEPS	727 (452/275)	USA	52.6 (>65+)	4	**Combined EP:** 0.45 mg CEE + 200 mg micronized progesterone,<br>0.05 mg estradiol (transdermal) + 200 mg micronize d progesterone	4	VTE (1/1), stroke (1/0)	NA
Clarke et al. 2002[14]	PHASE	255 (134/121)<br><br>Underlying disease: CHD	UK	66.3	2.56	**Combined EP**: 3 mg 17ß-estradiol + 4 mg norethisterone (transdermal)<br>**Estrogen**: 2.5 mg transdermal 17ß-estradiol (transdermal)	2.56	All-cause death (8/3), CVD death (6/1), MI (1/4), angina (46/32)	NA

Gallagher et al. 2001[15]	STOP IT	489 (121/123)	USA	71 (65-77)	3	**Combined EP:** 0.625 mg conjugated equine estrogen + 2.5 mg medroxyprogesterone acetate<br>**Estrogen:** 0.625 mg conjugated equine estrogen	3	All-cause death (1/1), VTE (2/1), stroke (4/3), MI (8/3)	NA
Waters et al. 2002[16]	WAVE	423 (210/213)<br><br>Underlying disease: coronary stenosis	USA, Canada	65	3	**Combined EP:** 0.625 mg conjugated equine estrogen +2.5 mg medroxyprogesterone acetate<br>**Estrogen:** 0.625 mg conjugated equine estrogen	3	All-cause death (14/8), CVD death (8/6), VTE (4/4), stroke (9/4), MI (4/4), revascularization (34/44)	NA
Hodis et al. 2003[17]	WELL-HART	226 (150/76)<br><br>Underlying disease: coronary artery disease	USA	63.5 (48-75)	3.3	**Combined EP:** 1 mg 17β-estradiol + 5 mg medroxyprogesterone acetate<br>**Estrogen:** 1 mg 17β-estradiol	3.3	All-cause death (5/8)	NA
Viscoli et al. 2001[18]	WEST	664 (337/327)<br><br>Underlying disease: stoke or TIA	USA	71 (46-91)	2.8	**Estrogen:**1 mg 17ß-estradiol	2.8	All-cause death (99/93), CVD death (11/13), VTE (3/4), PE (2/2), stroke (63/56), MI (14/12)	Adjusted relative risk
Manson et al. 2003[19]	WHI I	16608 (8506/8102)<br><br>Underlying disease: pre-existing diseases	USA	63.3 (50-79)	5.2 (median)	**Combined EP:** 0.625 mg conjugated equine estrogen + 2.5 mg medroxyprogesterone acetate	5.2	Angina (172/195), revascularization (214/205)	Adjusted hazard ratio
Rossouw et al. 2002[20]	WHI I	16608 (8506/8102)<br><br>Underlying disease: pre-existing diseases	USA	63.2 (50-79)	5.2 (median)	**Combined EP:** 0.625 mg conjugated estrogen + 2.5 mg medroxyprogesterone acetate	5.2	VTE (144/66)	Adjusted hazard ratio
Anderson et al. 2004[21]	WHI II	10739 (5310/5429)<br><br>Underlying disease: pre-existing diseases	USA	63.6 (50-79)	6.8 (median)	**Estrogen:** 0.625 mg conjugated equine estrogen	6.8	VTE (101/78)	Adjusted hazard ratio
Hsia et al. 2006[22]	WHI II	10739 (5310/5429)<br><br>Underlying disease: pre-existing diseases	USA	63.6 (50-79)	6.8 (median)	**Estrogen:** 0.625 mg conjugated equine estrogen	6.8	Angina (163/171), revascularization (253/276)	NA

Manson et al. 2013[23]	WHI I, II	27347 (combined EP: 5310/5429, Estrogen: 8506/8102)<br><br>Underlying disease: pre-existing diseases	USA	63 (50-79)	13 (median)	**Combined EP:** 0.625 mg conjugated estrogen + 2.5 mg medroxyprogesterone acetate<br>**Estrogen:** 0.625 mg conjugated equine estrogen	combined EP: 5.6 , Estrogen: 7.2	Stroke (376/311, 278/253), PE (172/128,107/96), MI (389/324, 285/288), CHD (487/430, 363/393)	NA
Manson et al. 2017[24]	WHI I, II	27347 (combined EP: 5310/5429, Estrogen: 8506/8102)<br><br>Underlying disease: pre-existing diseases	USA	50-79	18 (median)	**Combined EP:** 0.625 mg conjugated estrogen + 2.5 mg medroxyprogesterone acetate<br>**Estrogen:** 0.625 mg conjugated equine estrogen	combined EP: 5.6 , Estrogen: 7.2	All-cause death (2244/2110, 1505/1630), CVD death (688/644, 547/577)	NA
Collins et al. 2006[25]	WHISP	100 (49/51)<br><br>Underlying disease: acute coronary syndrome	UK	69 (>55+)	0.7 (median)	**Combined EP:** 1 mg 17ß-estradiol + 0.5 mg norethisterone acetate	0.7	All-cause death (1/2), CVD death (1/2), VTE (1/1), stroke (0/1), MI (3/6)	NA
Vickers et al. 2007[26]	WISDOM	5,692 (2196/2189)	UK, New Zealand, Australia	62.8 (50-69)	1.06	**Combined EP:** 0.625 mg conjugated equine estrogen + 2.5 mg medroxyprogesterone acetate	1.06	All-cause death (8/5), VTE (23/3), PE (10/2), MI (4/0), angina (3/0)	NA

CHD, coronary heart disease; CVD, cardiovascular disease; DOPS, Danish Osteoporosis Prevention Study; EAGAR, Estrogen And Graft Atherosclerosis Research; ELITE, Early versus Late Intervention Trial with Estradiol; EMS, Estrogen Memory Study; EPAT, Estrogen in Prevention Atherosclerosis Trial; EPHT, Estonian Postmenopausal Hormone Therapy; ERA, Estrogen Replacement and Atherosclerosis; ESPRIT, Estrogen for the Prevention of Re-Infarction Trial; HERS, Heart and Estrogen/Progestin Replacement Study; KEEPS, Kronos Early Estrogen Prevention Study; MI, myocardial infarction; NA, not applicable; PE, pulmonary embolism; PHASE, Papworth HRT Atherosclerosis; VTE, venous thromboembolism; WAVE, Women's Angiographic Vitamin and Estrogen; WELL-HART, Women's Estrogen Progestin Lipid-Lowering Hormone Atherosclerosis; WEST, Women's Estrogen for Stroke Trial; WHI, Women's Health Initiative; WHISP, Women's Hormone Intervention Secondary Prevention Study; WISDOM, Women's International Study of long Duration Oestrogen after Menopause.

Supplementary Table S2. Summary of cohort studies included in the meta-analysis

First author, year	Study name	Participants (exposure/non-exposure or exposure ratio)	Country	Age (mean, years)	Follow-up duration (mean, years)	Exposure (extracted data) vs. never users	Outcomes (no. of exposure/non-exposure)	Adjustment
Apostolakis et al. 2014[27]	AFFIRM	1,594 (376/1,218) (23.6%)<br><br>Underlying disease: atrial fibrillation	UK	71 (65-74)	3.5 median	Ever (HR)	All-cause death (41/270), stroke (13/67)	Age, prior stroke, hypertension, diabetes mellitus, heart failure, prior MI, peripheral arterial disease, warfarin treatment on recruitment, and treatment arm
Chen et al. 2017[28]	ALSWH	13,715 (2,582/11,099) (18.8%)	Australia	ever: 50, never: 49.6 (47-52)	1998-2013	Ever (HR)	CVD death (12/117)	Age, education, and self-rated health
Wilson et al. 2019[29]	ALSWH	13,529 (3,664/9,865) (27.1%)	Australia	NR (45-50)	21.5 median	Ever (N[*])	All-cause death (290/611)	NA
Alexander et al. 2001[30]	CARS	1,857 (413/1,333) (28%)<br><br>Underlying disease: MI	USA, Canada	ever: 59, never: 67 (>50+)	1.25 median	Ever (RR)	All-cause death (2/6), angina (24/20)	All-cause death: Age, previous angina, congestive heart failure, current smoker, ejection fraction, hypertension, prior MI, peripheral vascular disease, prior stroke or TIA, race, weight, randomized treatment and HRT (prior/current, new, or never)<br>Angina: Age, history of angina, atrial fibrillation, systolic BP, prior percutaneous transluminal coronary angioplasty, and HRT use

Stram et al. 2011[31]	CTS	71,237 (49,338/17,874)	USA	63 (36-94)	1995-2004	Ever (HR*), Past/current (HR), Estrogen/combined EP (N), Duration (N), Timing of initiation (N)	All-cause death (4,670/3,268)	Age, status of hormone therapy and their interaction, stratified by race, BMI, smoking, total pack-years, alcohol consumption, physical activity, dietary intake, other hormone, and prior history of heart attack, stroke, cancer and diabetes
Lokkegaard et al. 2008[32]	DaHoRS	698,098 (26%)	Denmark	NR (51-69)	1995-2001	Ever (RR*), Past/current (RR), Estrogen (RR)/combined EP (RR†), Oral/non-oral (RR†),	MI (1,080/3,742)	Age, calendar year, education, employment status, habitation and medication for hypertension, heart conditions, hyperlipidemia, or diabetes
Lokkegaard et al. 2017[33]	DaHoRS	980,003 (36%)	Denmark	NR (51-79)	7.9	Ever (RR*), Past/current (RR), Estrogen (RR)/combined EP (RR†), Oral/non-oral (RR†), Duration (RR†)	Stroke (6,240/12,788)	Age, calendar year, education, medication for diabetes, arrhythmia, hypertension, diuretics, hyperlipidemia, and anticoagulation, and bilateral oophorectomy
Holm et al. 2019[34]	Diet, Cancer and Health	29,243 (13,303/15,904) (45.5%)	Denmark	56 median (50-64)	17.6 median	Ever (HR*), Past/current (HR), Oral/non-oral (HR), Estrogen (HR)/combined EP (HR†)	All-cause death (1,966/2,132), CVD death (315/356)	Age, alcohol, smoking, BMI, physical activity and level of education
Lokkegaard et al. 2003[35] (a)	DNS	13,122 (5,547/7,575) (42.3%)	Denmark	NR (>45+)	1993-1998	Ever (HR), Past/current (HR), Estrogen/combined EP (HR)	Stroke (58/86)	smoking history, alcohol consumption, BMI, physical activity, hypertension, angina, diabetes mellitus, and metabolic disease

Lokkegaard et al. 2003[36] (b)	DNS	13,084 (5,508/7,558) (42%)	Denmark	NR (>45+)	1993-1998	Ever (HR), Past/current (HR), Estrogen/combined EP (HR)	All-cause death (347/624), CHD (153/198), MI (40/68)	Age, familial predisposition, smoking, alcohol consumption, BMI, physical activity, hypertension, angina, diabetes, thyroid disease and self-reported health
Canonico et al. 2010[37]	E3N	80,308 (NA)	France	54 (45-70)	10.1	Ever (HR*), Past (HR)/current (HR†), Oral/non-oral (HR)	VTE (321/181)	Age, BMI, parity, education and time-period
Newton et al. 2003[38]	GHC	770 (419/351)<br><br>Underlying disease: diabetes	USA	68.9 (45-80)	6.8 median	Ever (RR*), Past (RR)/current (RR†), Estrogen/combined EP (RR)	CHD (321/306)	Age, duration of diabetes, insulin use, history of MI, angina, congestive heart failure, stroke, peripheral vascular disease, lower extremity amputation, lower extremity ulcer, revascularization, and current smoking
Tannen et al. 2007[39]	GPRD	18,462 (6,890/11,572)	UK	60.5 (55-79)	5.5	Ever (HR), Estrogen (HR)	All-cause death (NR), PE (NR)	Age, BP, BMI, smoking, previous MI or stroke and evidence for angina or other ischaemic cerebrovascular disease, heart failure or diabetes mellitus
Weiner et al. 2008[40]	GPRD	50,756 (20,654/30,102)	UK	50-79	5	Ever (HR**), Combined EP (HR*)	All-cause death (548/1,807), PE (97/156)	Age, BP, BMI, smoking, previous MI or stroke and evidence for angina or other ischaemic cerebrovascular disease, heart failure or diabetes mellitus

Ferrara et al. 2003[41]	KPMCP	24,420 (4,614/19,806) (19%) Underlying disease: diabetes	USA	64.9 (>50+)	3.04	Ever (RH), Estrogen/combined EP (RH)	MI (180/674)	Age, ethnicity, education, obesity, diabetes duration, hypoglycemic therapy, glycosylated hemoglobin, hypertension, lipid-lowering medications, smoking, alcohol and exercise
Ohira et al. 2010[42]	LITE	8,236 (NA)	USA	NR (>45+)	11.8	Ever (RR*), Past/current (RR), Oral (RR*)	VTE (66/120)	Age, race, BMI, diabetes mellitus, and factor VIII
Mares et al. 2008[43]	MISSION	4,949 (2,693/2,256)	France	ever: 60.6, never: 64.2 (NR)	1	Ever (RR)	CHD (3/3)	NA
Sweetlands et al. 2012[44]	MWS	1,058,259 (581,548/476,711) (55%)	UK	56.7 (50-64)	3.1	Ever (RR*), Past/current (RR), Estrogen/combined EP (RR), Oral (RR†)/non-oral (RR), Duration (RR†)	VTE (1,235/965), PE (NR)	Age, region of residence, socioeconomic quintile and BMI, acute MI, heart failure, inflammatory bowel disease, respiratory failure, stroke, varicose vein
Su et al. 2012[45]	NHI	16,045 (combined EP: 4,712/8,070, E only: 1,208/2,055)	Taiwan	NR (50-79)	9.2 median	Ever (HR**), Estrogen/combined EP (HR)	All-cause death (234/639), CVD death (12/20), PE (12/18), stroke (265/628), MI (41/89)	Age, statin use, aspirin use, hypercholesterolemia, diabetes medication use, and hypertension
Chen et al. 2015[46]	NHI	1,712 (428/1,284, 1:3 matched cohort) Underlying disease: diabetes	Taiwan	59 median (>55+)	5 (exposure duration 6.5 months)	Ever (HR), Estrogen (HR), Oral (HR)	Stroke (4/39)	Age, comorbidities (hypertension, hyperlipidemia, chronic kidney disease, coronary artery disease, and heart failure), and

								medications (aspirin or clopidogrel)
Lee et al. 2015[47]	NHI	358,702 (179,351/179,351 matched cohort)	Taiwan	ever: 60.7, never: 59.5 (>50+)	2	Ever (HR), Oral (HR)	VTE (NR)	medications and comorbidities
Huang et al. 2018[48]	NHI	28,860 (3,249/25,611) (11.3%)	Taiwan	52.2 (45-100)	3	Ever (HR), Duration (HR)	CHD (1,974/1,429)	Age, sex, monthly income, charlson comorbidity scores, comorbidity, and long-term medications
Grodstein et al. 2000[49]	NHS	70,533 (NA)	USA	NR (<50-75)	1976-1996	Ever (RR*), Past/current (RR), Estrogen/combined EP (RR), Oral (RR†) Duration (RR†)	Stroke (455/312), CHD (596/662)	Age, BMI, history of diabetes, hypertension, high cholesterol level, age at menopause, cigarette smoking, and parental history of premature heart disease
Bhupathiraju et al. 2018[50]	NHS	53,797 (896/52,901) (1.7%)	USA	Ever: 54.8 Never: 54.8	18	Ever (HR), Estrogen (HR), Non-oral (vaginal, HR), Duration (HR)	Stroke (22/1,188) MI (20/1,339) VTE (11/524)	Age, calendar time, race, smoking status, alcohol intake, physical activity, BMI, age at menopause, hysterectomy, bilateral oophorectomy, history of past systemic hormone therapy use, parental history of cancer, history of blood pressure, hypercholesterolemia, history of diabetes, and parental history of early MI
Shlipak et al. 2001[51]	NRMI-3	114,724 (7,353/107,371) (6.4%)<br><br>Underlying disease: MI	USA	ever: 77, never: 71 (>55+)	1998-2000	Ever (N)	All-cause death (7/16), Stroke (2/2)	NA

Pentti et al. 2006[52]	OSTRPE	11,667 (6,157/5,519) (52.7%)	Finland	57.3 (52-62)	6.7	Ever (HR[**]), Duration (HR)	All-cause death (158/203), CVD death (21/37)	Age, BMI, parity, no. of chronic health disorders, hysterectomy and bilateral oophorectomy
Alexandersen et al. 2006[53]	PERF	1,280 (553/727) (43%)	Denmark	55 (44-75)	9.8	Ever (HR)	All-cause death (51/123), CVD death (14/47)	Age, BMI, and smoking habits
Ryan et al. 2012[54]	Three-City Study (3C)	5,135 (1,559/3,576) (30.4%)	France	Current: 70.3<br>Past: 73.2<br>Never: 75.2 (>65+)	5.2 median	Ever (HR[*]),<br>Past/current (HR),<br>Estrogen (HR)/combined EP (HR[†]),<br>Oral/non-oral (HR),<br>Timing of initiation (HR)	All-cause death (68/284)	Age, education, recruitment centre, living situation, comorbidity, depressive symptoms and cognitive impairment
Crandall et al. 2018[55]	WHI-OS	45,663 (4,100/41,563) (9%)	USA	ever: 65.5, never: 64.8 (50-79)	7.2 median	Ever (HR),<br>Estrogen (HR),<br>Non-oral (vaginal, HR)	All-cause death (59/2,592), VTE (12/600), stroke (20/902), CHD (20/1,311)	Age, education, past estrogen use, history of diseases (cancer, CVD, DVT/PE, hysterectomy status, race, BMI, diagnosis of diabetes, physical activity, hypertension, gail breast cancer risk score, fracture, smoking, income, and alcohol use
Graff-Iversen et al. 2004[56]	NR	14,324 (702/13,622) (4.9%)	Norway	ever: 48.8, never: 51.2 (35-62)	14	Ever (RR),<br>Combined EP (RR)	All-cause death (41/1,141), CVD death (7/324)	Age, self-reported CVD, diabetes and antihypertensive medication

[*]The estimates as ever users were calculated by combining the estimates as past and current users[31-34,37,38,42,44,49,54]; [**]the estimates as ever users were calculated by combining the various estimates (i.e., hysterectomy status[29], age of <55/>55 years[40], estrogen/combined EP[45], and duration of <5/>5 years[52], respectively); [†]the estimates in the subgroup analyses were calculated by combining the estimates of each study (not shown).

Data for extraction: HR, hazard ratio; RR, relative risk; N, number; RH: relative hazard.

AFFIRM, Atrial Fibrillation Follow-Up Investigation of Rhythm Management; ALSWH, Australian Longitudinal Study on Women's Health; BMI, body mass index; CARS, The Coumadin Aspirin Reinfarction Study; CHD, coronary heart disease; CTS, The California Teachers Cohort; CVD, cardiovascular disease; DaHoRS, The Danish Sex Hormone Register Study; DNS, The Danish Nurses Study; E3N, Etude Epidemiologique de femmes de l'Education Nationale; GHC, The Group Health Cooperative; GPRD, General Practice Research Database; KPMCP, Kaiser Permanente Medical Care Program; LITE, The Longitudinal Investigation of Thromboembolism Etiology; MI, myocardial infarction; MWS, Million Women Study; NA, not applicable; NHI, National Health Insurance; NHS, Nurses' Health Study; NR, not reported; NRMI-3, National Registry of Myocardial Infarction-3; OSTPRE, Kuopio Osteoporosis Risk Factor and Prevention; PE, pulmonary embolism; PERF, Prospective Epidemiological Risk Factors; VTE, venous thromboembolism; WHI-OS, The Women's Health Initiative Observational Study.

Supplementary Table S3. Summary of nested case-control studies included in the meta-analysis

First author, year	Study name	No. of case/control (exposure/non-exposure or exposure ratio)	Country	Age (mean, years)	Follow-up duration (mean, years)	Exposure (extracted data) vs. never users	Outcomes (no. of exposure/non-exposure)	Adjustment
Canonico et al. 2016[57]	French NHI	Case: 3,144 /control: 12,158 (1,021/14,281) (6.7%)	France	case: 56.7, control: 56.6 (51-62)	2009-2011	Ever (OR*), Oral/transdermal (OR)	Stroke (194/2,950)	Age, antidiabetic medication, antihypertensive medication, anti-dyslipidemia medication, and long-term chronic disease
Renoux et al. 2008[58]	GPRD	Case: 15,710/ control: 59,958 (3,187/70,330) (4.5%)	UK	70.3 (50-79)	6.7	Ever (OR*) Estrogen/combined EP (OR)	Stroke (721/14,496)	Age, ponderal status, smoking status, alcohol abuse, diabetes, hyperlipidemia, hypertension, atrial fibrillation, cardiovascular disease, transient ischemic attack and aspirin or other NSAID use and history of hysterectomy or oophorectomy
Renoux et al. 2010[59] (a)	GPRD	Case: 15,710/ control: 59,958 (3,187/70,330) (4.5%)	UK	70.3 (50-79)	6.7	Oral/transdermal (RR)	Stroke (721/14,496)	Age, BMI, smoking status, alcohol misuse, diabetes, hyperlipidaemia, hypertension, atrial fibrillation, cardiovascular disease, transient ischaemic attack, aspirin or other NSAID use, and history of hysterectomy or oophorectomy
Renoux et al. 2010[60] (b)	GPRD	Case: 23,505/ control: 231,562 (33,233/221,834) (13%)	UK	Case: 65.9, Control: 65.8 (50-79)	NR	Ever (RR*), Past/current (RR), Estrogen/combined EP (RR), Oral/non-oral (patch, RR*)	VTE (3,656/19,849)	Age, BMI, varicose veins, smoking status, immobilization, surgery, trauma or fracture, cancer, hypertension, cardiovascular or cerebrovascular disorder, myeloproliferative syndrome, inherited thrombophilia, screening for inherited thrombophilia, tamoxifen and NSAID
Lee et al. 2015[47]	NHI	Case: 3,833 /control: 76,660 (7,183/73,310)	Taiwan	65.1 (>50+)	2	Estrogen/combined EP (OR)	VTE (511/6,672)	Age, enrollment year, history of varicose veins, diagnosed cancer, hypertension, chronic lung disease, renal insufficiency, cardiovascular and cerebrovascular disease, major surgery

*The estimates as ever users were calculated by combining the various estimates (i.e., oral/non-oral[57], estrogen/combined EP[58] and past/current[60], respectively); †the estimates in the subgroup analyses were calculated by combining the estimates of each study (not shown).

Data for extraction: RR, relative risk; OR, odds ratio.
BMI, body mass index; GPRD, General Practice Research Database; NHI, National Health Insurance; NR, not reported; NSAID, Nonsteroidal anti-inflammatory drug; VTE, venous thromboembolism.

Supplementary Table S4. Summary of case-control studies included in the meta-analysis

First author, year	Study title	No. of case/control (exposure/non-exposure or exposure ratio)	Origin of control	Country	Age (mean, years)	Exposure (extracted data) vs. never users	Outcomes (no. of exposure/non-exposure)	Adjustment
Scarabin et al. 2003[61]	ESTHER	155/381 (257/279) (47.9%)	Hospital	France	62 (45-70)	Estrogen/combined EP (OR*), Duration (OR†)	VTE (84/71)	centre, age, and time of recruitment, BMI, familial history of VTE, history of varicose veins, education level
Canonico et al. 2007[62]	ESTHER	271/610 (488/392) (55.3%)	Hospital and population	France	Case: 61.6, /control: 61.5 (45-70)	Ever (N*), Past/current (N), Oral/transdermal (OR)	VTE (164/106)	Age, center, obesity, family history of VTE, history of varicose veins, education, age at menopause, hysterectomy, and cigarette smoking
Smith et al. 2004[63]	GHC	586/2,268 (1,043/1,811) (36.5%)	Population	USA	Case: 69.4, /control: 68.3 (30-89)	Ever (N*), Estrogen/combined EP (N), Oral (N†)	VTE (214/372)	NA
Lemaitre et al. 2006[64]	GHC	2,724/4,205 (NA)	Population	USA	Mean 67-70	Ever (N*), Estrogen/combined EP (N)	Stroke (290/790), MI (374/1,270)	NA
Kim et al. 2006[65]	GPRD	22,225/144,085 (4,065/162,245) (2.4%)	Population	UK	73	Ever (OR), Estrogen/combined EP (OR)	MI (475/21,750)	Age, hyperlipidaemia, hypertension, atheroma, diabetes, history of angina, smoking, alcohol, BMI, aspirin, cardiovascular drug use, consultation rate, atrial fibrillation, peripheral vascular disease, stroke and heart failure
Roach et al. 2013[66]	MEGA	1,082/1,468 (190/2,127) (8.9%)	Population	Netherlands	59 (50-70)	Ever (OR*) Estrogen/combined EP (OR), Oral/non-oral (OR), Duration (N)	VTE (88/823)	Age, BMI, smoking, and family history of venous thrombosis
de Lecinana et al. 2007[67]	PIVE	430/905 (87/1,248) (6.5%)	Hospital	Spain	68.9 (46-93)	Ever (OR)	Stroke (22/408)	Age

Carrasquilla et al. 2015[68]	SHEEP	347/499 (292/554) (34.5%)	Population	Sweden	Case: 62.4, /control: 63.2 (45-70)	Ever (OR), Duration (OR), Timing of initiation (OR)	MI (101/246)	Age, residential area, hysterectomy, oophorectomy, current smoking, ex-smoking, physical activity, alcohol consumption, BMI, and socioeconomic status
Bergendal et al. 2012[69]	THES	524/576 (230/869) (20.9%)	Population	Sweden	59 median	Ever (OR)	VTE (138/386)	Age, BMI, smoking, use of hormones, bedrest/minor trauma, surgery, cast, prothrombin mutation and/or factor V Leiden
Petitti et al. 2000[70]	NR	410/411 (467/354) (56.9%)	Hospital	USA	NR (45-74)	Ever (OR*) Past (OR)/current (OR†), Estrogen/combined EP (OR)	MI (224/186)	Age and facility
Hippisley -Cox et al. 2003[71]	NR	417/2,435 (367/2,485) (12.9%)	Hospital	UK	Case: 68.7, /control: 68.2	Ever (OR), Past/current (OR), Estrogen/combined EP (OR), Oral/non-oral (OR)	CHD (60/357)	Age, diabetes, hypertension, BMI, and smoking
Chilvers et al. 2003[72]	NR	559/1,118 (625/1,052) (45.4%)	Population	UK	NR (35-65)	Ever (OR), Past (OR†)/current (OR), Estrogen/combined EP (OR), Oral/non-oral (OR), Duration (OR†)	MI (188/371)	Age, diabetes, hypertension, smoking, alcohol, social class, family history and a health conscious behavior score
Douketis et al. 2005[73]	NR	95/610 (219/486) (31.1%)	Hospital	Canada, Italy, Netherlands	NR	Ever (OR), Past/current (N), Estrogen/combined EP (OR), Oral/transdermal (N†)	VTE (36/59)	hysterectomy and clinical center

*The estimates as ever users were calculated by combining the various estimates (i.e., oral/non-oral[61,63,64,66], and past/current[62,70], respectively); †The estimates in the subgroup analyses were calculated by combining the estimates of each study (not shown).
Data for extraction: OR, odds ratio; N, number.; BMI, body mass index; CHD, coronary heart disease.
ESTHER, The Estrogen and Thromboembolism Risk; GHC, The Group Health Cooperative; GPRD, General Practice Research Database; MI, myocardial infarction; NA, not applicable; NR, not reported; PIVE, Protection against stroke as a function of estrogenic lifetime; SHEEP, The Stockholm Heart Epidemiology Program; TEHS, The Thrombo Embolism Hormone Study; VTE, venous thromboembolism.

Supplementary Table S5. The included studies in the meta-analysis of randomized controlled trials according to the inclusion criteria of the duplicated study population

**Study name**	**First author, year**	**Follow-up duration (years)**	**MHT type**	**All-cause death**	**CVD death**	**Stroke**	**VTE**	**PE**	**CHD**	**MI**	**Angina**	**Revasculari-zation**
ESPIRIT	ESPRIT team 2002[8]	2	Estrogen		X	X	X	X				
	Cherry et al. 2014[9]	14.1	Estrogen	X								
HERS	Grady et al. 2002[11]	6.8	Combined EP			X			X	X	X	X
	Hulley et al. 2002[12]	6.8	Combined EP	X	X		X	X				
WHI I, II	Rossouw et al.2002[20]	5.2	Combined EP				X					
	Manson et al. 2003[19]	5.2	Combined EP								X	X
	Manson et al. 2013[23]	13	Estrogen/ combined EP			X		X	X	X		
	Manson et al.2017[24]	18	Estrogen/ combined EP	X	X							
	Anderson et al.2004[21]	6.8	Estrogen				X					
	Hsia et al.2006[22]	6.8	Estrogen								X	X

X, included in the meta-analysis in Table 2.

Supplementary Table S6. The included studies in the meta-analysis of observational studies according to the inclusion criteria of the duplicated study population

**Study name**	**First author, year**	**No. of total**	**Follow-up duration (years)**	**MHT type**	**All-cause death**	**CVD death**	**Stroke**	**VTE**	**PE**	**CHD**	**MI**
ALSWH	Chen et al. 2017[28]	13,715	1998-2013	Any		X					
	Wilson et al. 2019[29]	13,529	21.5	Any	X						
DaHoRS	Lokkegaard et al. 2008[32]	698,098	1995-2001	Any/estrogen /combined EP							X
	Lokkegaard et al. 2017[33]	980,003	7.9	Any/estrogen /combined EP			X				
DNS	Lokkegaard et al. 2003 (a)[35]	13,122	1993-1998	Any/estrogen /combined EP			X				
	Lokkegaard et al. 2003 (b)[36]	13,084	1993-1998	Any/estrogen /combined EP	X					X	X
ESTHER	Scarabin et al. 2003[61]	536	1999-2002	Estrogen /combined EP				△			
	Canonico et al. 2007[62]	881	1999-2005	Any				X			
GHC	Newton et al. 2003[38]	770	6.8	Any/estrogen /combined EP						X	
	Smith et al. 2004[63]	2,854	1995-2001	Any/estrogen /combined EP				X			
	Lemaitre et al. 2006[64]	6,929	1986-1989	Any/estrogen /combined EP			X				X
GPRD	Kim et al. 2006[65]	166,310	1987-2001	Any/estrogen /combined EP							X
	Tannen et al. 2007[39]	18,462	5.5	Estrogen	X				X		
	Weiner et al. 2008[40]	50,756	5	Combined EP	X				X		
	Renoux et al. 2008[58]	75,668	6.7	Any/estrogen /combined EP			X				
	Renoux et al. 2010 (a)[59]	75,668	6.7	Oral /transdermal			△				
	Renoux et al. 2010 (b)[60]	255,067	1987-2008	Any/estrogen /combined EP/oral/patch				X			

NHI	Su et al. 2012[45]	16,045	9.2	Any/estrogen /combined EP	X	X	X		X		X
	Chen et al. 2015[46]	1,712	5	Any/estrogen/oral			X				
	Lee et al. 2015[47]	358,702	2	Any				X			
	Huang et al. 2018[48]	28,860	3	Any						X	
NHS	Grodstein et al. 2000[49]	70,533	1976-1996	Any/estrogen /combined EP/oral			X			X	
	Bhupathiraju et al. 2018[50]	53,797	18	Any/estrogen /vaginal			X	X			X

X, included in the meta-analysis in Table 2; △, only included in the subgroup analysis in Table 4.

Supplementary Table S7. Risk of bias assessment for randomized controlled trials using the Jadad scale

**Trials**	**Randomization**	**Blinding**	**An account of all patient**	**Total scoring (quality)***
DOPS [1]	2	0	1	3 (fair)
EAGAR [2]	1	2	1	4 (good)
ELITE [3]	0	2	1	3 (fair)
EMS [4]	2	2	1	5 (good)
EPAT [5]	2	2	1	5 (good)
EPHT [6]	2	1	1	4 (good)
ERA [7]	2	2	1	5 (good)
ESPRIT [8,9]	2	2	1	5 (good)
Greenspan [10]	2	2	1	5 (good)
HERS [11,12]	2	2	1	5 (good)
KEEPS [13]	2	2	1	5 (good)
PHASE [14]	0	2	1	3 (fair)
STOP-IT [15]	0	2	1	3 (fair)
WAVE [16]	2	2	1	5 (good)
WELL-HART [17]	2	2	1	5 (good)
WEST [18]	2	2	1	5 (good)
WHI I [19,20,23,24]	2	2	1	5 (good)
WHI II [21-24]	2	2	1	5 (good)
WHISP [25]	1	2	0	3 (fair)
WISDOM [26]	2	2	1	5 (good)

*The thresholds for assessing quality as follows: 1) good (4-5 points), 2) fair (3 points) and 3) poor (0-2 points).

DOPS, Danish Osteoporosis Prevention Study; EAGAR, Estrogen And Graft Atherosclerosis Research; ELITE, Early versus Late Intervention Trial with Estradiol; EMS, Estrogen Memory Study; EPAT, Estrogen in Prevention Atherosclerosis Trial; EPHT, Estonian Postmenopausal Hormone Therapy; ERA, Estrogen Replacement and Atherosclerosis; ESPRIT, Estrogen for the Prevention of Re-Infarction Trial; HERS, Heart and Estrogen/Progestin Replacement Study; KEEPS, Kronos Early Estrogen Prevention Study; PHASE, Papworth HRT Atherosclerosis; WAVE, Women's Angiographic Vitamin and Estrogen; WELL-HART, Women's Estrogen Progestin Lipid-Lowering Hormone Atherosclerosis; WEST, Women's Estrogen for Stroke Trial; WHI, Women's Health Initiative; WHISP, Women's Hormone Intervention Secondary Prevention Study; WISDOM, Women's International Study of long Duration Oestrogen after Menopause.

Supplementary Table S8. Risk of bias assessment for cohort studies using the Newcastle-Ottawa Scale

**First author, year**	**Study name**	**Selection**	**Comparability**	**Outcome**	**Quality***
Apostolakis et al. 2014 [27]	AFFIRM	1	1	0	Poor
Chen et al. 2017 [28]	ALSWH	4	1	2	Good
Wilson et al. 2019[29]	ALSWH	4	2	2	Good
Alexander et al. 2001 [30]	CARS	3	2	0	Poor
Stram et al. 2011 [31]	CTS	2	2	2	Fair
Lokkegaard et al. 2008 [32]	DaHoRS	4	1	3	Good
Lokkegaard et al. 2017 [33]	DaHoRS	4	1	2	Good
Holm et al. 2019[34]	Diet, Cancer and Health	3	1	3	Good
Lokkegaard et al. 2003 (a) [35]	DNS	2	2	3	Fair
Lokkegaard et al. 2003 (b)[36]	DNS	2	2	2	Fair
Canonico et al. 2010 [37]	E3N	3	2	2	Good
Newton et al. 2003 [38]	GHC	4	2	2	Good
Tannen et al. 2007 [39]	GPRD	4	2	2	Good
Weiner et al. 2008 [40]	GPRD	4	2	2	Good
Ferrara et al. 2003 [41]	KPMCP	4	2	1	Poor
Ohira et al. 2010 [42]	LITE	4	1	2	Good
Mares et al. 2008 [43]	MISSION	4	0	2	Poor
Sweetlands et al. 2012 [44]	MWS	3	2	2	Good
Su et al. 2012 [45]	NHI	4	1	3	Good
Chen et al. 2015 [46]	NHI	4	1	2	Good
Lee et al. 2015 [47]	NHI	4	1	2	Good
Huang et al. 2018 [48]	NHI	4	1	2	Good
Grodstein et al. 2000 [49]	NHS	2	2	2	Fair
Bhupathiraju et al. 2018[50]	NHS	2	2	3	Fair
Shlipak et al. 2001 [51]	NRMI-3	1	0	1	Poor
Pentti et al. 2006 [52]	OSTRPE	4	2	2	Good
Alexandersen et al. 2006 [53]	PERF	4	2	2	Good
Ryan et al. 2012[54]	Three-City Study (3C)	4	1	2	Good
Crandall et al. 2018 [55]	WHI-OS	2	2	2	Fair
Graff-lversen et al. 2004 [56]	NR	4	1	2	Good

*Thresholds for converting the Newcastle-Ottawa Scales to Agency for Healthcare Research and Quality (AHRQ) standards (good, fair and poor); 1) Good quality: selection (3 or 4 stars) AND comparability (1 or 2 stars) AND outcome/exposure (2 or 3 stars), 2) Fair quality: selection (2 stars) AND comparability (1 or 2 stars) AND outcome/exposure (2 or 3 stars) and 3) Poor quality: selection (0 or 1 star) OR comparability (0 star) OR outcome/exposure (0 or 1 star).

AFFIRM, Atrial Fibrillation Follow-Up Investigation of Rhythm Management; ALSWH, Australian Longitudinal Study on Women's Health; CARS, The Coumadin Aspirin Reinfarction Study; CTS, The California Teachers Cohort; DaHoRS, The Danish Sex Hormone Register Study; DNS, The Danish Nurses Study; E3N, Etude Epidemiologique de femmes de l'Education Nationale; GHC, The Group Health Cooperative; GPRD, General Practice Research Database; KPMCP, Kaiser Permanente Medical Care Program; LITE, The Longitudinal Investigation of Thromboembolism Etiology; MWS, Million Women Study; NHI, National Health Insurance; NHS, Nurses' Health Study; NR, not reported; NRMI-3, National Registry of Myocardial

Infarction-3; OSTPRE, Kuopio Osteoporosis Risk Factor and Prevention; PERF, Prospective Epidemiological Risk Factors; WHI-OS, The Women's Health Initiative Observational Study.

Supplementary Table S9. Risk of bias assessment for nested case-control studies using the Newcastle-Ottawa Scale

**First author, year**	**Study name**	**Selection**	**Comparability**	**Outcome**	**Quality***
Canonico et al. 2016 [57]	French NHI	4	1	3	Good
Lee et al. 2015 [47]	NHI	4	1	2	Good
Renoux et al. 2008[58]	GPRD	4	2	3	Good
Renoux et al. 2010 (a) [59]	GPRD	4	2	3	Good
Renoux et al. 2010 (b) [60]	GPRD	4	2	3	Good

*Thresholds for converting the Newcastle-Ottawa Scales to Agency for Healthcare Research and Quality (AHRQ) standards (good, fair and poor); 1) Good quality: selection (3 or 4 stars) AND comparability (1 or 2 stars) AND outcome/exposure (2 or 3 stars), 2) Fair quality: selection (2 stars) AND comparability (1 or 2 stars) AND outcome/exposure (2 or 3 stars) and 3) Poor quality: selection (0 or 1 star) OR comparability (0 star) OR outcome/exposure (0 or 1 star).
GPRD, General Practice Research Database; NHI, National Health Insurance.

Supplementary Table S10. Risk of bias assessment for case-control studies using the Newcastle-Ottawa Scale

First author, year	Study name	Selection	Comparability	Exposure	Quality*
Scarabin et al. 2003 [61]	ESTHER	3	0	2	Poor
Canonico et al. 2007 [62]	ESTHER	3	1	3	Good
Smith et al. 2004 [63]	GHC	4	1	3	Good
Lemaitre et al. 2006 [64]	GHC	4	1	2	Good
Kim et al. 2006 [65]	GPRD	4	2	2	Good
Roach et al. 2013 [66]	MEGA	4	1	1	Poor
de Lecinana et al. 2007 [67]	PIVE	3	0	1	Poor
Carrasquilla et al. 2015 [68]	SHEEP	4	2	1	Poor
Bergendal et al. 2012 [69]	THES	4	1	2	Good
Petitti et al. 2000 [70]	NR	3	0	1	Poor
Hippisley-Cox et al. 2003 [71]	NR	4	2	3	Good
Chilvers et al. 2003 [72]	NR	4	2	3	Good
Douketis et al. 2005 [73]	NR	3	0	1	Poor

*Thresholds for converting the Newcastle-Ottawa Scales to Agency for Healthcare Research and Quality (AHRQ) standards (good, fair and poor); 1) Good quality: selection (3 or 4 stars) AND comparability (1 or 2 stars) AND outcome/exposure (2 or 3 stars), 2) Fair quality: selection (2 stars) AND comparability (1 or 2 stars) AND outcome/exposure (2 or 3 stars) and 3) Poor quality: selection (0 or 1 star) OR comparability (0 star) OR outcome/exposure (0 or 1 star).

ESTHER, The Estrogen and Thromboembolism Risk; GHC, The Group Health Cooperative; GPRD, General Practice Research Database; NR, not reported; PIVE, Protection against stroke as a function of estrogenic lifetime; SHEEP, The Stockholm Heart Epidemiology Program; TEHS, The Thrombo Embolism Hormone Study.

Reference list of included studies in the meta-analysis

1 Schierbeck, L. L. *et al.* Effect of hormone replacement therapy on cardiovascular events in recently postmenopausal women: randomised trial. *BMJ* **345**, e6409, doi:10.1136/bmj.e6409 (2012).

2 Ouyang, P. *et al.* Randomized trial of hormone therapy in women after coronary bypass surgery. Evidence of differential effect of hormone therapy on angiographic progression of disease in saphenous vein grafts and native coronary arteries. *Atherosclerosis* **189**, 375-386, doi:10.1016/j.atherosclerosis.2005.12.015 (2006).

3 Hodis, H. N. *et al.* Vascular Effects of Early versus Late Postmenopausal Treatment with Estradiol. *N Engl J Med* **374**, 1221-1231, doi:10.1056/NEJMoa1505241 (2016).

4 Tierney, M. C. *et al.* A randomized double-blind trial of the effects of hormone therapy on delayed verbal recall in older women. *Psychoneuroendocrinology* **34**, 1065-1074, doi:10.1016/j.psyneuen.2009.02.009 (2009).

5 Hodis, H. N. *et al.* Estrogen in the prevention of atherosclerosis. A randomized, double-blind, placebo-controlled trial. *Ann Intern Med* **135**, 939-953, doi:10.7326/0003-4819-135-11-200112040-00005 (2001).

6 Veerus, P. *et al.* Results from the Estonian postmenopausal hormone therapy trial [ISRCTN35338757]. *Maturitas* **55**, 162-173, doi:10.1016/j.maturitas.2006.01.012 (2006).

7 Herrington, D. M. *et al.* Effects of estrogen replacement on the progression of coronary-artery atherosclerosis. *N Engl J Med* **343**, 522-529, doi:10.1056/NEJM200008243430801 (2000).

8 Cherry, N. *et al.* Oestrogen therapy for prevention of reinfarction in postmenopausal women: a randomised placebo controlled trial. *Lancet* **360**, 2001-2008, doi:10.1016/s0140-6736(02)12001-0 (2002).

9 Cherry, N., McNamee, R., Heagerty, A., Kitchener, H. & Hannaford, P. Long-term safety of unopposed estrogen used by women surviving myocardial infarction: 14-year follow-up of the ESPRIT randomised controlled trial. *BJOG* **121**, 700-705; discussion 705, doi:10.1111/1471-0528.12598 (2014).

10 Greenspan, S. L., Resnick, N. M. & Parker, R. A. The effect of hormone replacement on physical performance in community-dwelling elderly women. *Am J Med* **118**, 1232-1239, doi:10.1016/j.amjmed.2005.03.004 (2005).

11 Grady, D. *et al.* Cardiovascular disease outcomes during 6.8 years of hormone therapy: Heart and Estrogen/progestin Replacement Study follow-up (HERS II). *JAMA* **288**, 49-57,

doi:10.1001/jama.288.1.49 (2002).

12 Hulley, S. *et al.* Noncardiovascular disease outcomes during 6.8 years of hormone therapy: Heart and Estrogen/progestin Replacement Study follow-up (HERS II). *JAMA* **288**, 58-66, doi:10.1001/jama.288.1.58 (2002).

13 Gleason, C. E. *et al.* Effects of Hormone Therapy on Cognition and Mood in Recently Postmenopausal Women: Findings from the Randomized, Controlled KEEPS-Cognitive and Affective Study. *PLoS Med* **12**, e1001833; discussion e1001833, doi:10.1371/journal.pmed.1001833 (2015).

14 Clarke, S. C., Kelleher, J., Lloyd-Jones, H., Slack, M. & Schofiel, P. M. A study of hormone replacement therapy in postmenopausal women with ischaemic heart disease: the Papworth HRT atherosclerosis study. *BJOG* **109**, 1056-1062 (2002).

15 Gallagher, J. C., Fowler, S. E., Detter, J. R. & Sherman, S. S. Combination treatment with estrogen and calcitriol in the prevention of age-related bone loss. *J Clin Endocrinol Metab* **86**, 3618-3628, doi:10.1210/jcem.86.8.7703 (2001).

16 Waters, D. D. *et al.* Effects of hormone replacement therapy and antioxidant vitamin supplements on coronary atherosclerosis in postmenopausal women: a randomized controlled trial. *JAMA* **288**, 2432-2440, doi:10.1001/jama.288.19.2432 (2002).

17 Hodis, H. N. *et al.* Hormone therapy and the progression of coronary-artery atherosclerosis in postmenopausal women. *N Engl J Med* **349**, 535-545, doi:10.1056/NEJMoa030830 (2003).

18 Viscoli, C. M. *et al.* A clinical trial of estrogen-replacement therapy after ischemic stroke. *N Engl J Med* **345**, 1243-1249, doi:10.1056/NEJMoa010534 (2001).

19 Manson, J. E. *et al.* Estrogen plus progestin and the risk of coronary heart disease. *N Engl J Med* **349**, 523-534, doi:10.1056/NEJMoa030808 (2003).

20 Rossouw, J. E. *et al.* Risks and benefits of estrogen plus progestin in healthy postmenopausal women: principal results From the Women's Health Initiative randomized controlled trial. *JAMA* **288**, 321-333, doi:10.1001/jama.288.3.321 (2002).

21 Anderson, G. L. *et al.* Effects of conjugated equine estrogen in postmenopausal women with hysterectomy: the Women's Health Initiative randomized controlled trial. *JAMA* **291**, 1701-1712, doi:10.1001/jama.291.14.1701 (2004).

22 Hsia, J. *et al.* Conjugated equine estrogens and coronary heart disease: the Women's Health Initiative.

Arch Intern Med **166**, 357-365, doi:10.1001/archinte.166.3.357 (2006).

23 Manson, J. E. *et al.* Menopausal hormone therapy and health outcomes during the intervention and extended poststopping phases of the Women's Health Initiative randomized trials. *JAMA* **310**, 1353-1368, doi:10.1001/jama.2013.278040 (2013).

24 Manson, J. E. *et al.* Menopausal Hormone Therapy and Long-term All-Cause and Cause-Specific Mortality: The Women's Health Initiative Randomized Trials. *JAMA* **318**, 927-938, doi:10.1001/jama.2017.11217 (2017).

25 Collins, P. *et al.* Randomized trial of effects of continuous combined HRT on markers of lipids and coagulation in women with acute coronary syndromes: WHISP Pilot Study. *Eur Heart J* **27**, 2046-2053, doi:10.1093/eurheartj/ehl183 (2006).

26 Vickers, M. R. *et al.* Main morbidities recorded in the women's international study of long duration oestrogen after menopause (WISDOM): a randomised controlled trial of hormone replacement therapy in postmenopausal women. *BMJ* **335**, 239, doi:10.1136/bmj.39266.425069.AD (2007).

27 Apostolakis, S., Sullivan, R. M., Olshansky, B. & Lip, G. Y. Hormone replacement therapy and adverse outcomes in women with atrial fibrillation: an analysis from the atrial fibrillation follow-up investigation of rhythm management trial. *Stroke* **45**, 3076-3079, doi:10.1161/STROKEAHA.114.006668 (2014).

28 Chen, L., Mishra, G. D., Dobson, A. J., Wilson, L. F. & Jones, M. A. Protective effect of hormone therapy among women with hysterectomy/oophorectomy. *Hum Reprod* **32**, 885-892, doi:10.1093/humrep/dex017 (2017).

29 Wilson, L. F., Pandeya, N., Byles, J. & Mishra, G. D. Hysterectomy status and all-cause mortality in a 21-year Australian population-based cohort study. *Am J Obstet Gynecol* **220**, 83 e81-83 e11, doi:10.1016/j.ajog.2018.10.002 (2019).

30 Alexander, K. P. *et al.* Initiation of hormone replacement therapy after acute myocardial infarction is associated with more cardiac events during follow-up. *J Am Coll Cardiol* **38**, 1-7, doi:10.1016/s0735-1097(01)01329-8 (2001).

31 Stram, D. O. *et al.* Age-specific effects of hormone therapy use on overall mortality and ischemic heart disease mortality among women in the California Teachers Study. *Menopause* **18**, 253-261, doi:10.1097/gme.0b013e3181f0839a (2011).

32 Lokkegaard, E. *et al.* Hormone therapy and risk of myocardial infarction: a national register study. *Eur*

Heart J **29**, 2660-2668, doi:10.1093/eurheartj/ehn408 (2008).

33 Lokkegaard, E., Nielsen, L. H. & Keiding, N. Risk of Stroke With Various Types of Menopausal Hormone Therapies: A National Cohort Study. *Stroke* **48**, 2266-2269, doi:10.1161/STROKEAHA.117.017132 (2017).

34 Holm, M. *et al.* Pattern of mortality after menopausal hormone therapy: long-term follow up in a population-based cohort. *BJOG* **126**, 55-63, doi:10.1111/1471-0528.15433 (2019).

35 Lokkegaard, E. *et al.* Increased risk of stroke in hypertensive women using hormone therapy: analyses based on the Danish Nurse Study. *Arch Neurol* **60**, 1379-1384, doi:10.1001/archneur.60.10.1379 (2003).

36 Lokkegaard, E. *et al.* Relation between hormone replacement therapy and ischaemic heart disease in women: prospective observational study. *BMJ* **326**, 426, doi:10.1136/bmj.326.7386.426 (2003).

37 Canonico, M. *et al.* Postmenopausal hormone therapy and risk of idiopathic venous thromboembolism: results from the E3N cohort study. *Arterioscler Thromb Vasc Biol* **30**, 340-345, doi:10.1161/ATVBAHA.109.196022 (2010).

38 Newton, K. M. *et al.* Estrogen therapy and risk of cardiovascular events among women with type 2 diabetes. *Diabetes Care* **26**, 2810-2816, doi:10.2337/diacare.26.10.2810 (2003).

39 Tannen, R. L., Weiner, M. G., Xie, D. & Barnhart, K. Estrogen affects post-menopausal women differently than estrogen plus progestin replacement therapy. *Hum Reprod* **22**, 1769-1777, doi:10.1093/humrep/dem031 (2007).

40 Weiner, M. G., Barnhart, K., Xie, D. & Tannen, R. L. Hormone therapy and coronary heart disease in young women. *Menopause* **15**, 86-93, doi:10.1097/gme.0b013e3180413e45 (2008).

41 Ferrara, A. *et al.* Current use of unopposed estrogen and estrogen plus progestin and the risk of acute myocardial infarction among women with diabetes: the Northern California Kaiser Permanente Diabetes Registry, 1995-1998. *Circulation* **107**, 43-48, doi:10.1161/01.cir.0000042701.17528.95 (2003).

42 Ohira, T. *et al.* Reproductive history, hormone replacement, and incidence of venous thromboembolism: the Longitudinal Investigation of Thromboembolism Etiology. *Br J Haematol* **149**, 606-612, doi:10.1111/j.1365-2141.2010.08128.x (2010).

43 Mares, P. *et al.* Coronary heart disease and HRT in France: MISSION study prospective phase results. *Gynecol Endocrinol* **24**, 696-700, doi:10.1080/09513590802454935 (2008).

44 Sweetland, S. *et al.* Venous thromboembolism risk in relation to use of different types of postmenopausal

hormone therapy in a large prospective study. *J Thromb Haemost* **10**, 2277-2286, doi:10.1111/j.1538-7836.2012.04919.x (2012).

45 Su, I. H. *et al.* Risks and benefits of menopausal hormone therapy in postmenopausal Chinese women. *Menopause* **19**, 931-941, doi:10.1097/gme.0b013e31824362ff (2012).

46 Chen, Y. H., Hsieh, T. F., Lee, C. C., Wu, M. J. & Fu, Y. C. Estrogen Therapy and Ischemic Stroke in Women with Diabetes Aged Over 55 Years: A Nation-Wide Prospective Population-Based Study in Taiwan. *PLoS One* **10**, e0144910, doi:10.1371/journal.pone.0144910 (2015).

47 Lee, C. H., Cheng, C. L., Kao Yang, Y. H. & Lin, L. J. Hormone therapy and risk of venous thromboembolism among postmenopausal women in Taiwan - a 10-year nationwide population-based study. *Circ J* **79**, 1107-1114, doi:10.1253/circj.CJ-14-1227 (2015).

48 Huang, C. H., Li, C. L., Kor, C. T. & Chang, C. C. Menopausal symptoms and risk of coronary heart disease in middle-aged women: A nationwide population-based cohort study. *PLoS One* **13**, e0206036, doi:10.1371/journal.pone.0206036 (2018).

49 Grodstein, F. *et al.* A prospective, observational study of postmenopausal hormone therapy and primary prevention of cardiovascular disease. *Ann Intern Med* **133**, 933-941, doi:10.7326/0003-4819-133-12-200012190-00008 (2000).

50 Bhupathiraju, S. N. *et al.* Vaginal estrogen use and chronic disease risk in the Nurses' Health Study. *Menopause* **26**, 603-610, doi:10.1097/GME.0000000000001284 (2018).

51 Shlipak, M. G. *et al.* Hormone therapy and in-hospital survival after myocardial infarction in postmenopausal women. *Circulation* **104**, 2300-2304, doi:10.1161/hc4401.98414 (2001).

52 Pentti, K. *et al.* Hormone replacement therapy and mortality in 52- to 70-year-old women: the Kuopio Osteoporosis Risk Factor and Prevention Study. *Eur J Endocrinol* **154**, 101-107, doi:10.1530/eje.1.02059 (2006).

53 Alexandersen, P., Tanko, L. B., Bagger, Y. Z., Qin, G. & Christiansen, C. The long-term impact of 2-3 years of hormone replacement therapy on cardiovascular mortality and atherosclerosis in healthy women. *Climacteric* **9**, 108-118, doi:10.1080/13697130600647743 (2006).

54 Ryan, J. *et al.* Hormone treatment, estrogen receptor polymorphisms and mortality: a prospective cohort study. *PLoS One* **7**, e34112, doi:10.1371/journal.pone.0034112 (2012).

55 Crandall, C. J. *et al.* Breast cancer, endometrial cancer, and cardiovascular events in participants who

used vaginal estrogen in the Women's Health Initiative Observational Study. *Menopause* **25**, 11-20, doi:10.1097/GME.0000000000000956 (2018).

56 Graff-Iversen, S., Hammar, N., Thelle, D. S. & Tonstad, S. Hormone therapy and mortality during a 14-year follow-up of 14 324 Norwegian women. *J Intern Med* **256**, 437-445, doi:10.1111/j.1365-2796.2004.01396.x (2004).

57 Canonico, M. *et al.* Postmenopausal Hormone Therapy and Risk of Stroke: Impact of the Route of Estrogen Administration and Type of Progestogen. *Stroke* **47**, 1734-1741, doi:10.1161/STROKEAHA.116.013052 (2016).

58 Renoux, C., Dell'aniello, S., Garbe, E. & Suissa, S. Hormone replacement therapy use and the risk of stroke. *Maturitas* **61**, 305-309, doi:10.1016/j.maturitas.2008.09.020 (2008).

59 Renoux, C., Dell'aniello, S., Garbe, E. & Suissa, S. Transdermal and oral hormone replacement therapy and the risk of stroke: a nested case-control study. *BMJ* **340**, c2519, doi:10.1136/bmj.c2519 (2010).

60 Renoux, C., Dell'Aniello, S. & Suissa, S. Hormone replacement therapy and the risk of venous thromboembolism: a population-based study. *J Thromb Haemost* **8**, 979-986, doi:10.1111/j.1538-7836.2010.03839.x (2010).

61 Scarabin, P. Y., Oger, E., Plu-Bureau, G., Estrogen & Group, T. H. R. S. Differential association of oral and transdermal oestrogen-replacement therapy with venous thromboembolism risk. *Lancet* **362**, 428-432, doi:10.1016/S0140-6736(03)14066-4 (2003).

62 Canonico, M. *et al.* Hormone therapy and venous thromboembolism among postmenopausal women: impact of the route of estrogen administration and progestogens: the ESTHER study. *Circulation* **115**, 840-845, doi:10.1161/CIRCULATIONAHA.106.642280 (2007).

63 Smith, N. L. *et al.* Esterified estrogens and conjugated equine estrogens and the risk of venous thrombosis. *JAMA* **292**, 1581-1587, doi:10.1001/jama.292.13.1581 (2004).

64 Lemaitre, R. N. *et al.* Esterified estrogen and conjugated equine estrogen and the risk of incident myocardial infarction and stroke. *Arch Intern Med* **166**, 399-404, doi:10.1001/archinte.166.4.399 (2006).

65 Kim, J., Evans, S., Smeeth, L. & Pocock, S. Hormone replacement therapy and acute myocardial infarction: a large observational study exploring the influence of age. *Int J Epidemiol* **35**, 731-738, doi:10.1093/ije/dyi285 (2006).

66 Roach, R. E. *et al.* The risk of venous thrombosis in women over 50 years old using oral contraception

or postmenopausal hormone therapy. *J Thromb Haemost* **11**, 124-131, doi:10.1111/jth.12060 (2013).

67 Alonso de Lecinana, M. *et al.* Risk of ischemic stroke and lifetime estrogen exposure. *Neurology* **68**, 33-38, doi:10.1212/01.wnl.0000250238.69938.f5 (2007).

68 Carrasquilla, G. D. *et al.* Does menopausal hormone therapy reduce myocardial infarction risk if initiated early after menopause? A population-based case-control study. *Menopause* **22**, 598-606, doi:10.1097/GME.0000000000000354 (2015).

69 Bergendal, A. *et al.* Risk factors for venous thromboembolism in pre-and postmenopausal women. *Thromb Res* **130**, 596-601, doi:10.1016/j.thromres.2012.05.024 (2012).

70 Petitti, D. B., Sidney, S. & Quesenberry, C. P., Jr. Hormone replacement therapy and the risk of myocardial infarction in women with coronary risk factors. *Epidemiology* **11**, 603-606, doi:10.1097/00001648-200009000-00018 (2000).

71 Hippisley-Cox, J., Pringle, M., Crown, N. & Coupland, C. A case-control study on the effect of hormone replacement therapy on ischaemic heart disease. *Br J Gen Pract* **53**, 191-196 (2003).

72 Chilvers, C. E., Knibb, R. C., Armstrong, S. J., Woods, K. L. & Logan, R. F. Post menopausal hormone replacement therapy and risk of acute myocardial infarction--a case control study of women in the East Midlands, UK. *Eur Heart J* **24**, 2197-2205, doi:10.1016/j.ehj.2003.09.019 (2003).

73 Douketis, J. D. *et al.* Does the type of hormone replacement therapy influence the risk of deep vein thrombosis? A prospective case-control study. *J Thromb Haemost* **3**, 943-948, doi:10.1111/j.1538-7836.2005.01268.x (2005).
